# REEV: review, evaluate and explain variants

**DOI:** 10.1093/nar/gkae366

**Published:** 2024-05-20

**Authors:** Dzmitry Hramyka, Henrike Lisa Sczakiel, Max Xiaohang Zhao, Oliver Stolpe, Mikko Nieminen, Ronja Adam, Magdalena Danyel, Lara Einicke, René Hägerling, Alexej Knaus, Stefan Mundlos, Sarina Schwartzmann, Dominik Seelow, Nadja Ehmke, Martin Atta Mensah, Felix Boschann, Dieter Beule, Manuel Holtgrewe

**Affiliations:** Berlin Institute of Health, Core Unit Bioinformatics, Berlin, Germany; Institute of Medical Genetics and Human Genetics, Charité - Universitätsmedizin Berlin, Freie Universität Berlin and Humboldt-Universität zu Berlin, Berlin, Germany; BIH Biomedical Innovation Academy, Clinician Scientist Program, Berlin Institute of Health at Charité - Universitätsmedizin Berlin, Berlin, Germany; RG Development & Disease, Max Planck Institute for Molecular Genetics, Berlin, Germany; Berlin Institute of Health, Core Unit Bioinformatics, Berlin, Germany; Institute of Medical Genetics and Human Genetics, Charité - Universitätsmedizin Berlin, Freie Universität Berlin and Humboldt-Universität zu Berlin, Berlin, Germany; Berlin Institute of Health, Berlin, Germany; Berlin Institute of Health, Core Unit Bioinformatics, Berlin, Germany; Berlin Institute of Health, Core Unit Bioinformatics, Berlin, Germany; Institute of Medical Genetics and Human Genetics, Charité - Universitätsmedizin Berlin, Freie Universität Berlin and Humboldt-Universität zu Berlin, Berlin, Germany; Berlin Institute of Health, Berlin, Germany; Institute of Medical Genetics and Human Genetics, Charité - Universitätsmedizin Berlin, Freie Universität Berlin and Humboldt-Universität zu Berlin, Berlin, Germany; BIH Biomedical Innovation Academy, Clinician Scientist Program, Berlin Institute of Health at Charité - Universitätsmedizin Berlin, Berlin, Germany; Institute of Medical Genetics and Human Genetics, Charité - Universitätsmedizin Berlin, Freie Universität Berlin and Humboldt-Universität zu Berlin, Berlin, Germany; Berlin Institute of Health, Berlin, Germany; Institute of Medical Genetics and Human Genetics, Charité - Universitätsmedizin Berlin, Freie Universität Berlin and Humboldt-Universität zu Berlin, Berlin, Germany; BIH Biomedical Innovation Academy, Clinician Scientist Program, Berlin Institute of Health at Charité - Universitätsmedizin Berlin, Berlin, Germany; RG Development & Disease, Max Planck Institute for Molecular Genetics, Berlin, Germany; Berlin Institute of Health , BIH Center for Regenerative Therapies, Berlin, Germany; Institute for Genomic Statistics and Bioinformatics, University Hospital Bonn, Germany; Institute of Medical Genetics and Human Genetics, Charité - Universitätsmedizin Berlin, Freie Universität Berlin and Humboldt-Universität zu Berlin, Berlin, Germany; RG Development & Disease, Max Planck Institute for Molecular Genetics, Berlin, Germany; Institute of Medical Genetics and Human Genetics, Charité - Universitätsmedizin Berlin, Freie Universität Berlin and Humboldt-Universität zu Berlin, Berlin, Germany; Institute of Medical Genetics and Human Genetics, Charité - Universitätsmedizin Berlin, Freie Universität Berlin and Humboldt-Universität zu Berlin, Berlin, Germany; Berlin Institute of Health, Berlin, Germany; Institute of Medical Genetics and Human Genetics, Charité - Universitätsmedizin Berlin, Freie Universität Berlin and Humboldt-Universität zu Berlin, Berlin, Germany; Berlin Institute of Health, Berlin, Germany; Institute of Medical Genetics and Human Genetics, Charité - Universitätsmedizin Berlin, Freie Universität Berlin and Humboldt-Universität zu Berlin, Berlin, Germany; BIH Biomedical Innovation Academy, Digital Clinician Scientist Program, Berlin Institute of Health at Charité - Universitätsmedizin Berlin, Berlin, Germany; Institute of Medical Genetics and Human Genetics, Charité - Universitätsmedizin Berlin, Freie Universität Berlin and Humboldt-Universität zu Berlin, Berlin, Germany; BIH Biomedical Innovation Academy, Clinician Scientist Program, Berlin Institute of Health at Charité - Universitätsmedizin Berlin, Berlin, Germany; Berlin Institute of Health, Berlin, Germany; Berlin Institute of Health, Core Unit Bioinformatics, Berlin, Germany; Max Delbrück Center for Molecular Medicine in the Helmholtz Association, Berlin, Germany; Berlin Institute of Health, Core Unit Bioinformatics, Berlin, Germany

## Abstract

In the era of high throughput sequencing, special software is required for the clinical evaluation of genetic variants. We developed REEV (Review, Evaluate and Explain Variants), a user-friendly platform for clinicians and researchers in the field of rare disease genetics. Supporting data was aggregated from public data sources. We compared REEV with seven other tools for clinical variant evaluation. REEV (semi-)automatically fills individual ACMG criteria facilitating variant interpretation. REEV can store disease and phenotype data related to a case to use these for phenotype similarity measures. Users can create public permanent links for individual variants that can be saved as browser bookmarks and shared. REEV may help in the fast diagnostic assessment of genetic variants in a clinical as well as in a research context. REEV (https://reev.bihealth.org/) is free and open to all users and there is no login requirement.

## Graphical Abstract

**Graphical Abstract:**
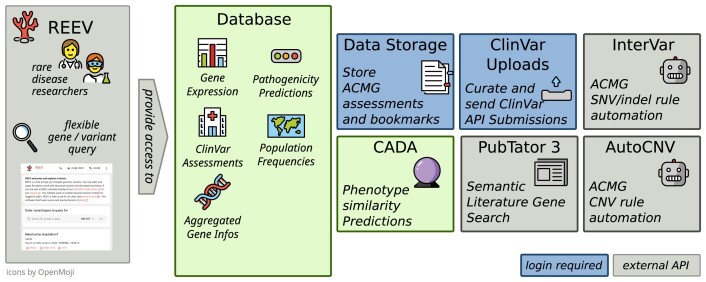

## Introduction

In recent years, high throughput genetic testing has fundamentally changed the diagnostic paradigm in clinical genetics (1,2). Hitherto, clinical geneticists were testing a very limited number of genes selected depending on a patient's phenotype. If a variant with severe consequences was found in the patients but not in healthy controls, it was likely deemed pathogenic. However, by now access to high throughput genetic analyses such as exome sequencing (ES) or genome sequencing (GS) could be implemented in standard health care (3,4). Accordingly, clinical geneticists have been faced with new challenges. One is, they now have to interpret a considerably larger number of variants from screening assays, hoping to identify the underlying disease-causing mutation among them (5,6). Specialized software is required to achieve this goal. Such tools for variant prioritization usually use two sources of information: variant-specific (molecular, evolutionary and population genetic) features and gene-to-phenotype associations (7–9). However, collecting, connecting and integrating these data about a variant of interest from multiple online sources is time consuming and individual tools frequently lack an easily accessible and comprehensive output (10,11). Also, existing platforms for gathering gene and variant information often fall short by either missing critical data or predictions, or by restricting access to these features to their commercial, full-version offerings (10,12–14). In particular, while several tools exist for the analysis and interpretation of small coding variants, albeit with the limitations mentioned above, there are currently only few platforms for the analysis and evaluation of structural variants (15). Finally, critical to the process of clinical variant evaluation is a robust final assessment of the variant's pathogenicity. To this end, the American College of Medical Genetics and Genomics (ACMG) has developed standardized guidelines for the classification of both sequence and structural variants (16,17). Although there are tools that offer individual or automated ACMG classification, an easy-to-use tool that integrates all these needs in a single platform is lacking.

Here, we introduce such a tool for the analysis, interpretation and ACMG classification of both small and structural variants. REEV (Review, Evaluate and Explain Variants; https://reev.bihealth.org/) provides an automated classification proposal, which can be easily understood, adapted, and amended. We also aim to compare REEV with other available web tools for clinical variant evaluation.

## Method outline

All components of the REEV backend pipeline including REEV’s specific original code, as well as components using or referring to third-party software and services (Table 1) are free to use in academic and commercial settings (open source, MIT license). All code, tests and data required to run REEV can be found at https://github.com/bihealth/reev. A comprehensive user documentation including a quickstart and a tutorial can be found at https://reev.readthedocs.io/. REEV is technically designed to speed-up diagnostic variant evaluation and classification in both research and clinical settings. We offer REEV as a complete open-source software suite including comprehensive automated tests as well as deployment scripts following FAIR4RS principles. Thus, REEV can also be individually extended and tailored to the specific needs of the laboratory or clinical institution using it. In several jurisdictions, software used for clinical diagnostics needs to be certified in accordance with national legal regulations. Any laboratory or institution using REEV should be aware that REEV has not been formally certified for diagnostic use in a clinical setting. Responsibility to use REEV or derivatives of REEV in a clinical setting and certify them for clinical use lies with the using institutions.

**Table 1.** Overview of sources and tools integrated with REEV. The REEV backend integrates state-of-the-art databases containing gene and variant related information including condition/disease/phenotype related information, population frequencies, pathogenicity predictions as well as expression and cross species conservation

	Data	Source
**Databases**	Seqvar frequency	gnomAD (gnomad.broadinstitute.org) (6,30)
	Variant and Gene Scores / Predictions	Variantscores: CADD (57), SpliceAI (58), and many scores from dbNSFP (45), e.g. REVEL (59) Gene constraints, e.g. gnomAD pLI/LOEUF/Z-Score (6,30) ClinGen haploinsufficiency and triplosensitivity scores (31) DECIPHER haploinsufficiency and triplosensitivity scores (28)
	Gene Information	HGNC (https://www.genenames.org/) NCBI Entrez (http://www.ncbi.nlm.nih.gov/Entrez/) (27) NCBI Gene (https://www.ncbi.nlm.nih.gov/gene/) (27)
	Gene-Condition	OMIM (26) OrphaData (Orphanet) (32) PanelApp (33) MONDO (60) HPO (21–23)
	Gene Expression	GTEx (34)
	Clinical databases	ClinVar (35) ClinGen (31) DECIPHER (28)
**Internal Services**	Annonars Mehari Viguno Cada-prio Dotty	https://github.com/varfish-org/annonars https://github.com/varfish-org/mehari https://github.com/varfish-org/viguno https://github.com/varfish-org/cada-prio https://github.com/bihealth/dotty
**Interface with**	ACMG criteria seqvar	InterVar (52)
	ACMG criteria strucvar	AutoCNV (53)
	Phenotype Similarity	CADA (24) and Exomiser (7)
	PubMed	PubTator 3 API (36,37)
**Link-Outs**	Various	See user manual for further details

REEV frontend allows to interface with semi-automated ACMG scoring tools, phenotype similarity applications and pubmed interfaces. To further support variant evaluation, validation and interpretation REEV also links out to many additional external resources, e.g. to the GA4GH beacon network, VariantValidator or MutationTaster. Current versions of the sources and tools integrated with REEV can be found at https://reev.cubi.bihealth.org/info#data-versions.

### Data storage and preprocessing

On the server, static data is stored in files. Smaller datasets are stored in text files or compressed binary Protocol Buffers format and loaded into memory on startup. To allow for reduced on-disk storage, low main memory footprint and fast lookup, larger datasets are generally stored in RocksDB (an embedded key/value store).

All data is downloaded from public sources using a Snakemake (18,19) workflow (s. also *Data Availability*). The resulting files are publicly available from our S3 server (see also *Data availability*).

### Software architecture and backend

REEV is designed to ensure full transparency and reproducibility of variant analysis in concordance with the FAIR4RS principles (20) and enable timely updates from quickly evolving data sources. REEV is actively maintained with updated releases scheduled once a quarter.

The overall architecture is a typical ‘microservice based’ web application, as roughly depicted in the graphical abstract. The ‘REEV’ server is a web server consisting of two layers. The actual user interface consists of a TypeScript/Vue single-page app (SPA) front-end (served through the web server) and a Python/FastAPI based backend for the SPA. The FastAPI server provides functionality for user login and persisting/managing data of logged-in users in a PostgreSQL database. It also functions as a reverse proxy to a number of backend services that provide the actual data and functionality.

These ‘microservices’ are: *Annonars* provides access to data on genes and variants which are stored in RocksDB databases. *Mehari* provides transcript-based variant consequence annotations (i.e. to project a genomic variant to the transcript-level and compute whether the variant leads to a missense or frameshift variant at protein level). *Viguno* provides access to HPO terms (21–23), a full-text index thereof, and standard ontology algorithms based on information content. The services above are implemented in the Rust programming language. The *cada-prio* service provides phenotype similarity queries based on the CADA (24) algorithm. The *dotty* service provides functionality for transforming variant descriptions between different notation systems, including HGVS (25), using the *hgvs* Python package (26). These two services are implemented in the Python programming language.

These microservices are running (together with utility services such as *nginx*, *traefik*, *PostgreSQL*, *Redis*, and *RabbitMQ*) in Docker containers and orchestrated with Docker Compose.

### Frontend overview

In the following, we focus on the features of the software from the perspective of the user.

REEV allows for the search of genes as well as sequence and structural variants. A short display of supported input styles is shown on the REEV startpage, while a quickstart and full tutorial on how to navigate REEV are provided in the REEV documentation available at https://reev.readthedocs.io. Here, we summarize the features implemented in REEV. For a complete list of integrated sources see Table 1. An overview of the REEV frontend for the query of sequence and structural variants can also be found in Supplementary Figures S1 and S2, respectively.

### Genes

At first, REEV provides basic information about a selected gene, including a short summary from NCBI Gene (27), a list of and link-outs to alternative identifiers for this gene, and link-outs to useful resources on gene level, e.g. DECIPHER (28), OMIM (29), pubmed (27), etc. If applicable for the gene of interest, REEV offers links to locus-specific databases, and NCBI’s *References Into Functions (RIFs)*. Next, REEV gives an overview about the gene's potential pathogenicity by providing haploinsufficiency and triplosensitivity scores, e.g. ClinGen (30) DECIPHER (28), gnomAD pLI and pLOEUF (6,30). The user is shown information about associated phenotypes (HPO terms (21–23)) and diseases (OMIM (29), Orphanet (32), Genomics England PanelApp (33)). In the case of a gene with an — as of yet — unknown disease association, REEV also displays gene expression data from the GTEx project (34). Also, REEV offers aggregated ClinVar variant statistics (35). This includes a summary of variant counts, a visualization of variant population frequencies separated by their ACMG class (16), and a plot of the variants’ positions and their ACMG classes. Finally, REEV summarizes available information of the gene of interest from the literature providing the ten most relevant hits from PubTator3 (36,37); (full data are available via link-outs to PubTator3 and PubMed).

### Sequence variants

When querying a sequence variant, REEV first provides the above-mentioned details on the respective gene. Following this gene information, the user is presented with a table showing the impact of the variant on different transcripts (according to NCBI GenBank (27,38)). REEV also provides an overview about the variant's ClinVar (35) entries, including whether the variant is present, its respective ClinVar reference assertion, its most pathogenic significance, and its review status. Users can also fold out this ClinVar card and look at the individual reference assertion. Furthermore, in this section REEV provides gnomAD v4 (6) population frequencies of the queried variant in different populations as well as the different sexes (XX vs. XY genotype) and the variant's UCSC 100 vertebrate conservation on protein level (39). Additionally, link-outs to genome browsers (ENSEMBL (40), UCSC (41)) and various external tools (DGV (42), Genoox Franklin (franklin.genoox.com), gnomAD (6,30), MutationTaster (43,44), Varsome (12)) help the user to further assess their variant of interest. REEV not only comes with an integrated display of variant pathogenicity scores from different tools (aggregated by dbNSFP (45)) but also provides a color coded suggestion of the ACMG criterion PP3 as well as raw pathogenicity scores calibrated following Pejaver et al. (46) (see also*ACMG classification*). Finally, users can query the GA4GH Beacon network (47) for entries of the variant by other institutions. Users may also submit the variant to VariantValidator (48) to obtain gold standard HGVS representation. We provide an example query for the sequence variant chr7:42012159:T:G (i.e. GLI3(NM_000168.6):c.1880A>C, p.(His627Pro)) using REEV in Figure 2, and online in our REEV tutorial (reev.readthedocs.io).

### Structural variants

Besides sequence variants, REEV also allows users to examine structural variants. These can be provided either in gnomAD (6,30) (GRCh37 or GRCh38 coordinates) or ISCN style (49). As for the sequence variants, REEV first shows general information on the affected gene followed by variant specific information. Here, REEV depicts an overview of the affected genes in the form of a list of genes that are overlapping with or are close to the structural variant. REEV displays how the variant affects every gene (i.e. gene fully or only partially contained; breakpoints exonic, intronic or extragenic). In the case of multiple overlapping genes, users may sort this list of genes by, e.g. gnomAD (6,30) pLI score or the ClinGen (31) haploinsufficiency or triplosensitivity assessment. REEV also depicts details on overlapping variants in ClinVar (35) and the individual reference ClinVar assertions. ClinVar variants will be sorted by reciprocal overlap (the fraction of overlap between the variant studied and the ClinVar variant). Users can see the genomic location of the variant in an integrated IGV genome browser (50) with tracks for interpreting the variant. Users are again provided with link-outs to known external genome browsers (ENSEMBL (40), UCSC (41)) and other external tools (DGV (42), Genoox Franklin (franklin.genoox.com), gnomAD (6,30), Varsome (12)) for further analysis. For an example query in REEV invoking the structural variant DEL:chr14:37131998:37133815, see also Figure 3 as well as the REEV tutorial (reev.readthedocs.io).

### ACMG classification

**Figure 1.**
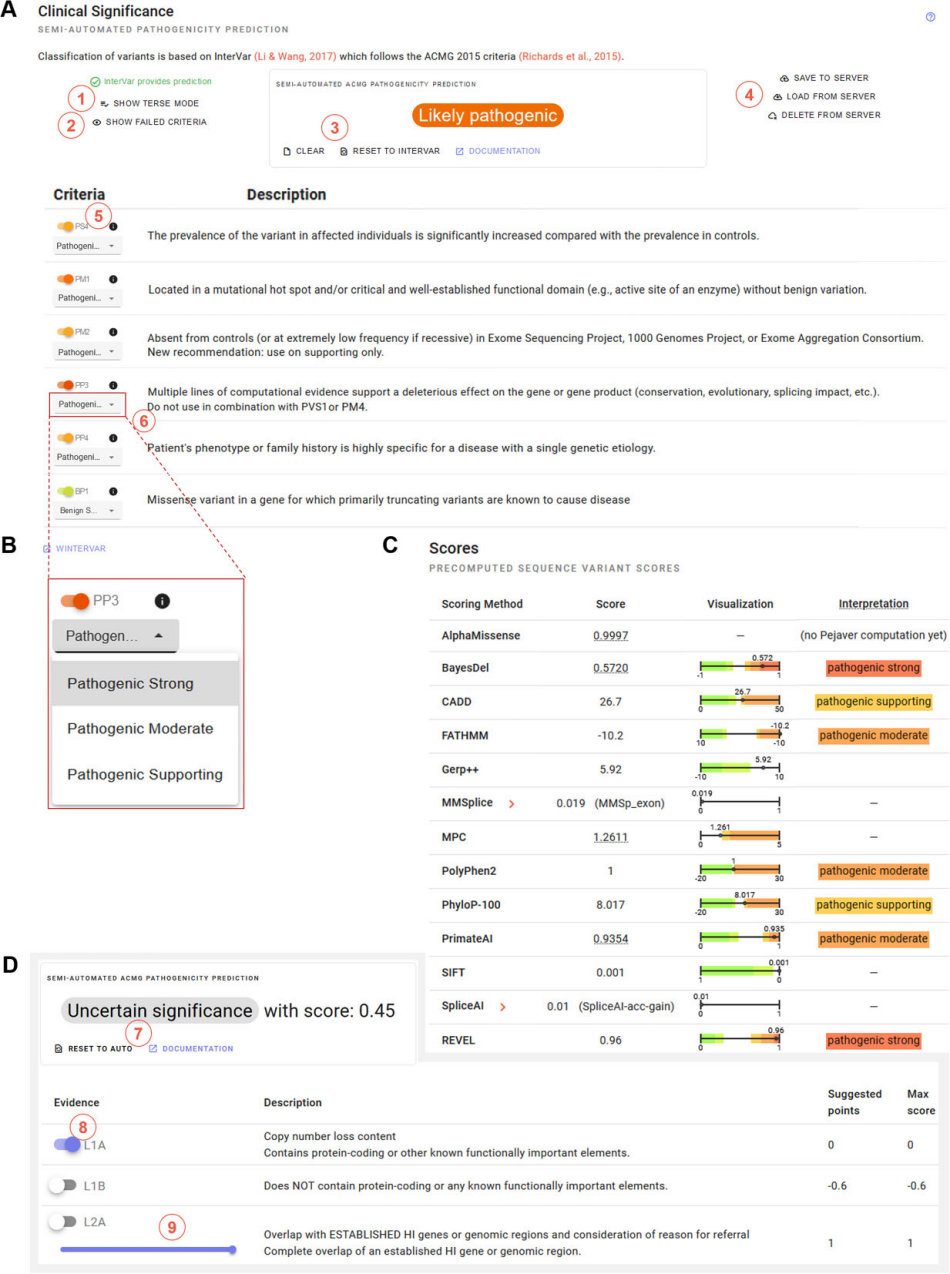
Semi-automated ACMG Classification. Overview of semi-automated variant classification using REEV. (A–C) ACMG classification of a sequence variant. (**A**) Semi-automated classification based on the InterVar tool. The user can (de)activate a terse mode (**1**), show or hide failed ACMG criteria (**2**) and clear or reset chosen criteria to auto (**3**) meaning they are computed rather than user set. Logged in users can also load and save the classification of variants (**4**). Every criterion can be (de)selected manually (**5**) and set at the chosen level of confidence (**6**). (**B**) Example of this manual setting of the level of confidence for the variable pathogenicity prediction criterion PP3. (**C**) Overview of variant pathogenicity scores from different tools (aggregated by dbNSFP) as well as color coding and calibrated scores where applicable for an easy and correct usage of the modified PP3 criterion following Pejaver *et al.* (46). (**D**) Semi-automated ACMG classification of a structural variant based on AutoCNV (applicable for ACMG/ClinGen CNV criteria 1–3 of 5 (17)), which can also be reset (**7**), (de)selected manually (**8**) and set at the chosen level of confidence (**9**).

REEV provides tools for the fast interpretation of sequence and structural variants using the ACMG guidelines for variant interpretation including commonly used modifications. For semi-automated variant classification, REEV considers three rule systems: the original ACMG 2015 guidelines (16), the ACGS 2020 rules (17) and the 2020 point system described by Tavtigian *et al.* (51). REEV offers a short explanation of the definition and application of every of their criteria. For the classification of sequence variants, REEV uses a semi-automated ACMG variant class assessment based on the InterVar (52) tool (Figure 1A, see also below for details). Users can modify and complete this by checking and unchecking every single criterion or altering its level of evidence (supporting, moderate, strong, or very strong), clear all criteria or reset to auto-fill (Figure 1A, B). Users can customize the application of the PP3-criterion (e.g. adapt the level of evidence according to the applied pathogenicity prediction tool) (Figure 1C). Semi-automated classification of structural variants is performed following ACMG and ClinGen standards (17) using the AutoCNV (53) tool (see below for details). As for sequence variants, users can modify each criterion by checking and unchecking or adapting the points given for the respective criterion (Figure 1D). Since both, InterVar and AutoCNV, can provide inaccurate pathogenicity predictions not only manual completion of unassigned ACMG criteria but also checking of prefilled ACMG criteria is crucial for the correct assessment of a variant. Therefore, we designed REEV as a diagnostic decision support system putting a focus on the semi-automated classification by the user themself. To this end, REEV assists the user with the aforementioned explanation of the definition and application of every ACMG criterion and the easy option to adapt them.

#### Automated ACMG classification by InterVar and AutoCNV

InterVar (52) implements an automated evaluation of the ACMG criteria (16) including the pathogenic PVS1, PS1, PS4, PM1, PM2, PM4, PM5, PP2, PP3, PP5 criteria and the benign BA1, BS1, BS2, BP1, BP3, BP4, BP6, BP7 criteria. InterVar uses ESP6500, 1000 Genomes and ExAC for evaluating population frequency data. Prediction scores are obtained through dbNSFP and dbSCNV, with MetaSVM used as a score for deleteriousness and GERP++ used for conservation. Splice predictions use ADA and RF scores. Pathogenicity information is determined through ClinVar variants.

AutoCNV (53) automatically classifies CNVs using the ACMG/ClinGen CNV criteria (17). AutoCNV implements automated evaluation of all criteria in Section 1 and 3, as well as most of Section 2 (excluding 2J and 2K) and criteria 4O in Section 4. DGV (42) and gnomAD (6,30) were used to obtain frequency information. Haploinsufficiency and triplosensitivity information were obtained through Decipher (28). AutoPVS1 (54) is used to evaluate the impact of gene-level duplications and deletions. All sections and criteria concerned with phenotype specificity, segregation or patient phenotyping require manual evaluation by the user.

### Additional features for logged-in users

While REEV is fully functional without login, there are a few features only available after login. Storing ACMG assessment and bookmarks on the server requires a login to be uniquely assigned to users and across computers. Users can always store the persistent URLs into the REEV server as browser bookmarks and share these URLs without registration, e.g. via Email. ClinVar (35) uploads can only be made available after login as this requires depositing the institution's ClinVar API key on the server which should be protected by login.

Logging in is possible via an ORCID account (55) or LifeScience RI (56) (which allows researchers in the European Union to use their home institution accounts).

#### Storing bookmarks and ACMG assessments

Logged-in users can store bookmarks on genes and variants on the server. In addition, they can store the phenotype information they entered for their case as well as the ACMG variant assessment scores (both the automated analysis results as well as their adjustments).

#### ClinVar upload

Logged-in users can deposit their ClinVar (35) API key and use it to submit their clinical variant assessments to ClinVar. To the best of our knowledge, REEV is the first graphical tool to allow for such uploads via the API. Submission via the API has the advantage that ClinVar submission accession identifiers can be obtained within a few hours.

We plan to add further functionality for logged-in users such as publicly sharing comments on variants.

### Benchmarking and software testing

We benchmarked REEV’s performance with regard to accuracy and speed using a defined set of 10 different variants investigated independently by six different clinicians. These ten variants comprise two null variants, two missense variants, two splice site variants, two deletions and two duplications (Supplementary Table S1). Correctness of displayed information (e.g. gnomAD frequencies, ClinVar entries, pathogenicity predictions, etc.) was checked. Classification of variants was carried out according to current ACMG and ACGS guidelines (16,17,51). Time until reaching final ACMG classification was measured and compared between using REEV and single look-up of information required for classification. Statistical analysis was performed using a one-sided paired *t*-test.

## Results and discussion

Here we present REEV, a free and open tool for the user-friendly automated clinical evaluation of genetic variants.

### Accuracy and performance in comparison to single tools

Testing REEV’s accuracy using ten different benchmarking variants showed a correct presentation of this information. When this information was used to classify the respective variant with the help of the semi-automated ACMG classification offered by REEV this classification overall matched the clinician's classification reached without using REEV. For small variants, REEV significantly reduced the time required to reach final ACMG classification (compared to single look-up of information). For structural variants we could only detect a significant reduction of the time required for classification for deletions (Supplementary Figure S3).

### Comparison to existing integrative tools

To benchmark REEV’s potential use in the clinical routine, we compared REEV and ten similar state-of-the-art web tools (Table 2). Of the ten evaluated tools gathering variant effect prediction, only five provide (detailed) information on gene-disease-associations. The only other tool enabling the annotation with case specific phenotype information and returning gene-to-phenotype ranks is the commercial platform Genoox Franklin. A frequent short-coming of existing variant interpretation platforms is their restriction to coding small variants. Apart from REEV, we found only DECIPHER and the two merely commercial services, Genoox Franklin and Varsome, supporting both small variants and structural variants. The ability to submit variants directly to the ClinVar database is only available in one other software: the commercial offering Varsome.

**Table 2.** Feature comparison of REEV with existing tools

	URL	Latest update	Documentation / Tutorial	Support of structural variants	Gene info	Gene pathogenicity	Associated conditions	Gene literature	Direct literature link-outs	Gene expression	Gene ClinVar	Case specificity / Gene-to-phenotype ranking	Variant details	Variant pathogenicity scores	Variant tool link-outs	Variant classification (according ACMG guidelines)	Integrated genome browser	Integrated protein viewer	Pharmacology information	Customization options	Option for ClinVar upload	Login required?	additional features with login?	Open access?	Open source?	Certified for clinical use?	academic or commercial?
REEV	reev.bihealth.org	2024	✓	✓	✓	✓	✓	✓	✓	✓	✓	✓	✓	✓	✓	✓	(✓)^10^	-	-	✓	✓	-	✓	✓	✓	-	academic
AutoCNV	phoenix.bgi.com/autocnv/	2021	-	✓	-	-	-	-	-	-	-	-	✓	✓	-	✓	-	-	-	✓	-	-	-	✓	✓	-	academic
DECIPHER	deciphergenomics.org	2024	✓	✓	✓	✓	✓	(✓)^5^	(✓)^5^	-	✓	-	(✓)^1, 2^	-	-	-	✓	-	-	✓		-	✓	✓	-	-	academic
Ensembl VEP Web	ensembl.org/Tools/VEP	2024	✓	-	-	-	✓	-	-	-	-	-	✓	✓	-	-	-	-	-	✓	-	-	✓	✓	✓	-	academic
GeneBe	genebe.net	2023	-	-	✓	✓	✓	(✓)^5^	(✓)^5^	-	✓	-	✓	✓	✓	✓	✓	-	-	-	-	-	✓	✓	✓	-	academic
Genoox Franklin	franklin.genoox.com	2023	✓	✓	✓	✓	✓	✓	✓	✓	(✓)^4^	✓	✓	✓	-	✓	✓	-	-	✓	-	-	✓	✓	-	-	commercial
InterVar	wintervar.wglab.org	2022	-	-	(✓)^4^	(✓)^4^	(✓)^4^	-	-	-	-	-	✓	✓	-	✓	-	-	-	✓	-	-	-	✓	-	-	academic
UCSC Variant Annotation Integrator	genome.ucsc.edu/cgi-bin/hgVai	2024	✓	-	(✓)^1^	-	(✓)^1^	-	-	(✓)^1^	-	-	✓	✓	-	-	✓	-	-	✓	-	-	✓	✓	✓	-	academic
VannoPortal	mulinlab.org/vportal	2020	✓	-	(✓)^4^	-	(✓)^8^	-	-	-	-	-	✓	✓	(✓)^6^	-	✓	-	✓	-	-	-	-	✓	-	-	academic
VarCards	genemed.tech/varcards	2019	✓	-	(✓)^4^	✓	-	-	-	-	-	-	✓	✓	(✓)^7^	-	-	-	-	-	-	-	-	✓	-	-	academic
Varsome	varsome.com	2024	✓	✓	✓	✓ ^3^	✓ ^3^	✓	✓	✓	✓	-	✓	✓	✓	✓	✓	✓	✓ ^9^	(✓)^1^	✓	(✓)^2^	✓	(✓)^1^	-	-	commercial

^1^limited functionality; ^2^limited access without login; ^3^limited access without purchase; ^4^linkout only; ^5^pubmed link-out only; ^6^ClinVar only; ^7^UCSC only; ^8^GWAS only; ^9^access only upon purchase; ^10^for structural variants only.

### Distinctive features and use cases

**Figure 2.**
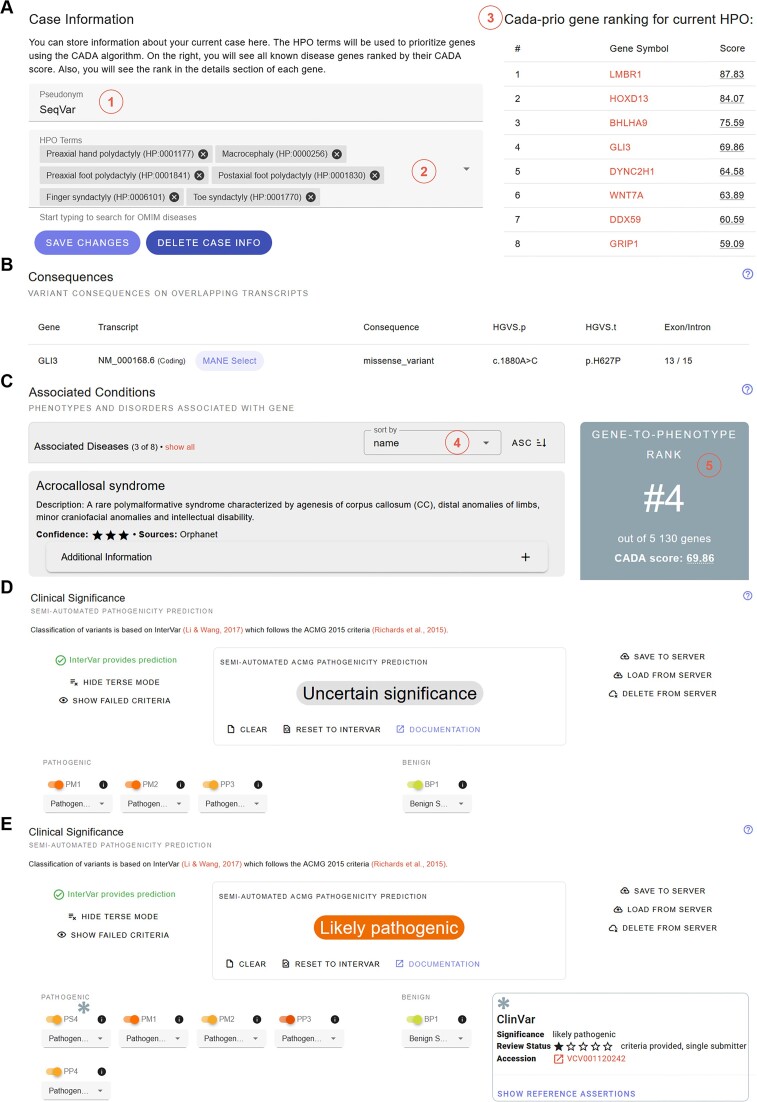
Use case: sequence variant. Example query for the sequence variant chr7:42012159:T:G. Variants can be either entered as genomic variant or on cDNA level providing the corresponding GenBank transcript variant, e.g. NM_000168.6(*GLI3*):c.1880A>C. Here, we highlight key features provided by REEV; for a full use case demonstration, e.g. including an example on the overview given to the affected gene, see the REEV quickstart and tutorial at reev.readthedocs.io. (**A**) Logged-in users can provide a case specific pseudonym/id (**1**) and phenotype information in the form of HPO terms or OMIM diseases (**2**). In our example, we provided a patient phenotype which is a preaxial hand and foot polydactyly, foot postaxial polydactyly, syndactyly and macrocephaly. REEV automatically suggests possible genes linked to the given phenotype, as well as genes, variants in which might be likely to be causative of this phenotype (as provided by CADA-prio HPO-gene-ranking) (note, that this phenotypic information does not influence automated variant predictions by InterVar and AutoCNV, but the results of these gene-to-phenotype rankings can be taken into account by the user for the manual completion of variant classification according to ACMG guidelines.) (**3**) *GLI3*, the gene affected by the likely causative variant, is listed in 4th position and possible differential diagnoses: *LMBR1*, *HOXD13*, etc. are also shown. (**B**) Overview of the variant and its consequences summarized by REEV. (**C**) Associated conditions (shown here: Orphanet; but OMIM, HPO and Genomics England PanelApp are also available in REEV) for the gene affected by the respective variant. This list can also be sorted either by name or level of confidence (**4**). Note, when logged-in users have provided case specific phenotype information (see A), here they are shown a gene-to-phenotype rank (according to CADA ranking) (**5**). (**D**) Semi-automated ACMG classification based on InterVar which classifies this variant as of uncertain significance. (**E**) Manual input of further information allows for the final classification as likely pathogenic: REEV shows that this variant is listed once in the ClinVar database as likely pathogenic (*). We thus can additionally assign the ACMG criterion PS4 on supporting level. Using REVEL as our reference pathogenicity prediction tool and this variant yielding a REVEL score of 0.96 we can use the PP3 criterion at strong level according to Pejaver *et al.* (46) (see also Figure 1A–C).

**Figure 3.**
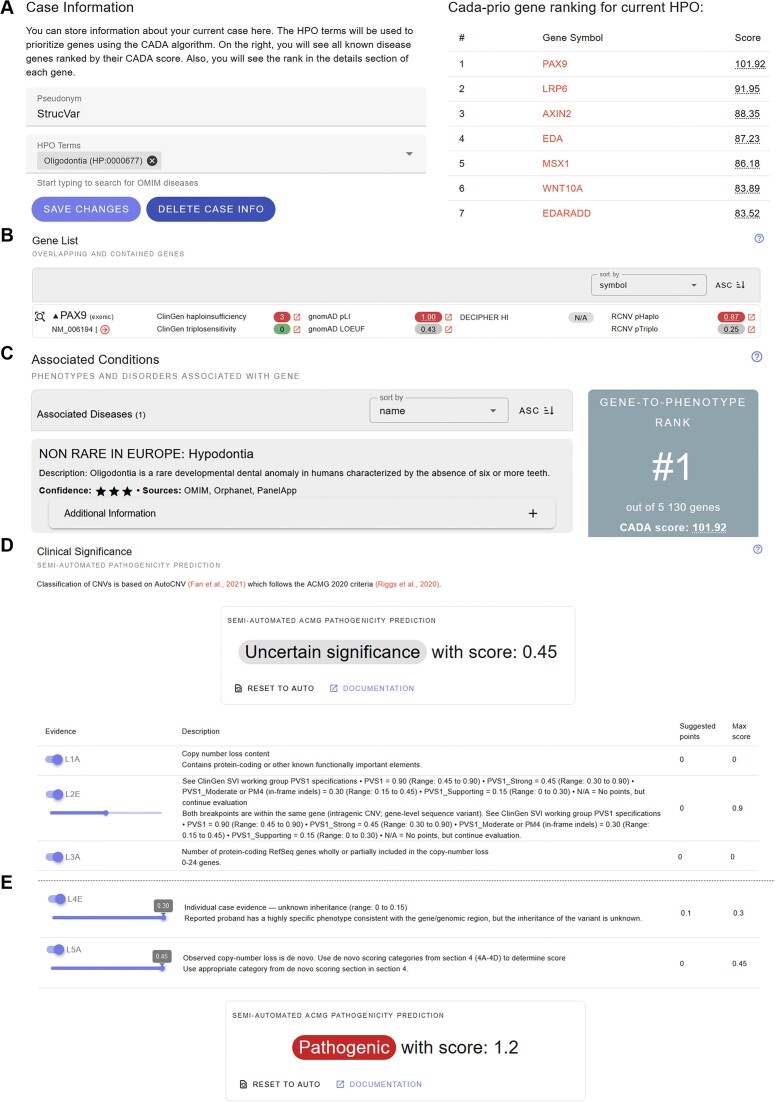
Use case: structural variant. Example query of the structural variant DEL:chr14:37131998:37133815. Again, here we demonstrate the unique features provided by REEV; for a full use case demonstration see the REEV quickstart and tutorial at reev.readthedocs.io. (**A**) Logged-in users can provide case specific phenotype information (here we provided our patient's phenotype which is oligodontia). Again, REEV suggests possible genes linked to the given phenotype (as provided by CADA-prio HPO-gene-ranking) (*PAX9*, the gene affected by our variant is listed 1st). (**B**) Full list of genes affected by our variant as well as different dosage sensitivity scores (provided by ClinGen, gnomAD, RCNV) of the respective gene. This list can also be sorted either by gene symbol or score of interest. (**C**) Associated conditions for the gene affected by the respective variant. This list can be sorted either by name or level of confidence. Again, when a logged-in user has provided case specific phenotype information (see A), they are shown a gene-to-phenotype rank (according to CADA ranking), in our example rank for a variant in *PAX9*. (**D**) Semi-automated ACMG classification of our structural variant based on AutoCNV (see also Figure 1D) yields a variant of uncertain significance with the CNV criteria L1A true (deletion contains part of a protein coding gene), L2E true with 0.45 points (corresponding to the PVS1 criterion; our variant abrogates >10% of the protein and PVS1 is to be used at strong level, equaling 0.45 points for L2E) and L3A true (for number of contained genes is 0–24). (**E**) Manual input of further information allows for the final classification as pathogenic: ClinVar information of this variant provided by REEV revealed several (likely) pathogenic loss of function sequence variants in *PAX9*, therefore criterion L4E is true with 0.3 points. Also, we identified this variant in a trio-genome sequencing as a *de novo* variant, so criterion L5A is true with 0.45 points.

With REEV we present a free and open easy-to-use, versatile web application that integrates all relevant information on genes, sequence, and structural variants into one single platform. By combining extensive information on genes and their clinical relevance (gene-disease associations, dosage sensitivity scores, etc.) as well as compiling variant specific pathogenicity predictions and database information (gnomAD v4, ClinVar, etc.) REEV sets the basis for the rapid and thorough evaluation of a variant of interest. As described above, a stand-alone feature of REEV amongst further academic variant interpretation platforms is its capability of taking case specific phenotype information and returning gene-to-phenotype ranking, helping the user to rate a variant's significance. To achieve this, we implemented semi-automated predictions for small (InterVar) and structural (AutoCNV) variants and combined them with a flexible, easy-to-use interactive system for manual interpretation and completion of ACMG criteria (Figure 1). However, REEV does not only aim to support the daily work of assessing a variant's relevance in routine NGS diagnostics but also wants to provide valuable additional information when it comes to rating a variant's possible significance in a research setting. We summarize and demonstrate these features in two use cases, one for a sequence (Figure 2) and one for a structural variant (Figure 3).

## Conclusion

REEV is a free versatile web tool that implements all the information needed for a fast and complete assessment of sequence as well as structural variants. By allowing for case specific phenotype-to-gene ranking, REEV assists the user in a fast prioritization of clinically relevant variants. With a special focus on ACMG classification and an user-friendly way of submitting the evaluated variant to the ClinVar database, REEV is useful in a clinical geneticist's daily routine. REEV also provides valuable additional information necessary when further evaluating variants in a research context.

REEV is used in the authors’ daily work and actively maintained. We welcome questions, comments, and suggestions via email or the GitHub project's issue tracker and discussion forum.

## Supplementary Material

gkae366_Supplemental_Files

## Data Availability

All data is available for download as described in the software manual attached as Supplementary Data, in particular software available on GitHub under permissive MIT license and also deposited with Zenodo (https://doi.org/10.5281/zenodo.10424214). This also includes all pipelines and software to download and preprocess the data as well as test code and deployment scripts. All data has been aggregated and generated from freely available sources. Instructions for downloading the data from our S3 server are available in the software manual as well.

## References

[1] Sun Y., Ruivenkamp C.A.L., Hoffer M.J.V., Vrijenhoek T., Kriek M., Asperen C.J., Dunnen J.T., Santen W.E. Next-generation diagnostics: gene panel, exome, or whole genome?. Hum. Mutat. 2015; 36:648–655. 25772376 10.1002/humu.22783

[2] Kliegman R.M., Bordini B.J. Undiagnosed and Rare Diseases, An Issue of Clinics in Perinatology: Undiagnosed and Rare Diseases, An Issue of Clinics in Perinatology. 2020; Elsevier Health Sciences.

[3] Turro E., Astle W.J., Megy K., Gräf S., Greene D., Shamardina O., Allen H.L., Sanchis-Juan A., Frontini M., Thys C. et al. Whole-genome sequencing of patients with rare diseases in a national health system. Nature. 2020; 583:96–102. 32581362 10.1038/s41586-020-2434-2 PMC7610553

[4] Schmidt A., Danyel M., Grundmann K., Brunet T., Klinkhammer H., Hsieh T.-C., Engels H., Peters S., Knaus A., Moosa S. et al. Next-generation phenotyping integrated in a national framework for patients with ultra-rare disorders improves genetic diagnostics and yields new molecular findings. 2023; medRxiv doi:25 April 2023, preprint: not peer reviewed 10.1101/2023.04.19.23288824. PMC11319204 39039281

[5] Lappalainen T., Scott A.J., Brandt M., Hall M. Genomic analysis in the age of human genome sequencing. Cell. 2019; 177:70–84. 30901550 10.1016/j.cell.2019.02.032 PMC6532068

[6] Chen S., Francioli L.C., Goodrich J.K., Collins R.L., Kanai M., Wang Q., Alföldi J., Watts N.A., Vittal C., Gauthier L.D. et al. A genomic mutational constraint map using variation in 76,156 human genomes. Nature. 2023; 625:92–100. 38057664 10.1038/s41586-023-06045-0 PMC11629659

[7] Smedley D., Jacobsen J.O.B., Jäger M., Köhler S., Holtgrewe M., Schubach M., Siragusa E., Zemojtel T., Buske O.J., Washington N.L. et al. Next-generation diagnostics and disease-gene discovery with the exomiser. Nat. Protoc. 2015; 10:2004–2015. 26562621 10.1038/nprot.2015.124 PMC5467691

[8] Holtgrewe M., Stolpe O., Nieminen M., Mundlos S., Knaus A., Kornak U., Seelow D., Segebrecht L., Spielmann M., Fischer-Zirnsak B. et al. VarFish: comprehensive DNA variant analysis for diagnostics and research. Nucleic Acids Res. 2020; 48:W162–W169. 32338743 10.1093/nar/gkaa241 PMC7319464

[9] Hombach D., Schuelke M., Knierim E., Ehmke N., Schwarz J.M., Fischer-Zirnsak B., Seelow D. MutationDistiller: user-driven identification of pathogenic DNA variants. Nucleic Acids Res. 2019; 47:W114–W120. 31106342 10.1093/nar/gkz330 PMC6602447

[10] Xin J., Mark A., Afrasiabi C., Tsueng G., Juchler M., Gopal N., Stupp G.S., Putman T.E., Ainscough B.J., Griffith O.L. et al. High-performance web services for querying gene and variant annotation. Genome Biol. 2016; 17:91. 27154141 10.1186/s13059-016-0953-9 PMC4858870

[11] Niroula A., Vihinen M. Variation interpretation predictors: principles, types, performance, and choice. Hum. Mutat. 2016; 37:579–597. 26987456 10.1002/humu.22987

[12] Kopanos C., Tsiolkas V., Kouris A., Chapple C.E., Aguilera M.A., Meyer R., Massouras A. VarSome: the human genomic variant search engine. Bioinformatics. 2018; 35:1978–1980. 10.1093/bioinformatics/bty897 PMC6546127 30376034

[13] Li J., Shi L., Zhang K., Zhang Y., Hu S., Zhao T., Teng H., Li X., Jiang Y., Ji L. et al. VarCards: an integrated genetic and clinical database for coding variants in the human genome. Nucleic Acids Res. 2018; 46:D1039–D1048. 29112736 10.1093/nar/gkx1039 PMC5753295

[14] Huang D., Zhou Y., Yi X., Fan X., Wang J., Yao H., Sham P.C., Hao J., Chen K., Li J. VannoPortal: multiscale functional annotation of human genetic variants for interrogating molecular mechanism of traits and diseases. Nucleic Acids Res. 2022; 50:D1408–D1416. 34570217 10.1093/nar/gkab853 PMC8728305

[15] Geoffroy V., Lamouche J.-B., Guignard T., Nicaise S., Kress A., Scheidecker S., Béchec A.L., Muller J. The AnnotSV webserver in 2023: updated visualization and ranking. Nucleic Acids Res. 2023; 51:W39–W45. 37216590 10.1093/nar/gkad426 PMC10320077

[16] Richards S., Aziz N., Bale S., Bick D., Das S., Gastier-Foster J., Grody W.W., Hegde M., Lyon E., Spector E. Karl Voelkerding, Heidi L Rehm, and ACMG Laboratory Quality Assurance Committee. Standards and guidelines for the interpretation of sequence variants: a joint consensus recommendation of the american college of medical genetics and genomics and the association for molecular pathology. Genet. Med. 2015; 17:405–424. 25741868 10.1038/gim.2015.30 PMC4544753

[17] Rooney Riggs E., Andersen E.F., Cherry A.M., Kantarci S., Kearney H., Patel A., Raca G., Ritter D.I., South S.T., Thorland E.C. et al. Technical standards for the interpretation and reporting of constitutional copy-number variants: a joint consensus recommendation of the american college of medical genetics and genomics (ACMG) and the clinical genome resource (ClinGen). Genet. Med. 2020; 22:245–257. 31690835 10.1038/s41436-019-0686-8 PMC7313390

[18] Mölder F., Jablonski K.P., Letcher B., Hall M.B., Tinch C.H.T., Sochat V., Forster J., Lee S., Twardziok S.O., Kanitz A. et al. Sustainable data analysis with snakemake. F1000Res. 2021; 10:33. 34035898 10.12688/f1000research.29032.1 PMC8114187

[19] Köster J., Rahmann S. Snakemake—a scalable bioinformatics workflow engine. Bioinformatics. 2012; 28:2520–2522. 22908215 10.1093/bioinformatics/bts480

[20] Barker M., Hong N.P.C., Katz D.S., Lamprecht A.-L., Ortiz C.M., Psomopoulos F., Harrow J., Castro L.J., Gruenpeter M., Martinez P.A. et al. Introducing the FAIR principles for research software. Sci. Data. 2022; 9:622. 36241754 10.1038/s41597-022-01710-x PMC9562067

[21] Robinson P.N., Köhler S., Bauer S., Seelow D., Horn D., Mundlos S. The human phenotype ontology: a tool for annotating and analyzing human hereditary disease. Am. J. Hum. Genet. 2008; 83:610–615. 18950739 10.1016/j.ajhg.2008.09.017 PMC2668030

[22] Köhler S., Gargano M., Matentzoglu N., Carmody L.C., Smith D.L., Vasilevsky N.A., Danis D., Balagura G., Baynam G., Brower A.M. et al. The human phenotype ontology in 2021. Nucleic Acids Res. 2021; 49:D1207–D1217. 33264411 10.1093/nar/gkaa1043 PMC7778952

[23] Gargano M.A., Matentzoglu N., Coleman B., Addo-Lartey E.B., Anagnostopoulos A.V., Anderton J., Avillach P., Bagley A.M., Bakstein E., Balhoff J.P. et al. The human phenotype ontology in 2024: phenotypes around the world. Nucleic Acids Res. 2024; 52:D1333–D1346. 37953324 10.1093/nar/gkad1005 PMC10767975

[24] Peng C., Dieck S., Schmid A., Ahmad A., Knaus A., Wenzel M., Mehnert L., Zirn B., Haack T., Ossowski S. et al. CADA: phenotype-driven gene prioritization based on a case-enriched knowledge graph. NAR Genom. Bioinform. 2021; 3:lqab078. 34514393 10.1093/nargab/lqab078 PMC8415429

[25] Dunnen J.T. Describing sequence variants using HGVS nomenclature. Methods Mol. Biol. 2017; 1492:243–251. 27822869 10.1007/978-1-4939-6442-0_17

[26] Hart R.K., Rico R., Hare E., Garcia J., Westbrook J., Fusaro A. A python package for parsing, validating, mapping and formatting sequence variants using HGVS nomenclature. Bioinformatics. 2015; 31:268–270. 25273102 10.1093/bioinformatics/btu630 PMC4287946

[27] Sayers E.W., Bolton E.E., Rodney Brister J., Canese K., Chan J., Comeau D.C., Connor R., Funk K., Kelly C., Kim S. et al. Database resources of the national center for biotechnology information. Nucleic Acids Res. 2022; 50:D20–D26. 34850941 10.1093/nar/gkab1112 PMC8728269

[28] Firth H.V., Richards S.M., Paul Bevan A., Clayton S., Corpas M., Rajan D., Vooren S.V., Moreau Y., Pettett R.M., Carter P. DECIPHER: database of chromosomal imbalance and phenotype in humans using ensembl resources. Am. J. Hum. Genet. 2009; 84:524–533. 19344873 10.1016/j.ajhg.2009.03.010 PMC2667985

[29] McKusick V.A. Mendelian inheritance in man and its online version, OMIM. Am. J. Hum. Genet. 2007; 80:588–604. 17357067 10.1086/514346 PMC1852721

[30] Karczewski K.J., Francioli L.C., Tiao G., Cummings B.B., Alföldi J., Wang Q., Collins R.L., Laricchia K.M., Ganna A., Birnbaum D.P. et al. The mutational constraint spectrum quantified from variation in 141,456 humans. Nature. 2020; 581:434–443. 32461654 10.1038/s41586-020-2308-7 PMC7334197

[31] Rehm H.L., Berg J.S., Brooks L.D., Bustamante C.D., Evans J.P., Landrum M.J., Ledbetter D.H., Maglott D.R., Martin C.L., Nussbaum R.L. et al. ClinGen. ClinGen–the clinical genome resource. N. Engl. J. Med. 2015; 372:2235–2242. 26014595 10.1056/NEJMsr1406261 PMC4474187

[32] Rath A., Olry A., Dhombres F., Brandt M.M., Urbero B., Ayme S. Representation of rare diseases in health information systems: the orphanet approach to serve a wide range of end users. Hum. Mutat. 2012; 33:803–808. 22422702 10.1002/humu.22078

[33] Martin A.R., Williams E., Foulger R.E., Leigh S., Daugherty L.C., Niblock O., Leong I.U.S., Smith K.R., Gerasimenko O., Haraldsdottir E. et al. PanelApp crowdsources expert knowledge to establish consensus diagnostic gene panels. Nat. Genet. 2019; 51:1560–1565. 31676867 10.1038/s41588-019-0528-2

[34] Consortium G.T.E. The GTEx consortium atlas of genetic regulatory effects across human tissues. Science. 2020; 369:1318–1330. 32913098 10.1126/science.aaz1776 PMC7737656

[35] Landrum M.J., Chitipiralla S., Brown G.R., Chen C., Gu B., Hart J., Hoffman D., Jang W., Kaur K., Liu C. et al. ClinVar: improvements to accessing data. Nucleic Acids Res. 2020; 48:D835–D844. 31777943 10.1093/nar/gkz972 PMC6943040

[36] Wei C.-H., Allot A., Lai P.-T., Leaman R., Tian S., Luo L., Jin Q., Wang Z., Chen Q., Lu Z. PubTator 3.0: an AI-powered literature resource for unlocking biomedical knowledge. Nucleic Acids Res. 2024; 10.1093/nar/gkae235. PMC11223843 38572754

[37] Wei C.-H., Allot A., Leaman R., Lu Z. PubTator central: automated concept annotation for biomedical full text articles. Nucleic Acids Res. 2019; 47:W587–W593. 31114887 10.1093/nar/gkz389 PMC6602571

[38] Benson D.A., Cavanaugh M., Clark K., Karsch-Mizrachi I., Lipman D.J., Ostell J., Sayers E.W. GenBank. Nucleic Acids Res. 2013; 41:D36–D42. 23193287 10.1093/nar/gks1195 PMC3531190

[39] Miller W., Rosenbloom K., Hardison R.C., Hou M., Taylor J., Raney B., Burhans R., King D.C., Baertsch R., Blankenberg D. et al. 28-way vertebrate alignment and conservation track in the UCSC genome browser. Genome Res. 2007; 17:1797–1808. 17984227 10.1101/gr.6761107 PMC2099589

[40] Howe K.L., Achuthan P., Allen J., Allen J., Alvarez-Jarreta J., Amode M.R., Armean I.M., Azov A.G., Bennett R., Bhai J. et al. Ensembl 2021. Nucleic Acids Res. 2021; 49:D884–D891. 33137190 10.1093/nar/gkaa942 PMC7778975

[41] James Kent W., Sugnet C.W., Furey T.S., Roskin K.M., Pringle T.H., Zahler A.M., Haussler D. The human genome browser at UCSC. Genome Res. June 2002; 12:996–1006. 12045153 10.1101/gr.229102 PMC186604

[42] MacDonald J.R., Ziman R., Yuen R.K.C., Feuk L., Scherer W. The database of genomic variants: a curated collection of structural variation in the human genome. Nucleic Acids Res. 2014; 42:D986–D992. 24174537 10.1093/nar/gkt958 PMC3965079

[43] Schwarz J.M., Rödelsperger C., Schuelke M., Seelow D. MutationTaster evaluates disease-causing potential of sequence alterations. Nat. Methods. 2010; 7:575–576. 20676075 10.1038/nmeth0810-575

[44] Steinhaus R., Proft S., Schuelke M., Cooper D.N., Schwarz J.M., Seelow D. MutationTaster2021. Nucleic Acids Res. 2021; 49:W446–W451. 33893808 10.1093/nar/gkab266 PMC8262698

[45] Liu X., Li C., Mou C., Dong Y., Tu Y. dbNSFP v4: a comprehensive database of transcript-specific functional predictions and annotations for human nonsynonymous and splice-site SNVs. Genome Med. 2020; 12:103. 33261662 10.1186/s13073-020-00803-9 PMC7709417

[46] Pejaver V., Byrne A.B., Feng B.-J., Pagel K.A., Mooney S.D., Karchin R., O’Donnell-Luria A., Harrison S.M., Tavtigian S.V., Greenblatt M.S. et al. and ClinGen Sequence Variant Interpretation Working Group. Calibration of computational tools for missense variant pathogenicity classification and ClinGen recommendations for PP3/BP4 criteria. Am. J. Hum. Genet. 2022; 109:2163–2177. 36413997 10.1016/j.ajhg.2022.10.013 PMC9748256

[47] Rambla J., Baudis M., Ariosa R., Beck T., Fromont L.A., Navarro A., Paloots R., Rueda M., Saunders G., Singh B. et al. Beacon v2 and beacon networks: a “lingua franca” for federated data discovery in biomedical genomics, and beyond. Hum. Mutat. 2022; 43:791–799. 35297548 10.1002/humu.24369 PMC9322265

[48] Freeman P.J., Hart R.K., Gretton L.J., Brookes A.J., Dalgleish R. VariantValidator: accurate validation, mapping, and formatting of sequence variation descriptions. Hum. Mutat. 2018; 39:61–68. 28967166 10.1002/humu.23348 PMC5765404

[49] International Standing Committee on Human Cytogenomic Nomenclature ISCN 2020: An International System for Human Cytogenomic Nomenclature. 2020; Karger 2020.

[50] Robinson J.T., Thorvaldsdottir H., Turner D., Mesirov J.P. igv.js: an embeddable JavaScript implementation of the integrative genomics viewer (IGV). Bioinformatics. 2023; 39:btac830. 36562559 10.1093/bioinformatics/btac830 PMC9825295

[51] Tavtigian S.V., Harrison S.M., Boucher K.M., Biesecker G. Fitting a naturally scaled point system to the ACMG/AMP variant classification guidelines. Hum. Mutat. 2020; 41:1734–1737. 32720330 10.1002/humu.24088 PMC8011844

[52] Li Q., Wang K. InterVar: clinical interpretation of genetic variants by the 2015 ACMG-AMP guidelines. Am. J. Hum. Genet. 2017; 100:267–280. 28132688 10.1016/j.ajhg.2017.01.004 PMC5294755

[53] Fan C., Wang Z., Sun Y., Sun J., Liu X., Kang L., Xu Y., Yang M., Dai W., Song L. et al. AutoCNV: a semiautomatic CNV interpretation system based on the 2019 ACMG/ClinGen technical standards for CNVs. Bmc Genomics [Electronic Resource]. 2021; 22:721. 34615484 10.1186/s12864-021-08011-4 PMC8496072

[54] Xiang J., Peng J., Baxter S., Peng Z. AutoPVS1: an automatic classification tool for PVS1 interpretation of null variants. Hum. Mutat. 2020; 41:1488–1498. 32442321 10.1002/humu.24051

[55] Haak L.L., Fenner M., Paglione L., Pentz E., Ratner H. ORCID: a system to uniquely identify researchers. Learn. Publ. 2012; 25:259–264.

[56] Crosswell L.C., Thornton J.M. ELIXIR: a distributed infrastructure for european biological data. Trends Biotechnol. 2012; 30:241–242. 22417641 10.1016/j.tibtech.2012.02.002

[57] Rentzsch P., Witten D., Cooper G.M., Shendure J., Kircher M. CADD: predicting the deleteriousness of variants throughout the human genome. Nucleic Acids Res. 2019; 47:D886–D894. 30371827 10.1093/nar/gky1016 PMC6323892

[58] Jaganathan K., Panagiotopoulou S.K., McRae J.F., Darbandi S.F., Knowles D., Li Y.I., Kosmicki J.A., Arbelaez J., Cui W., Schwartz G.B. et al. Predicting splicing from primary sequence with deep learning. Cell. 2019; 176:535–548. 30661751 10.1016/j.cell.2018.12.015

[59] Ioannidis N.M., Rothstein J.H., Pejaver V., Middha S., McDonnell S.K., Baheti S., Musolf A., Li Q., Holzinger E., Karyadi D. et al. REVEL: an ensemble method for predicting the pathogenicity of rare missense variants. Am. J. Hum. Genet. 2016; 99:877–885. 27666373 10.1016/j.ajhg.2016.08.016 PMC5065685

[60] Vasilevsky N., Essaid S., Matentzoglu N., Harris N.L., Haendel M., Robinson P., Mungall J. Mondo disease ontology: harmonizing disease concepts across the world. CEUR Workshop Proceedings, CEUR-WS. 2020; 2807:

