# Supplementary material for: REEV: review, evaluate and explain variants
Source: Nucleic Acids Res. 2024 May 20;52(W1):W148–58. doi: 10.1093/nar/gkae366 (PMC11223839; doi:10.1093/nar/gkae366)
Supplement: gkae366_Supplemental_Files [file gkae366_supplemental_files.zip › REEV_Manual.pdf]

REEV

REEV Authors

Feb 08, 2024

USER'S MANUAL

1 Features 3

2 Open Source and Data 5

Python Module Index 69

Index 71

REEV (REEV: Explanation and Evaluation of Variants) is a web-based tool for clinicians and researchers in rare disease genetics. See below for a list of features. A good place to start learning about REEV is the *quickstart* or *tutorial*.

CHAPTER

ONE

FEATURES

It provides the following features as a central resource:

- **gene-related information and functionality:**
  - basic information, including gene summaries
  - gene pathogenicity information, such as pLI and LOEUF constraints, DECIPHER Haploinsufficiency, …
  - condition/disease-related information
  - aggregated variant information from ClinVar
  - gene-phenotype similarity information and ranking
- **sequence variant-related functionality:**
  - consequences on transcripts and proteins sequences
  - ClinVar variant information
  - gnomAD population frequency
  - UCSC 100 vertebrate conservation
  - semi-automated ACMG classification from InterVar[1]
  - link-out to external resources and tools
  - query the GA4GH beacon network for the variant
  - query the VariantValidator API for the variant
- **structural variant (currently copy number loss/gain only)-related functionality:**
  - consequences on overlapping genes
  - information on overlapping structural variants in ClinVar
  - semi-automated ACMG classification from AutoCNV[2]
  - link-out to external resources and tools
  - integrated genome browser with useful tracks for interpreting the variant

[1] Q. Li and K. Wang. InterVar: Clinical Interpretation of Genetic Variants by the 2015 ACMG-AMP Guidelines. *Am J Hum Genet*, 100(2):267–280, Feb 2017.

[2] C. Fan, Z. Wang, Y. Sun, J. Sun, X. Liu, L. Kang, Y. Xu, M. Yang, W. Dai, L. Song, X. Wei, J. Xiang, H. Huang, M. Zhou, F. Zeng, L. Huang, Z. Xu, and Z. Peng. AutoCNV: a semiautomatic CNV interpretation system based on the 2019 ACMG/ClinGen Technical Standards for CNVs. *BMC Genomics*, 22(1):721, Oct 2021.

CHAPTER TWO

OPEN SOURCE AND DATA

REEV is open source software using open data. See section *Operator Introduction* for more information.

2.1 Quickstart

Do you like to jump heads-first into the water? Then this is the place for you! For a more in-depth explanation of REEV, see the *tutorial*. However, REEV also contains helpful help links throughout the web interface so you can also look up information as you go.

2.1.1 Introduction

We have developed REEV (Review, Evaluate and Explain Variants) to help clinicians and researchers in rare disease genetics with an open and free platform to explore comprehensive information about genes as well as sequence and structural variants. REEV is designed to help you to quickly yet comprehensively record all the important information on a variant and, on the basis of this, to evaluate it on the basis of the ACMG criteria[1]. To this end, REEV also integrates methods that allow users to (semi-)automatically fill the individual ACMG criteria sections to facilitate interpretation.

Here, we provide a quick start as well as a full tutorial guiding you through the process on reviewing and evaluating genes and variants with the help of REEV.

2.1.2 Home Page

First, go to the REEV home page at https://reev.bihealth.org and you will be the following screen.

The most important part is the search bar (1). Here, you can enter your query, which can be a gene symbol or the description of a sequence or structural variant. The example box (2) has a couple of examples to get you started and so you can learn some examples. You can look at the section *Supported Queries* to learn about the supported queries.

Sequence vs. Structural Variants

We distinguish between *sequence* and *structural* variants. Sequence variants are those where the actual sequence is of importance. This is contrast to structural variants that are generally bigger and where the change of structure in the genome is more important. One could also make a distinction between small variants (say up to 50bp in size) and structural variants. Then, single nucleotide variants (SNVs) and small insertions/deletions (indels) would be sequence variants.

[1] S. Richards, N. Aziz, S. Bale, D. Bick, S. Das, J. Gastier-Foster, W. W. Grody, M. Hegde, E. Lyon, E. Spector, K. Voelkerding, and H. L. Rehm. Standards and guidelines for the interpretation of sequence variants: a joint consensus recommendation of the American College of Medical Genetics and Genomics and the Association for Molecular Pathology. *Genet Med*, 17(5):405–424, May 2015.

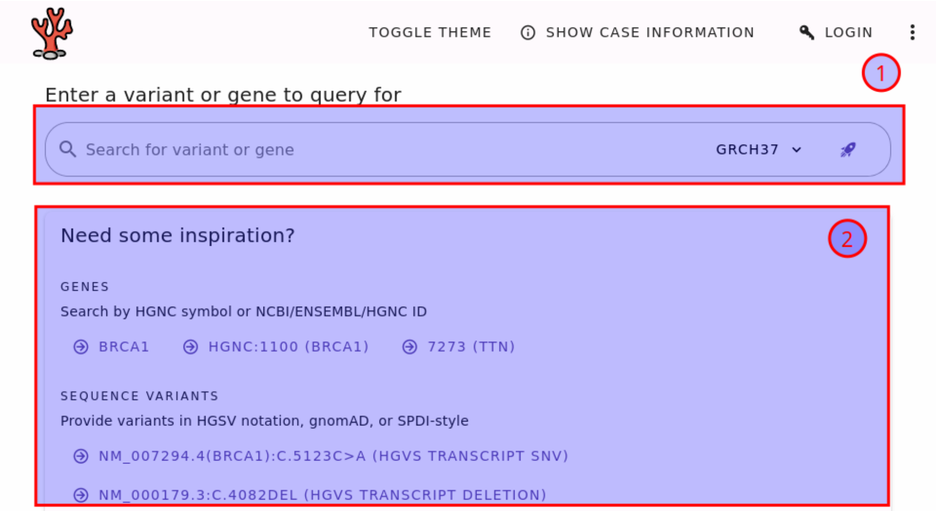

Fig. 1: The REEV home page.

However, in REEV the distinction is based on the query format. For sequence variants, variants are eventually represented by the changed chromosomal sequence. For structural variants, variants are represented by the genomic coordinates of the change. Read on, you will see what you mean in the examples.

2.1.3 Looking at Genes

Let us get started by looking at a gene. Enter `FBN1` into the search bar and press enter or click the search button. You will be redirected to the gene details page for *FBN1*.

Here, you can find the following information.

1. The basic gene information, including a short summary from NCBI Entrez.
2. Fold-out button for more details (here shown already expanded).
3. The gene fold-out pane displays information such as alternate identifiers, links to locus-specific databases, and NCBI references into functions.
4. Information about potential pathogenicity of the gene through haploinsufficiency or triplosensitivity.
5. Information about associated conditions in terms of phenotypes (HPO terms) and diseases (OMIM or Orphanet).
6. Fold-out button for more details on the associated conditions.
7. Gene expression information from the GTEx project.
8. Aggregated variant count from ClinVar regarding variant type and clinical significance assessments.
9. A plot that displays the population frequency of variants assessed as benign/uncertain/pathogenic in ClinVar.
10. A plot of the ClinVar variant assessments throughout the gene.

Note that you can use the little (?) links to get more information about the displayed information. These links will bring you to this documentation in a new browser tab/window.

Also note that you can copy and paste the URL to the gene details page and send it to your colleagues or create a bookmark of it.

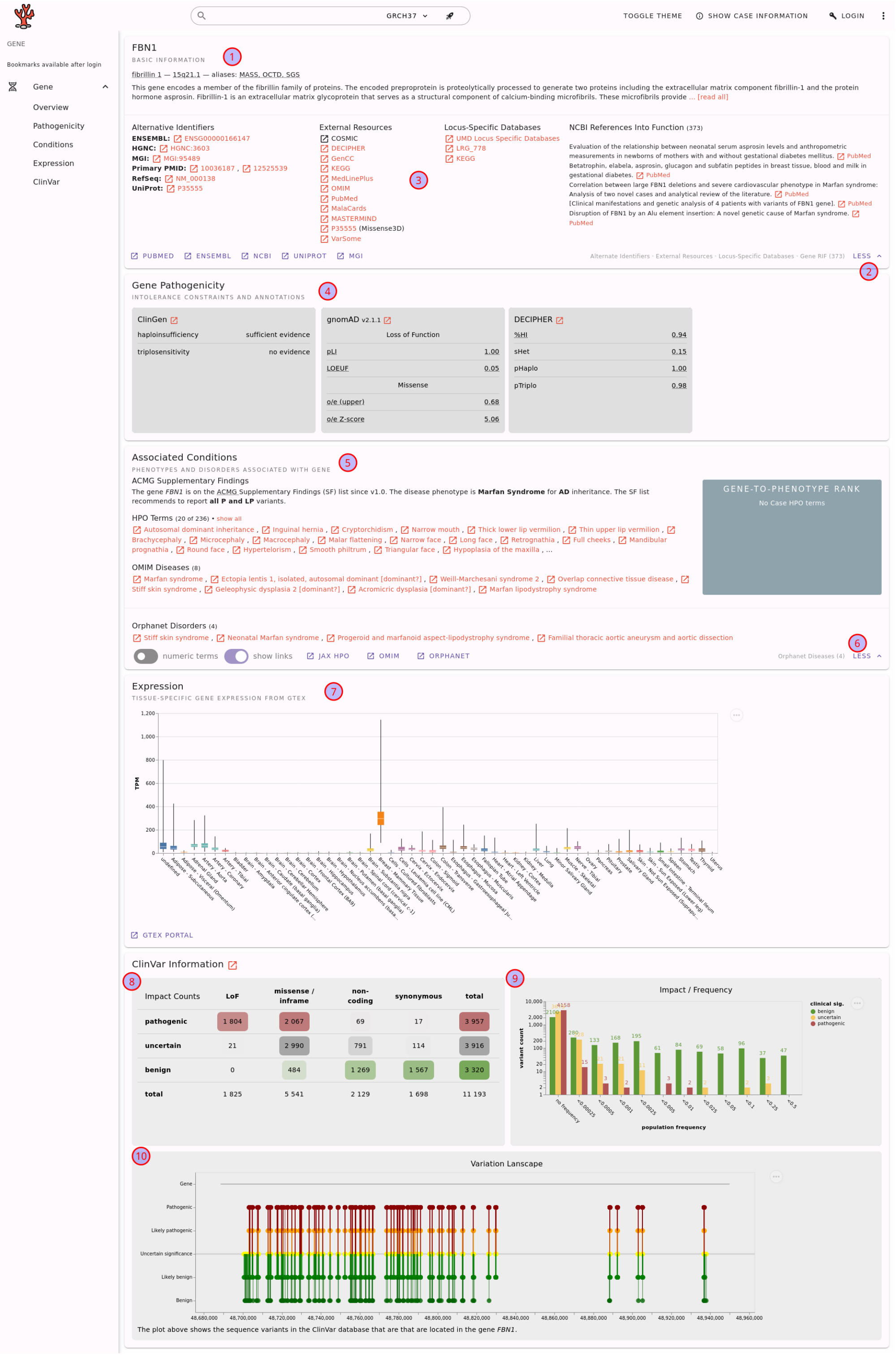

Fig. 2: The gene details page for *FBN1*.

2.1.4 Looking at Sequence Variants

Next, let us look at a SNV, which is referred to a *sequence variant* in REEV. If you want to go back to the home page, you can also reach there by clicking the little coral logo on the top left of the page. However, you can also enter the variant in the search bar at the top of the page.

You can enter variants in a number of formats, including HGVS format. See the section *Supported Queries* for a list of supported formats. We will now look at the variant `NM_000052.5(ATP7A):c.1172G>C`. Copy this variant into the search bar and press enter. You will be redirected to the variant details page for this variant.

Sharing URLs

First of all, note that you are redirected to the following URL.

- https://reev.bihealth.org/seqvar/grch37-X-77245290-G-C?orig=NM_000052.5(ATP7A):c.1172G%3EC

This URL is stable and you can send it to your colleagues to share a variant. You can find out more about the URLs in the section *Front-End URLs*.

Variant Details

On the top of the variant details page you will see the information about the gene which is identical to what is described in the section *Looking at Genes*. We will thus focus on the second half of the page shown in the following figure.

You can find the following elements on the page:

1. Semi-automated ACMG variant class assessment based on the InterVar[2] tool.
2. A table with the impact of the variant on different transcripts.
3. Information with ClinVar assertions on the variant. This displays the ClinVar reference assertion with the most pathogenic significance and its review status.
4. To obtain more information, fold out the card and look at the individual reference assertion. Each ClinVar reference assertion aggregates the submissions of a variant for a specific condition.
5. The population frequencies in dfiferent populations. Click on the population name to obtain frequency details by XX/XY karyotype.
6. Variant pathogenicity scores from different tools, aggregated by dbNSFP. To help interpreting the variant scores, we show scores calibrated following Pejaver *et al.*[3] where applicable (currently for all scores except for SpliceAI).
7. Also, you can find the UCSC 100 vertebrate conservation here on the protein level.
8. Link-outs to genome browsers and various external tools to help you to assess the variant further.
9. Query the GA4GH Beacon network for presence of the variant at other sites.
10. Submit the variant to VariantValidator to obtain gold standard HGVS representation. This is useful for a “second opinion” on the variant representation before using the variant description in a journal submission or report.

For more details, see the section *Sequence Variants* of the *Tutorial* or use the little (?) help icons on the page.

[2] Q. Li and K. Wang. InterVar: Clinical Interpretation of Genetic Variants by the 2015 ACMG-AMP Guidelines. *Am J Hum Genet*, 100(2):267–280, Feb 2017.

[3] V. Pejaver, A. B. Byrne, B. J. Feng, K. A. Pagel, S. D. Mooney, R. Karchin, A. O’Donnell-Luria, S. M. Harrison, S. V. Tavtigian, M. S. Greenblatt, L. G. Biesecker, P. Radivojac, S. E. Brenner, L. G. Biesecker, S. M. Harrison, A. A. Tayoun, J. S. Berg, S. E. Brenner, G. R. Cutting, S. Ellard, M. S. Greenblatt, P. Kang, I. Karbassi, R. Karchin, J. Mester, A. O’Donnell-Luria, T. Pesaran, S. E. Plon, H. L. Rehm, N. T. Strande, S. V. Tavtigian, and S. Topper. Calibration of computational tools for missense variant pathogenicity classification and ClinGen recommendations for PP3/BP4 criteria. *Am J Hum Genet*, 109(12):2163–2177, Dec 2022.

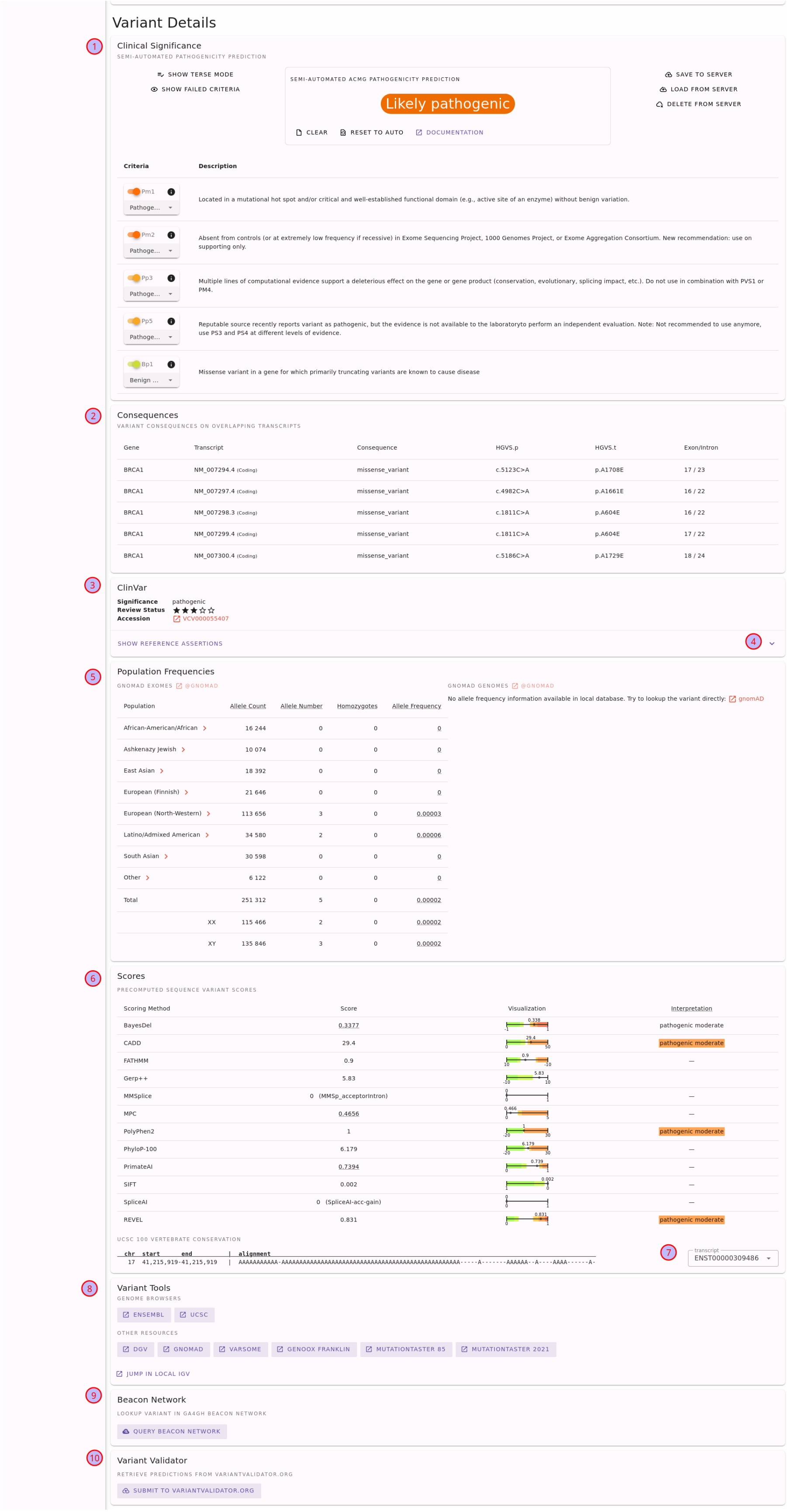

Fig. 3: Sequence variant details page for the variant `NM_000052.5(ATP7A):c.1172G>C`.

2.1.5 Looking at Structural Variants

As the last step of the quickstart, let us look at a ~100kb deletion on chromosome 17 from base pairs 41,176,312 to 41,277,500 (GRCh37 coordinates). In ISCN array notation, this is `GRCh37 17q21(41176312_41277500)x1`. For example, you can enter the variant using the ISCN notation from above or in a color-formated as `DEL:17:41176312:41277500`. You can find full list of query formats in section *Supported Queries*. Currently, REEV only supports copy number variants: deletions and duplications.

You will be redirected to the following URL. Also note that the URL to the structural variant details page is stable and can be shared with colleagues.

- https://reev.bihealth.org/strucvar/DEL-grch37-17-41176312-41277500?orig=GRCh37+17q21+(41176312_41277500)x1

At the top of the page, you will see information also shown int he following figure.

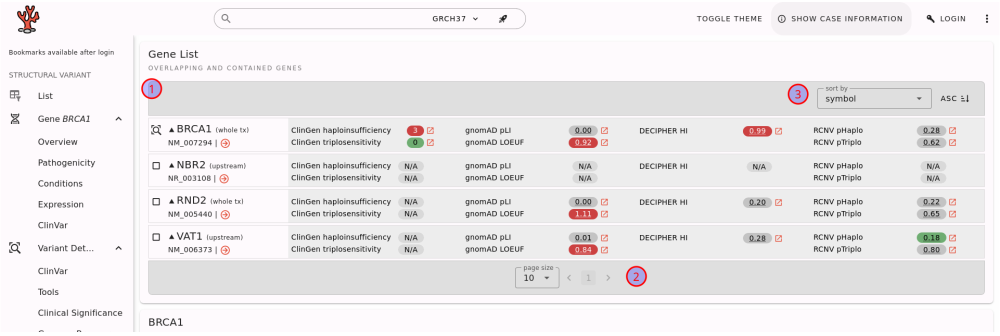

Fig. 4: Top of structural variant details page for the variant `DEL:17:41176312:41277500`.

1. You will see the list of genes that are overlapping with or close to the structural variant. Next to the symbol, you see the how the variant is affected. For example, *BRCA1* and *RND2* are fully contained while the variant is upstream of both *NBR2* and *VAT1*. You can click on the gene symbol to display the gene details on this page. You can also use the little right-pointing arrow in the circle to go to the gene details page.
2. When there are many genes then you can browse through them with the page control.
3. In the case of many overlapping genes, you probably want to use a different sort order than by gene symbol. You can use the “sort by” control to pick out the scores to sort by, e.g., gnomAD pLI score or the ClinGen haploinsufficiency or triplosensitivity assessment.

The details for the currently selected genes will be displayed below the gene table. All of this has been explained in the section *Looking at Genes* already so we will not repeat this here.

The second half of the page focuses on the variant rather than the overlapping genes. This is shown in the following figure.

4. Details on overlapping variants in ClinVar. You can also unfold each row to display the individual reference ClinVar assertions.
5. The variants will be sorted by reciprocal overlap (the fraction of the overlap of the variant - yours and the ClinVar one - and the large of the variant lenghts). This is useful to find the “best fitting” one.
6. Open the location of the variant in an external genome browser or an external tool for further analysis.
7. Semi-automated assessment of the variant following ACMG standards[Page 5, 1] using the AutoCNV[4] tool.
8. See the location of the variant in an internal genome browser with useful tracks for interpreting the variant.

[4] C. Fan, Z. Wang, Y. Sun, J. Sun, X. Liu, L. Kang, Y. Xu, M. Yang, W. Dai, L. Song, X. Wei, J. Xiang, H. Huang, M. Zhou, F. Zeng, L. Huang, Z. Xu, and Z. Peng. AutoCNV: a semiautomatic CNV interpretation system based on the 2019 ACMG/ClinGen Technical Standards for CNVs. *BMC Genomics*, 22(1):721, Oct 2021.

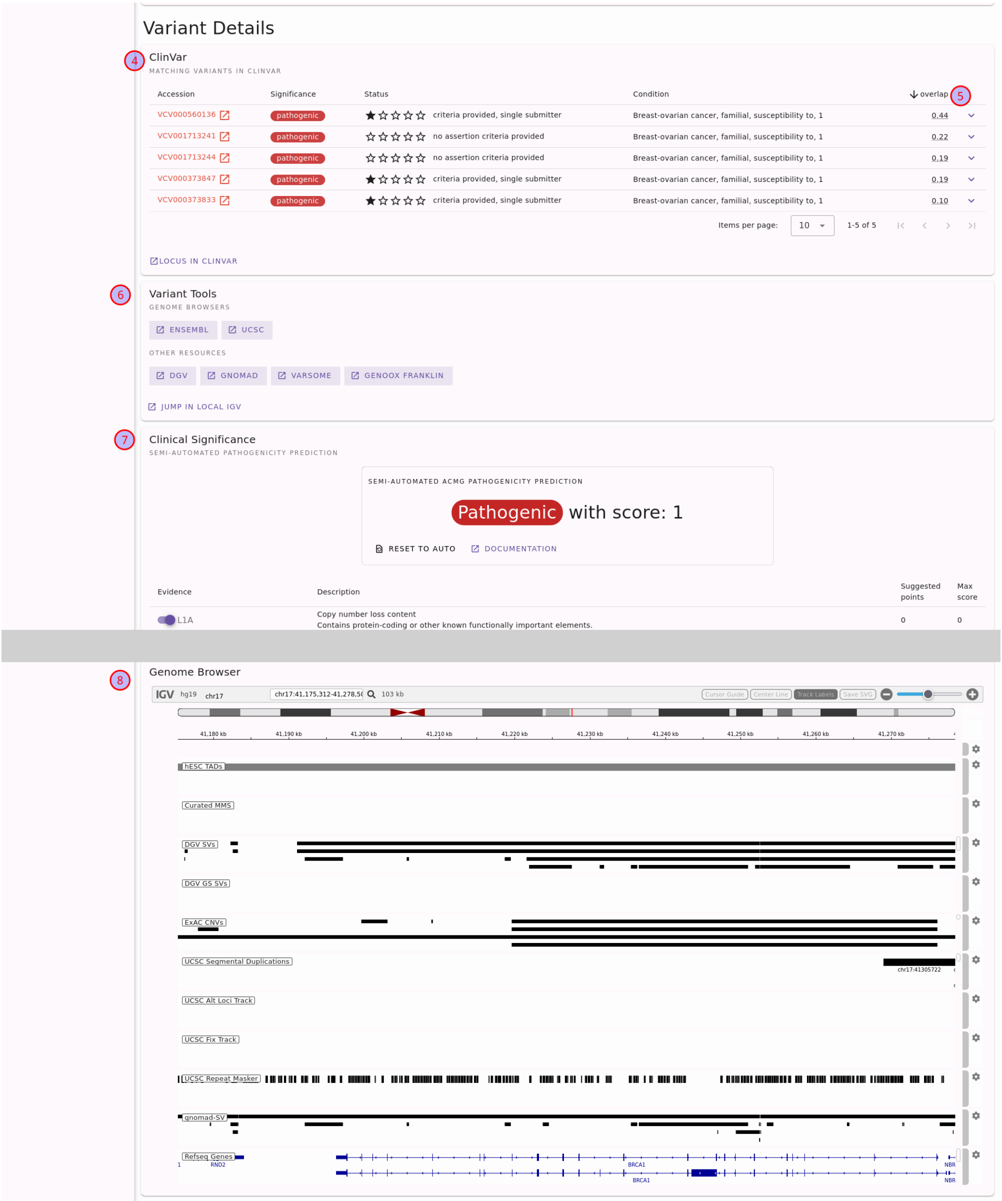

Fig. 5: Second half of structural variant details page for the variant `DEL:17:41176312:41277500`. Note that a large part of the ACMG assessment card has been cut out, indicated by the gray area.

For more details, see the section *Structural Variants* of the *Tutorial* or use the little (?) help icons on the page.

2.2 Tutorial

In this tutorial, we will walk through the analysis of a sequence and a structural variant and how to use REEV to interpret it step by step. If you want to get a brief overview of REEV, have a look at the *quickstart* section.

2.2.1 Sequence Variants

We came across the following interesting missense variant in *GLI3* in a patient with synpolydactyly which we want to explore in more detail.

Genomic variant [hg19/GRCh37]	Gene	RefSeq transcript	Variant effect
chr7:42012159:T:G	GLI3	NM_000168.6	c.1880A>C, p.(His627Pro)

Lets go the REEV start page and enter our variant here:

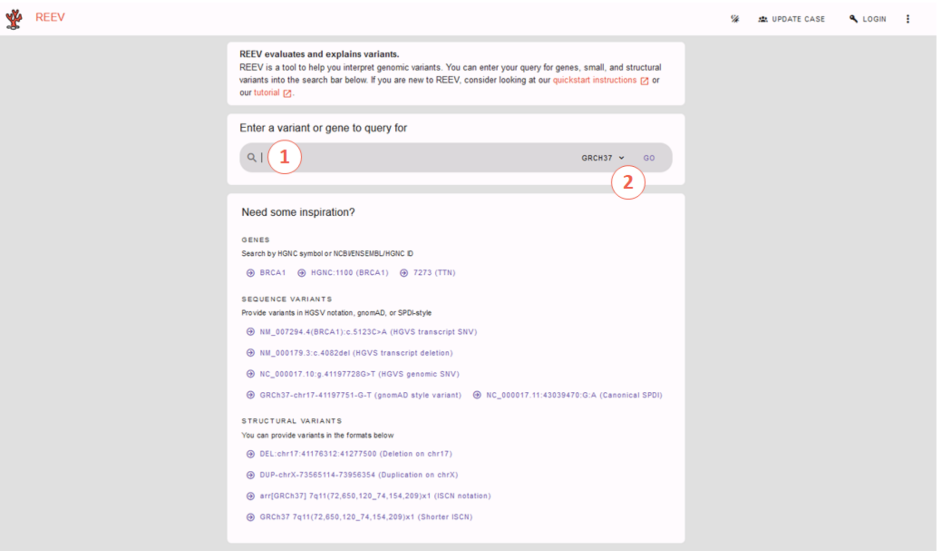

Fig. 6: The REEV home page.

To do so we have two options of entering this variant:

a) Genomic Variant

We could go for the genomic variant and enter here:

```
chr7:42012159:T:G
```

Note: Pay attention to choosing the correct reference genome in the selection box to the right.

b) Variant on cDNA Level

Go for the variant on cDNA level providing the respective RefSeq transcript variant and gene:

```
NM_000168.6(GLI3):c.1880A>C
```

which then brings us to REEV's gene and variant information. We will go through this step-by-step but if you want to jump to a specific information you can also use the overview and links on the left:

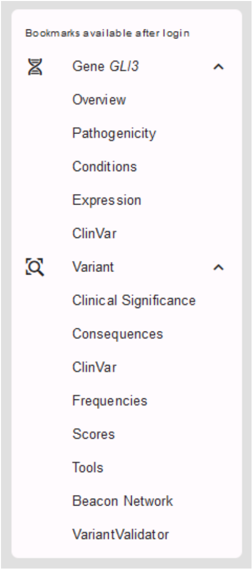

Let's start with a look at the gene information provided in the first box:

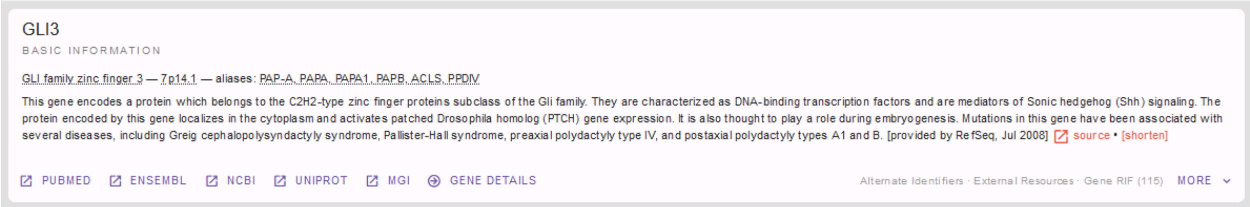

This information about the *GLI3* gene already hints at a possible link to our patient's phenotype.

Useful information on gene-phenotype-associations can also be found via the linkouts below, e.g. to *MGI*. Further useful links and information on the gene can be found when clicking on the *"more"* button in lower right corner:

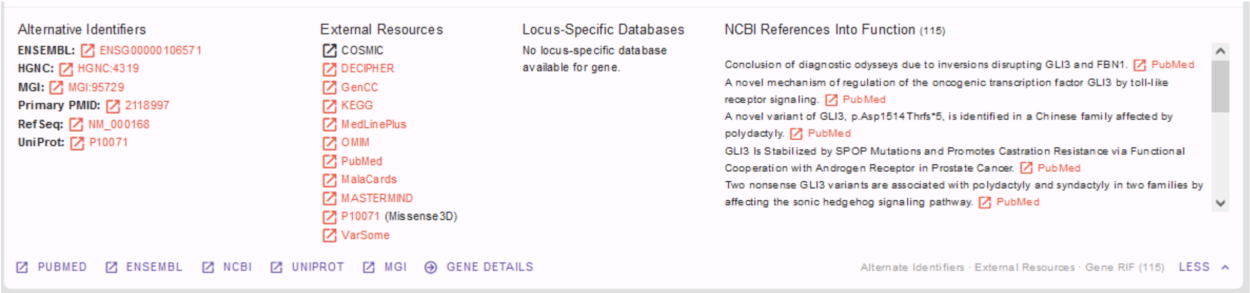

For our case, we find *GLI3* an interesting gene and move on to find out more on potential gene pathogenicity from the box below:

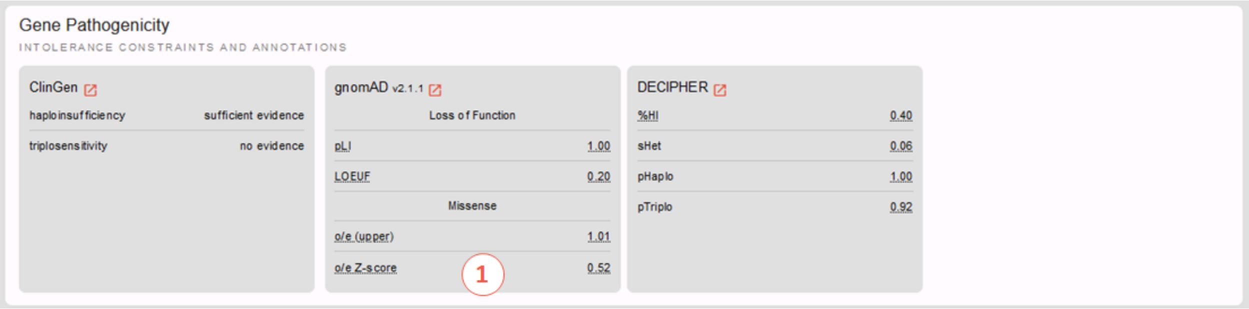

Since we are examining the missense variant we are especially interested in the gnomAD Z-score provided here (1). Noting a Z-score of 0.52 we see that *GLI3* is not missense constrained meaning that *GLI3* is tolerant for missense variations whereas with a pLI of 1.00 and a LOEUF of 0.20 gnomAD data as well as the displayed ClinGen and DECIPHER data all state haploinsufficiency meaning that *GLI3* is intolerant for heterozygous loss of function variants. We conclude that we have to look further into the pathogenicity of our specific missense variant on the variant level, if we consider *GLI3* to be a candidate gene for our patient's phenotype. We already suspect that this might be the case from the information above and know find the respective evidence in the box below:

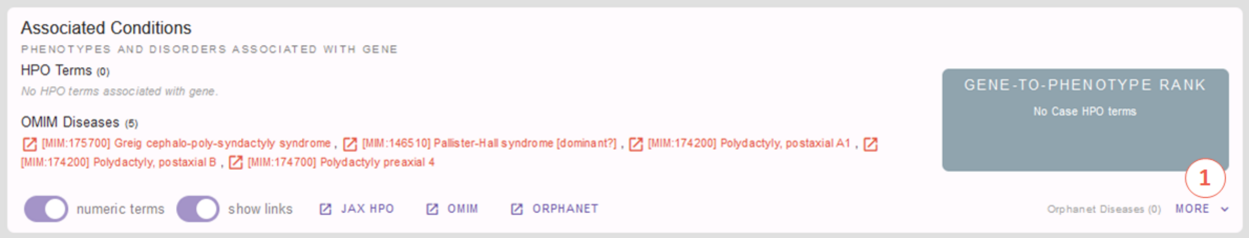

Yes! We find several known OMIM phenotypes for *GLI3* fitting our patient's phenotype. If we have logged in and provided our patient's phenotype e.g. *HP:0001177 - Preaxial hand polydactyly; HP:0001159 - Syndactyly* we will also find a score of how well our case's and the gene's phenotypes match each other. Apart from the OMIM phenotypes we also find similar links to Orphanet - and again we will find more information when clicking on "more" in the lower right corner (1).

If we had not found an established gene-phenotype-association in the section before, the following one would be of particular interest for us: Is our gene expressed in a tissue fitting our patient's affections? Let's look at the tissue specific gene expression from GTeX provided here:

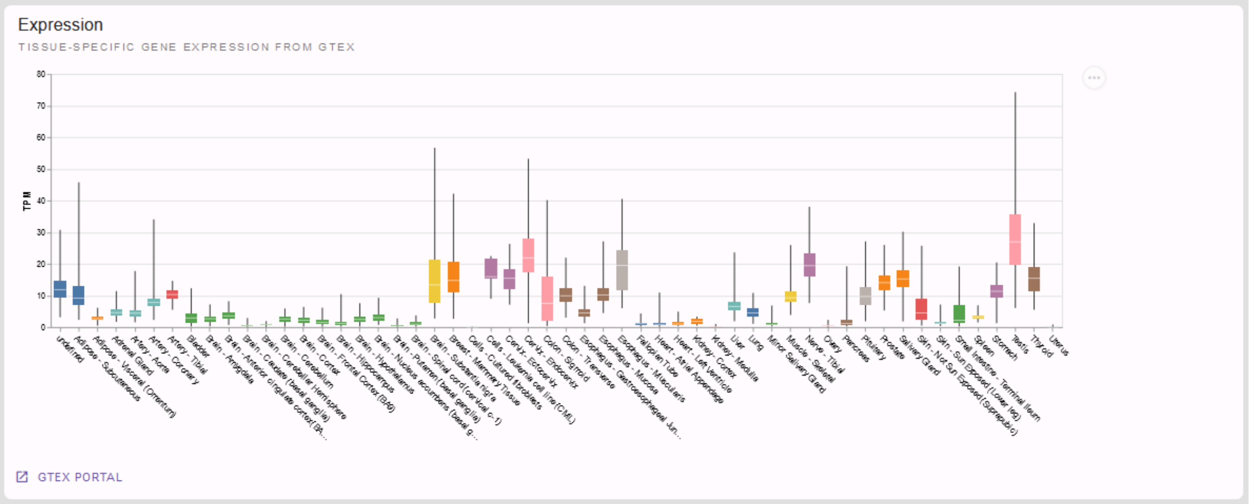

But we want to come back to our variant at hand `NM_000168.6(GLI3):c.1880A>C p.(His627Pro)`. We now know that a *GLI3* variant causing a heterozygous loss of function would cause a disease fitting our patient's phenotype. But is our variant a (likely) pathogenic one?

Before we finally look into the variant itself let's have a last look on the gene level: are there pathogenic missense variants in *GLI3* at all, e.g. in the ClinVar database? If so, in which part of the gene are they located?

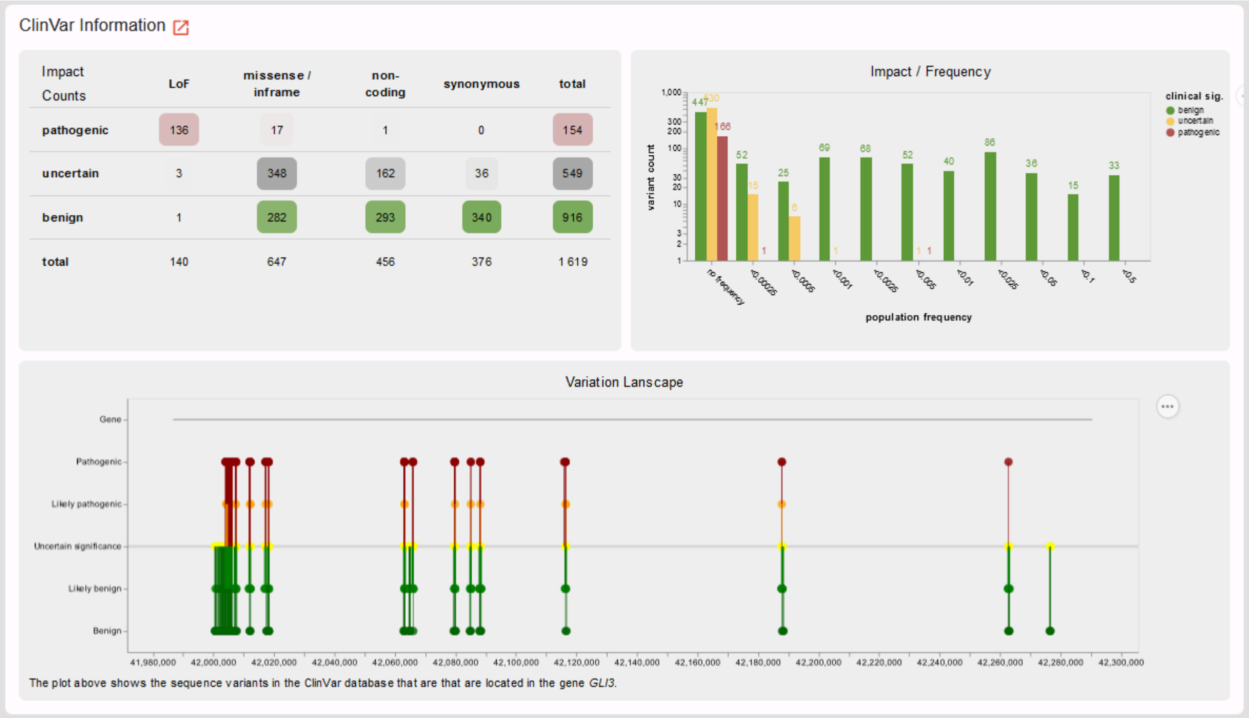

In line with the low Z-score retrieved from the gnomAD database, we see that most missense variants in ClinVar are classified as benign. However, indeed there are also some pathogenic missense variants. Time to look at the variant level and find out whether our missense variant is one of these few!

The semi-automated ACMG variant class assessment[1] based on the InterVar[2] tool tells us: we don't know…

But let's see whether we can assess this in more detail.

First, we have a more detailed look on the variant and its consequences in the different transcript variants and protein isoforms of a gene:

Here, we see that there is only one RefSeq transcript variant and our variant is located in exon 13 of 15 (- an important information e.g. also for nonsense and frameshift variants and whether or not they are subjected to nonsense mediated decay).

Is our variant a known disease variant? E.g. in the ClinVar database?

Yes, it is! Again, clicking on expand in the lower right corner and of course the link to the original ClinVar entry reveal more details:

We see that our variant is listed once in the ClinVar database as likely pathogenic for the OMIM phenotype polydactyly, postaxial type 1 (PAPA1) fitting the *GLI3*-related spectrum also fitting our patient's phenotype. We thus can assign

[1] S. Richards, N. Aziz, S. Bale, D. Bick, S. Das, J. Gastier-Foster, W. W. Grody, M. Hegde, E. Lyon, E. Spector, K. Voelkerding, and H. L. Rehm. Standards and guidelines for the interpretation of sequence variants: a joint consensus recommendation of the American College of Medical Genetics and Genomics and the Association for Molecular Pathology. *Genet Med*, 17(5):405–424, May 2015.

[2] Q. Li and K. Wang. InterVar: Clinical Interpretation of Genetic Variants by the 2015 ACMG-AMP Guidelines. *Am J Hum Genet*, 100(2):267–280, Feb 2017.

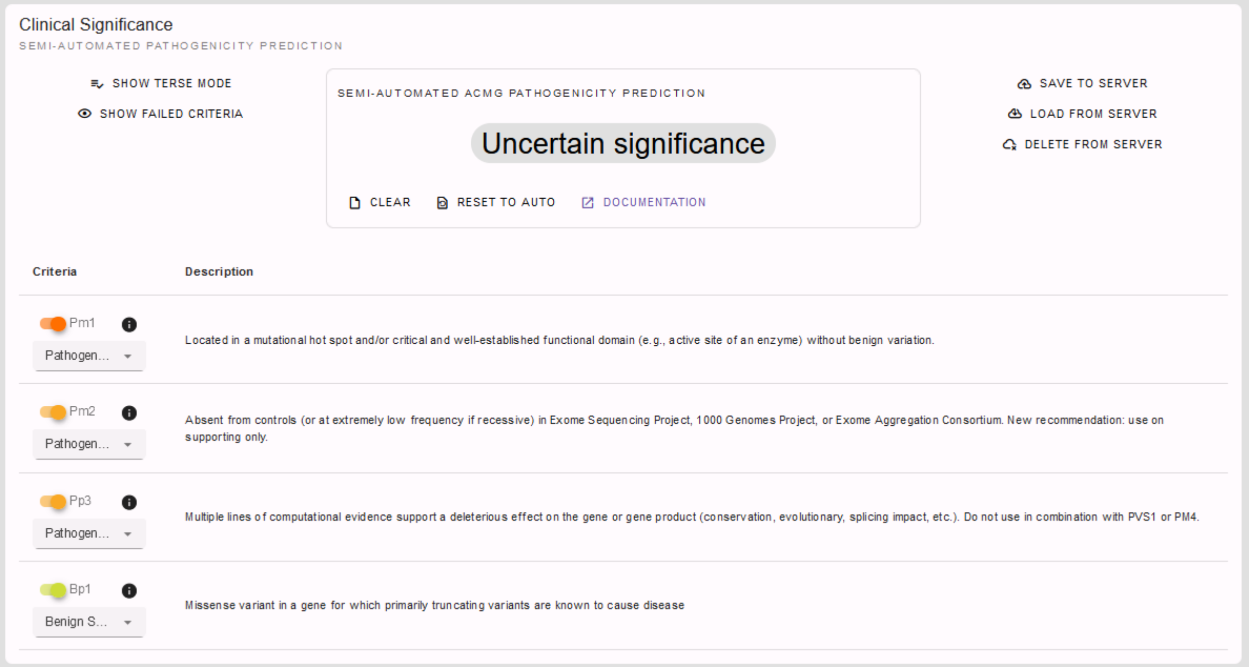
Clinical Significance
SEMI-AUTOMATED PATHOGENICITY PREDICTION
SHOW TERSE MODE
SHOW FAILED CRITERIA
SEMI-AUTOMATED ACMG PATHOGENICITY PREDICTION
Uncertain significance
CLEAR
RESET TO AUTO
DOCUMENTATION
SAVE TO SERVER
LOAD FROM SERVER
DELETE FROM SERVER
Criteria
Description
Pm1
Pathogen...
Located in a mutational hot spot and/or critical and well-established functional domain (e.g., active site of an enzyme) without benign variation.
Pm2
Pathogen...
Absent from controls (or at extremely low frequency if recessive) in Exome Sequencing Project, 1000 Genomes Project, or Exome Aggregation Consortium. New recommendation: use on supporting only.
Pp3
Pathogen...
Multiple lines of computational evidence support a deleterious effect on the gene or gene product (conservation, evolutionary, splicing impact, etc.). Do not use in combination with PVS1 or PM4.
Bp1
Benign S...
Missense variant in a gene for which primarily truncating variants are known to cause disease

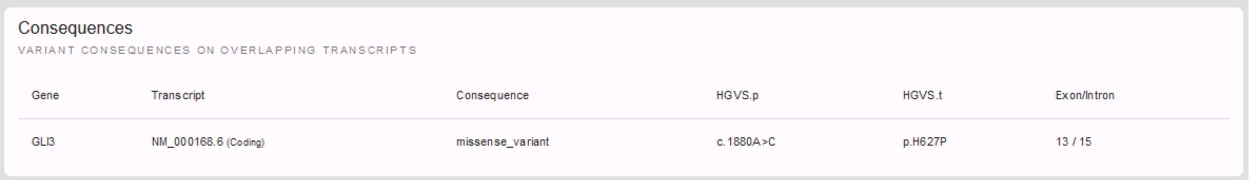
Consequences
VARIANT CONSEQUENCES ON OVERLAPPING TRANSCRIPTS
Gene
Transcript
Consequence
HGVS.p
HGVS.t
Exon/Intron
GLI3
NM_000168.6 (Coding)
missense_variant
c.1880A>C
p.H627P
13 / 15

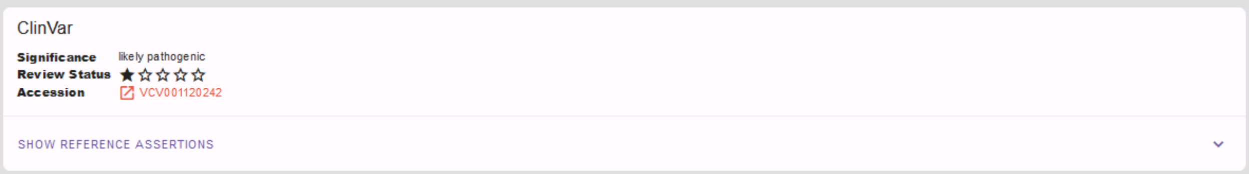
ClinVar
Significance likely pathogenic
Review Status
Accession VCV001120242
SHOW REFERENCE ASSERTIONS

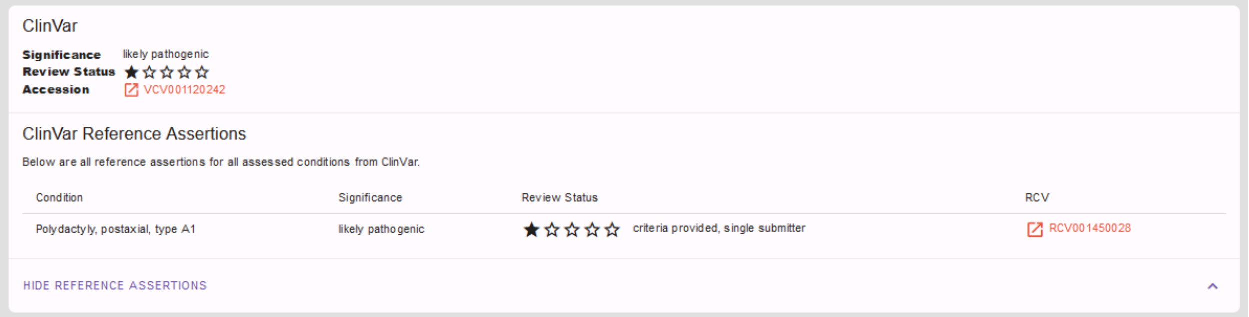
ClinVar
Significance likely pathogenic
Review Status
Accession VCV001120242
ClinVar Reference Assertions
Below are all reference assertions for all assessed conditions from ClinVar.
Condition
Significance
Review Status
RCV
Polydactyly, postaxial, type A1
likely pathogenic
criteria provided, single submitter
RCV001450028
HIDE REFERENCE ASSERTIONS

the ACMG criterion PP5 (or novel recommendation rather PS4 on supporting level) for our variant classification and adjust the ACMG rating above accordingly.

To do so, we have to select “show failed criteria” (1) in the ACMG rating section above:

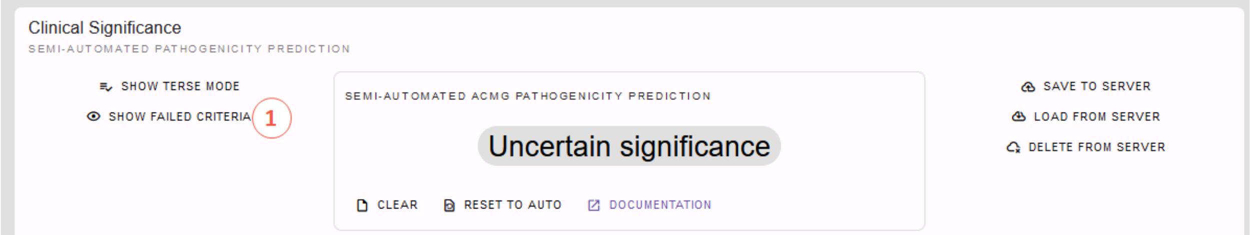

and can then set PS4 to true on supporting level:

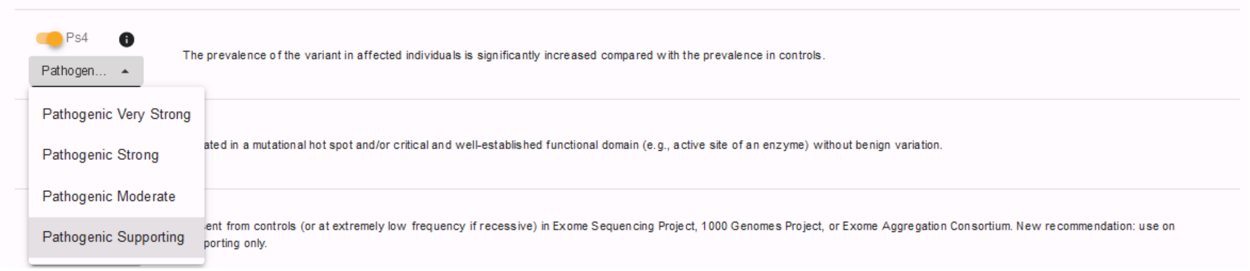

Rating the variant’s pathogenicity let’s move on to its frequency in the healthy control database gnomAD:

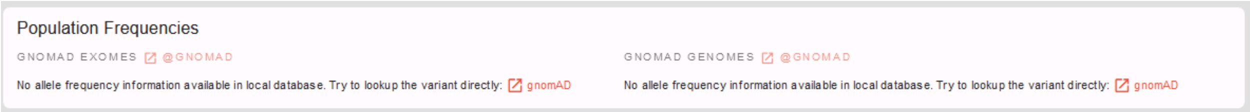

In the respective next section we see that our variant is absent from controls and can check the PM2 criterion. Note, that REEV reminds us that this criterion should be used at supporting level only according to the novel ACMG recommendations:

Next, we want to check whether our variant is predicted to be pathogenic by bioinformatic prediction tools. REEV provides us not only with an overview of the relevant prediction scores used in the assessment of SNVs, but also with the hint on which level of confidence the respective prediction can be used for ACMG criterion PP3 based on the recent ClinGen recommendations

Since they suggest that “For missense variants, to determine evidence for codes PP3 and BP4, we recommend that, for most situations, clinical laboratories use a single tool, genome-wide, that can reach the strong level of evidence for pathogenicity and moderate for benignity (BayesDel, MutPred2, REVEL, or VEST4)” and we in our lab chose to always use REVEL, we can apply the PP3 on strong level for our variant:

Note, that REEV smartly reminds us that we should not apply the PP3 criterion when PVS1 or PM4 have already been used (not the case for our missense variant here).

Also note, that beyond the PhyloP-100 score REEV also gives a nice view on conservation especially helpful for evaluating missense variants:

If we are looking at a gene with different transcript variants we can choose the one of interest in the selection box on the right.

Still missing some information? Here is more useful links REEV provides us at the end:

By clicking the “Jump in local IGV” button we can also look at our variant when we have the respective bam-file opened in our local IGV.

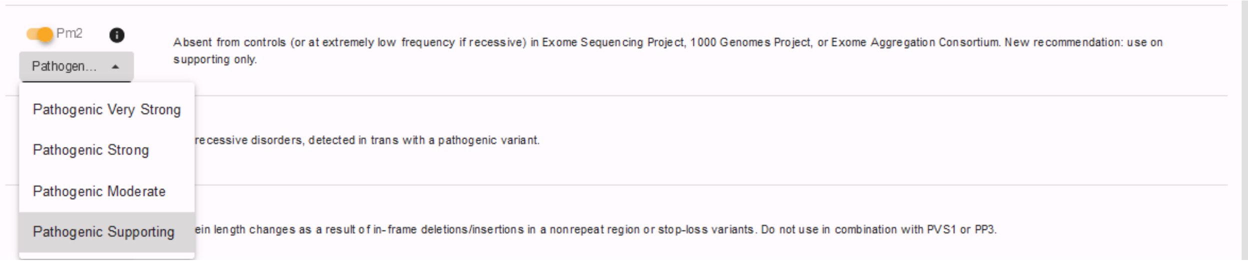
Pm2
Pathogen...
Absent from controls (or at extremely low frequency if recessive) in Exome Sequencing Project, 1000 Genomes Project, or Exome Aggregation Consortium. New recommendation: use on supporting only.
Pathogenic Very Strong
Pathogenic Strong
Pathogenic Moderate
Pathogenic Supporting
recessive disorders, detected in trans with a pathogenic variant.
ein length changes as a result of in-frame deletions/insertions in a nonrepeat region or stop-loss variants. Do not use in combination with PVS1 or PP3.

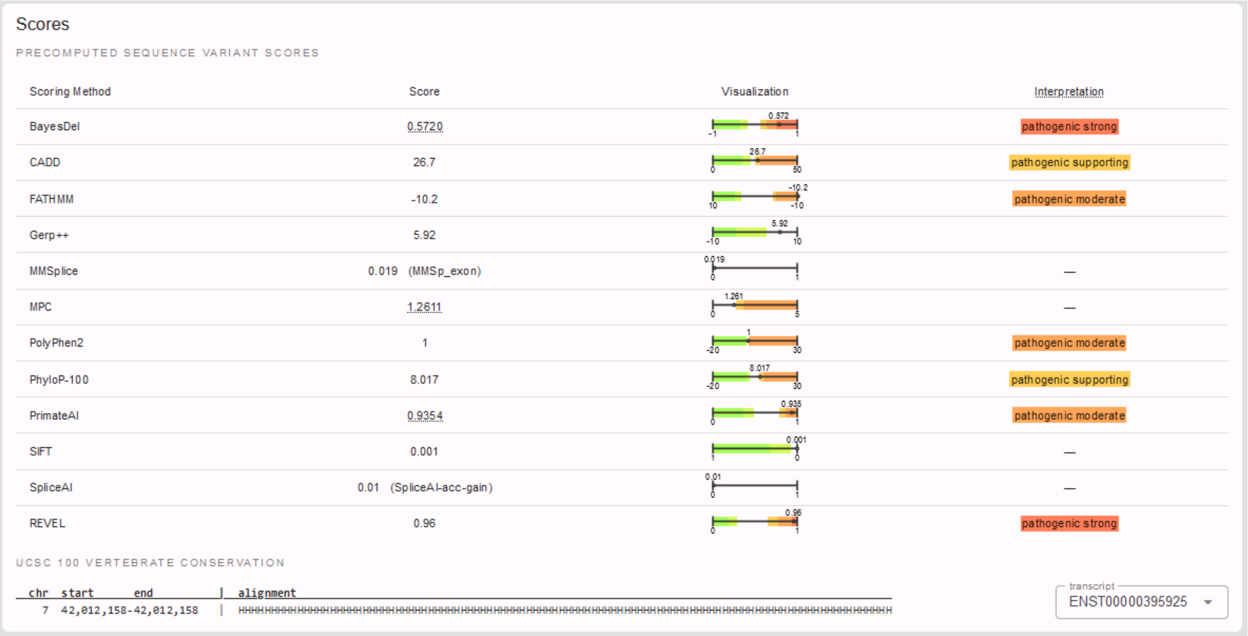
Scores
PRECOMPUTED SEQUENCE VARIANT SCORES
Scoring Method
Score
Visualization
Interpretation
BayesDel 0.5720 pathogenic strong
CADD 26.7 pathogenic supporting
FATHMM -10.2 pathogenic moderate
Gerp++ 5.92
MMSplice 0.019 (MMSp_exon)
MPC 1.2611
PolyPhen2 1 pathogenic moderate
PhyloP-100 8.017 pathogenic supporting
PrimateAI 0.9354 pathogenic moderate
SIFT 0.001
SpliceAI 0.01 (SpliceAI-acc-gain)
REVEL 0.96 pathogenic strong
UCSC 100 VERTEBRATE CONSERVATION
chr start end | alignment
7 42,012,158-42,012,158 |
transcript
ENST00000395925

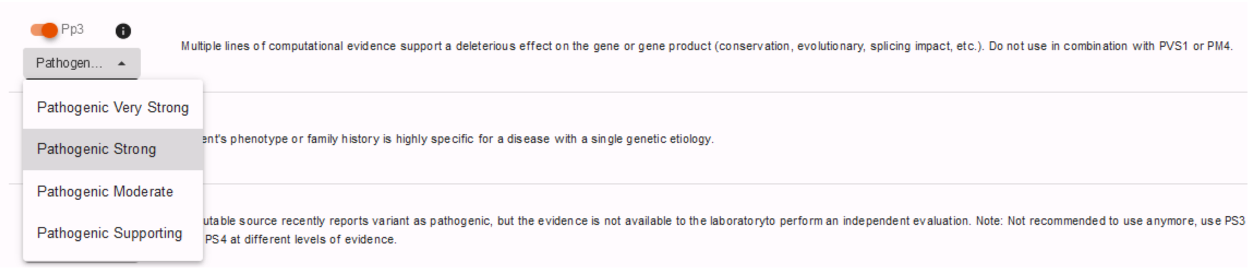
Pp3
Pathogen...
Multiple lines of computational evidence support a deleterious effect on the gene or gene product (conservation, evolutionary, splicing impact, etc.). Do not use in combination with PVS1 or PM4.
Pathogenic Very Strong
Pathogenic Strong
Pathogenic Moderate
Pathogenic Supporting
ent's phenotype or family history is highly specific for a disease with a single genetic etiology.
utable source recently reports variant as pathogenic, but the evidence is not available to the laboratoryto perform an independent evaluation. Note: Not recommended to use anymore, use PS3
PS4 at different levels of evidence.

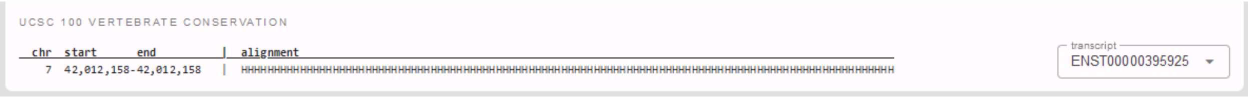
UCSC 100 VERTEBRATE CONSERVATION
chr start end | alignment
7 42,012,158-42,012,158 |
transcript
ENST00000395925

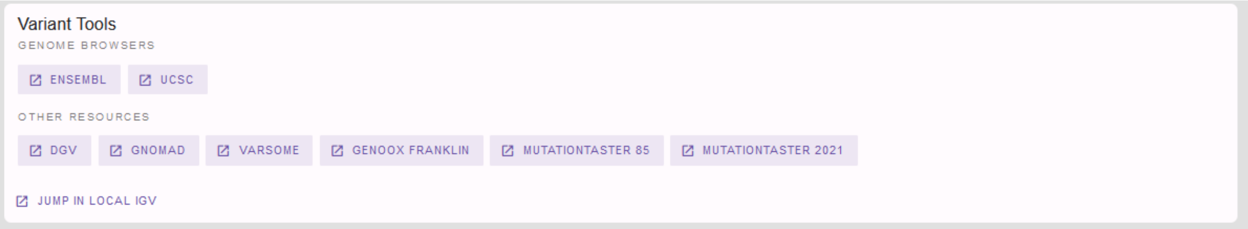
Variant Tools
GENOME BROWSERS
ENSEMBL
UCSC
OTHER RESOURCES
DGV
GNOMAD
VARSOME
GENOOX FRANKLIN
MUTATIONTASTER 85
MUTATIONTASTER 2021
JUMP IN LOCAL IGV

Last but not least, we can connect with others on our gene, variant and phenotype of interest via the beacon network (when logged in)

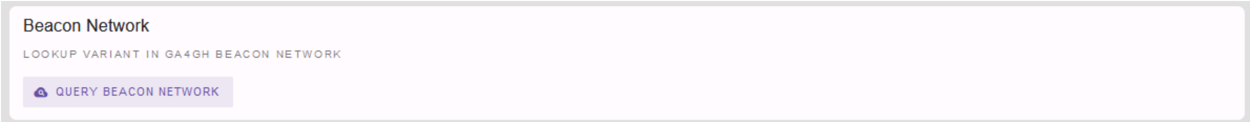

and run our variant through variant validator for a last check:

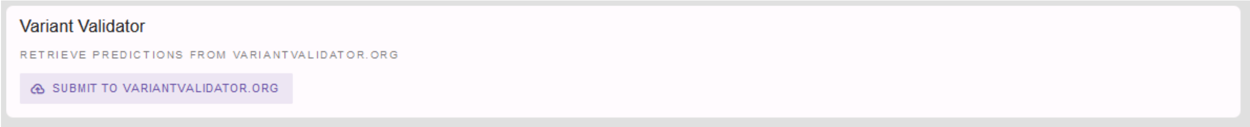

On going through this information we adjusted and added to some of the semi-automated ACMG criteria. Let's scroll back up to the ACMG rating tool and check the criteria ultimately applied and the final conclusion we came to with the help of all the information provided in REEV:

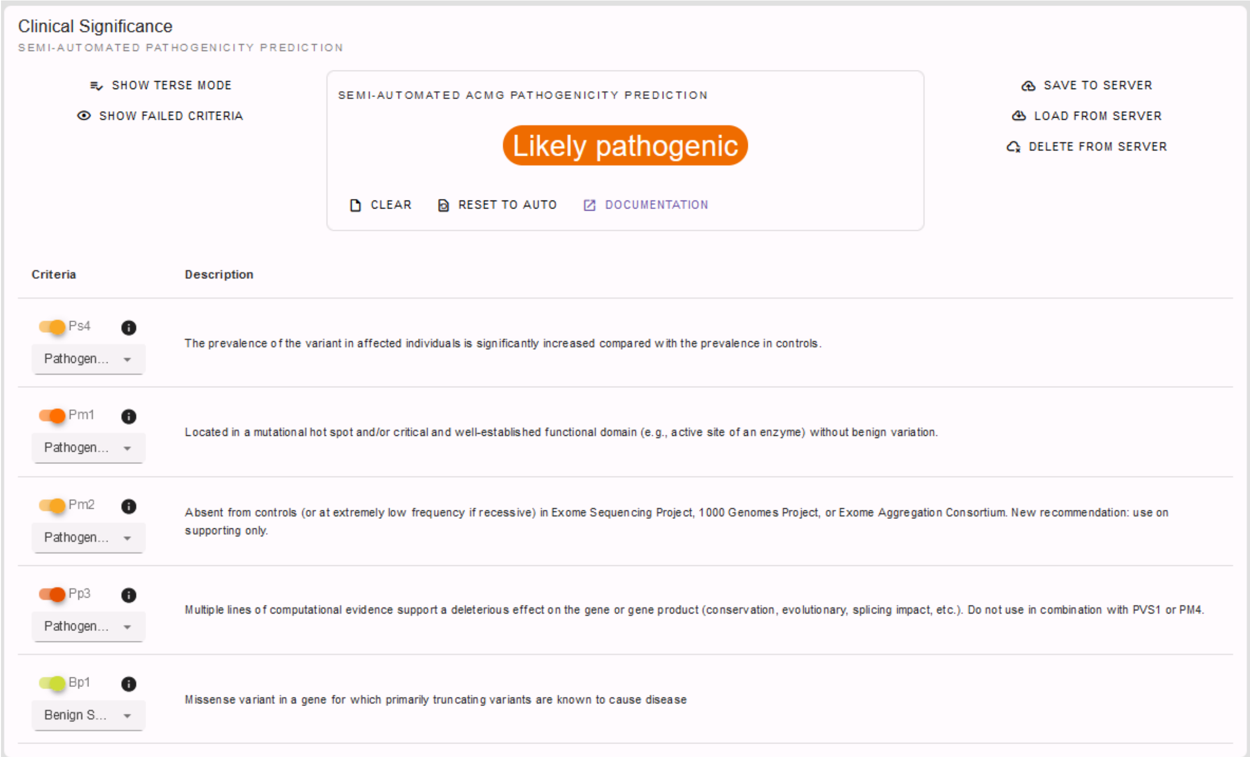

PM1, PM2, PP3 and BP1 had already been automatically selected by the InterVar[Page 15, 2] tool. However, we were able to adjust PM2 to supporting and PP3 to strong level now with the help of REEV. Additionally, we applied the PS4 criterion on supporting level as we noticed that this variant is already listed in ClinVar as a pathogenic variant with one star. So solely by the help of the information provided by REEV we were able to correct the InterVar[Page 15, 2] scoring as a variant of uncertain significance to a likely pathogenic variant!

But, of course, since we also have some clinical information on our patient at hand, we can additionally state the patient's phenotype fits the *GLI3*-related spectrum. However, there are more genes than just *GLI3* causing polydactyly, so that we apply the PP4 criterion on supporting level only. Finally, we found our variant to be de novo by segregation analysis using sanger sequencing without confirmation of paternity and maternity, so we can apply PM6 on moderate level. Using this additional information we can even conclude with a rating of our variant as pathogenic:

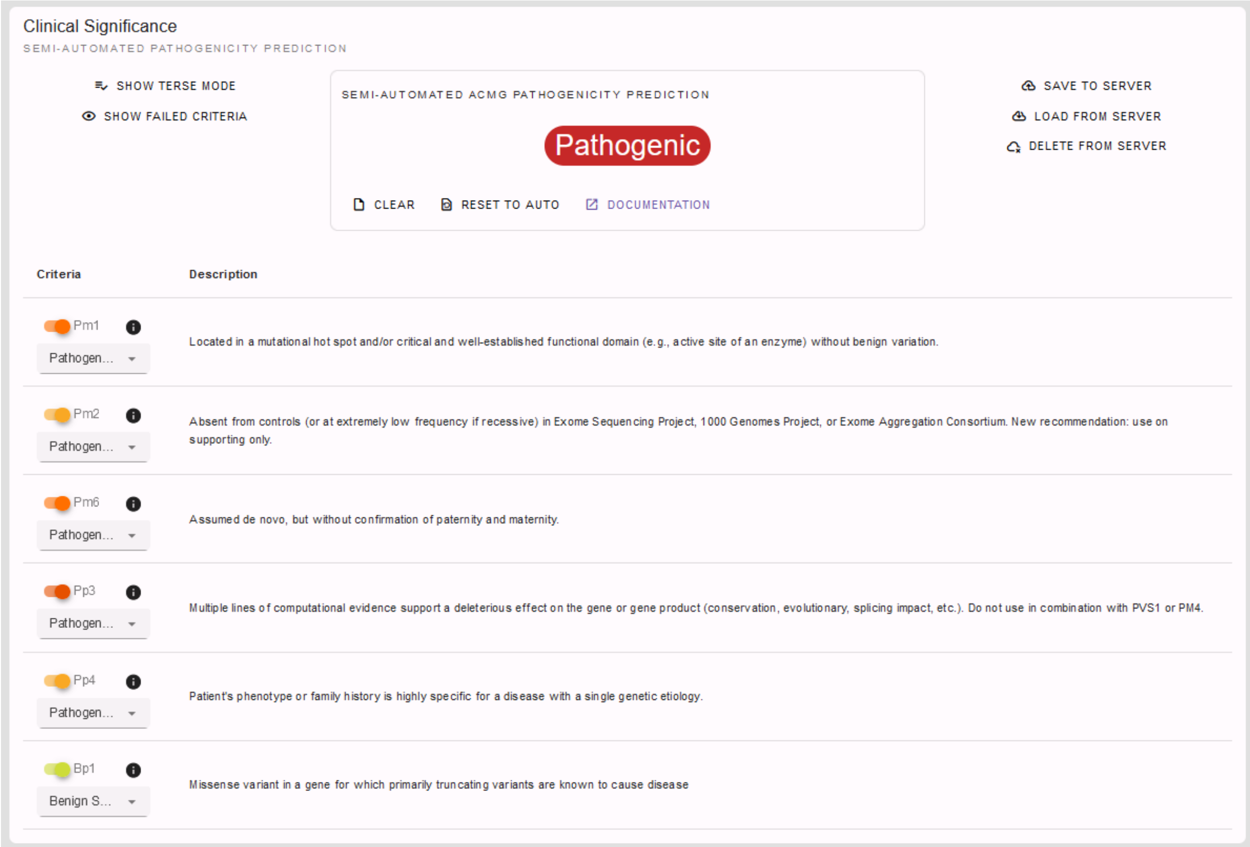

2.2.2 Structural Variants

Happy about the solved SNV case we are curious to see what REEV can provide and help us with when it comes to the trickier structural variants!

In such another case we came across the following deletion in a patient with oligodontia which we now want to explore in more detail.

Genomic variant [hg19/GRCh37]
DEL:chr14:37131998:37133815

As you can see you can enter your SV of interest by providing the affected chromosomal position preceded by the indication of the type of SV (DEL or DUP).

Note: Note, that you can also provide your variant according to ISCN. For example, this might be helpful, when you are interested in a variant that was reported in an arrayCGH test.

Let's see what REEV tells us on this structural variant. The overview shows us that this deletion affects the PAX9 gene and that this gene shows haploinsufficiency according to ClinGen, Decipher and RCNV scores as well as the gnomAD LOEUF score:

So, just one affected gene - but a potential candidate!

Note, that if we had a larger SV affecting multiple genes, REEV could help us prioritize that larger set of genes by sorting by different criteria using the sort by selection box on the right (1):

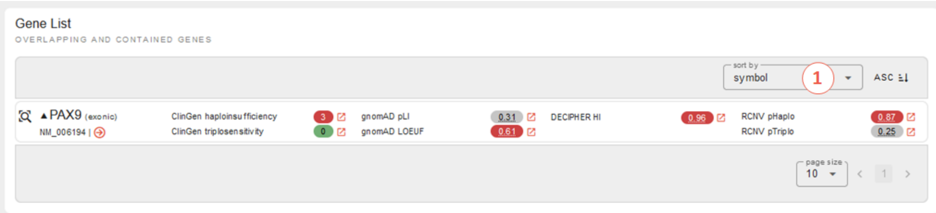

For deletions we could sort the gene list by the different haploinsufficiency scores to only look at genes that likely do not tolerate a heterozygous deletion. Vice versa, for duplications it would be helpful to sort the gene list by triplosensitivity scores.

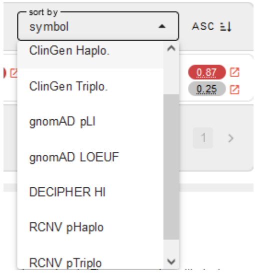

For our deletion we now know that the gene it affects is intolerant for heterozygous loss of function variants, so it could be potentially pathogenic. But is it a known disease gene? If so, does the disease fit our proband's phenotype? To answer this question, let's have a look on the gene overview and gene-phenotype-associations provided by REEV:

Those of you who started with the sequence variant tutorial already know this view - those of you who did not may find the detailed explanation in this respective section above. In brief, we find a promising gene-phenotype-association of PAX9 (1-3) with our patient's phenotype.

And again see above for the helpful display of gene expression data if PAX9 wasn't a known disease gene but might be an interesting novel candidate gene…

… as well as the display of ClinVar data you also know by now from the section above:

In line with the haploinsufficiency scores and low LOEUF score this display of ClinVar data tells us that with 29 out of 30 the vast majority of loss of function variants in PAX9 are considered pathogenic, strengthening our guess we might be onto something with our deletion here.

So, to nail that down let's have a look into the variant details:

Mhm, this is not a known ClinVar variant yet (1). But the links to ENSEMBL and UCSC (2) tell us that this variant affects exon 3 of the MANE transcript variant NM_006194.4 of the PAX9 gene which encodes for the majority of PAX9, rendering this variant a loss of function variant.

We also see that this deletion is absent from healthy controls in gnomAD (3).

I think we can all agree: a very hot variant! But can we rate it pathogenic according to the ACMG and ClinGen recommendations for copy-number variants[3]?

Semi-automated prediction from AutoCNV[4] implemented in REEV tells us it still remains unclear. We have to use our knowledge gathered from the REEV information above and our clinical knowledge on this case, let's go through the CNV criteria!

[3] E. R. Riggs, E. F. Andersen, A. M. Cherry, S. Kantarci, H. Kearney, A. Patel, G. Raca, D. I. Ritter, S. T. South, E. C. Thorland, D. Pineda-Alvarez, S. Aradhya, and C. L. Martin. Technical standards for the interpretation and reporting of constitutional copy-number variants: a joint consensus recommendation of the American College of Medical Genetics and Genomics (ACMG) and the Clinical Genome Resource (ClinGen). *Genet Med*, 22(2):245–257, Feb 2020.

[4] C. Fan, Z. Wang, Y. Sun, J. Sun, X. Liu, L. Kang, Y. Xu, M. Yang, W. Dai, L. Song, X. Wei, J. Xiang, H. Huang, M. Zhou, F. Zeng, L. Huang, Z. Xu, and Z. Peng. AutoCNV: a semiautomatic CNV interpretation system based on the 2019 ACMG/ClinGen Technical Standards for CNVs. *BMC Genomics*, 22(1):721, Oct 2021.

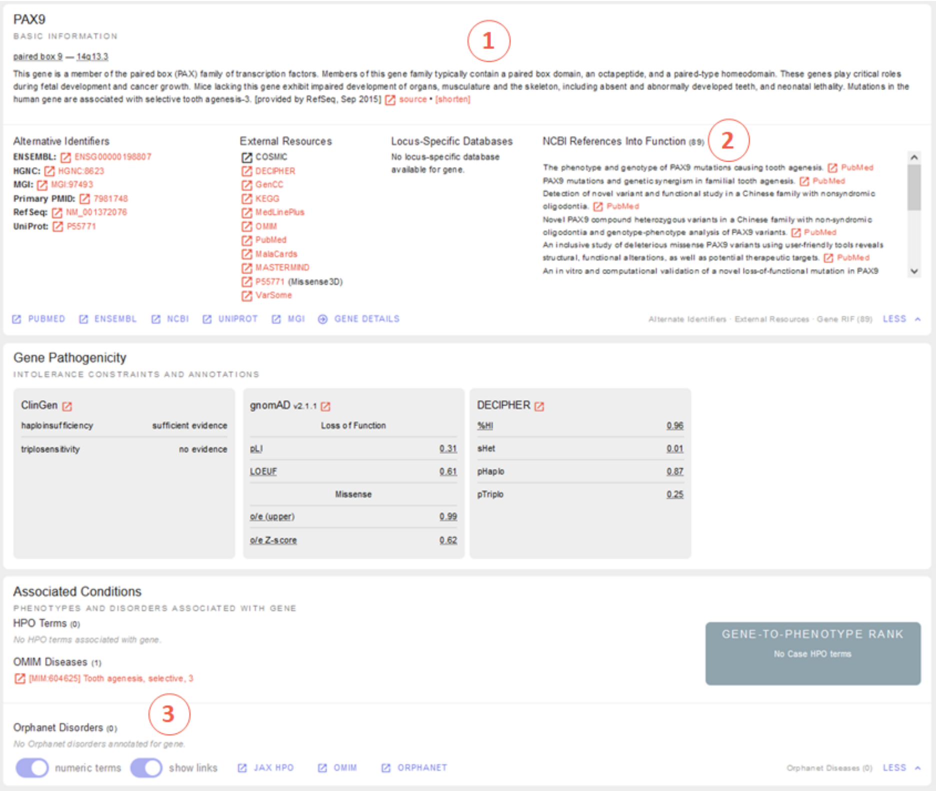
PAX9
BASIC INFORMATION
paired box 9 — 14q13.3
1
2
3
Alternative Identifiers
External Resources
Locus-Specific Databases
NCBI References Into Function (89)
Gene Pathogenicity
INTOLERANCE CONSTRAINTS AND ANNOTATIONS
ClinGen
haploinsufficiency
sufficient evidence
triplosensitivity
no evidence
gnomAD v2.1.1
Loss of Function
pLI
0.31
LOEUF
0.61
Missense
o/e (upper)
0.99
o/e Z-score
0.62
DECIPHER
%HI
0.96
sHet
0.01
pHaplo
0.87
pTriplo
0.25
Associated Conditions
PHENOTYPES AND DISORDERS ASSOCIATED WITH GENE
HPO Terms (0)
No HPO terms associated with gene.
OMIM Diseases (1)
[MIM:604625] Tooth agenesis, selective, 3
Orphanet Disorders (0)
No Orphanet disorders annotated for gene.
GENE-TO-PHENOTYPE RANK
No Case HPO terms
numeric terms
show links
JAX HPO
OMIM
ORPHANET

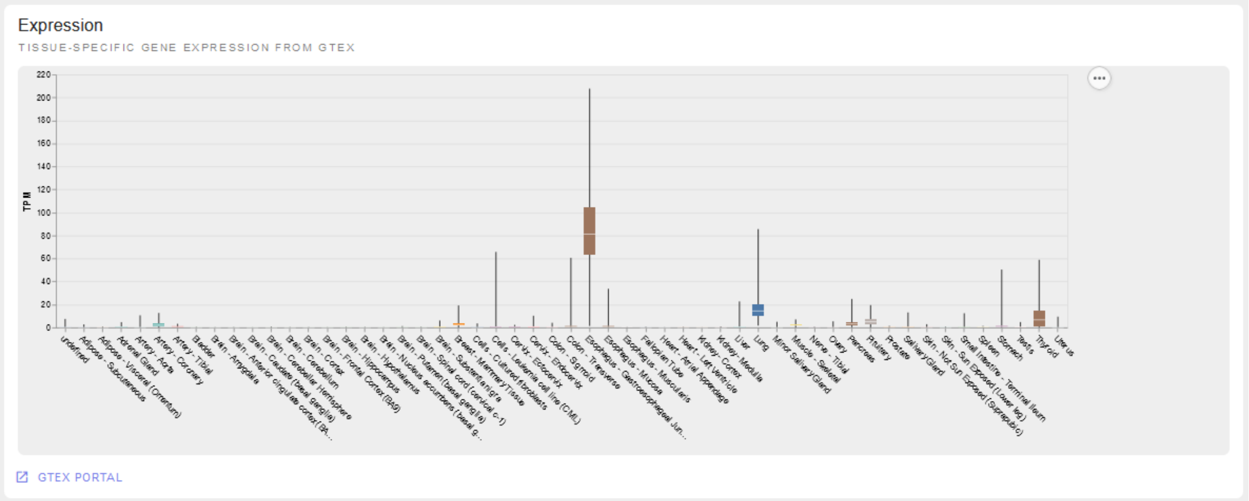
Expression
TISSUE-SPECIFIC GENE EXPRESSION FROM GTEX
TPM
GTEX PORTAL

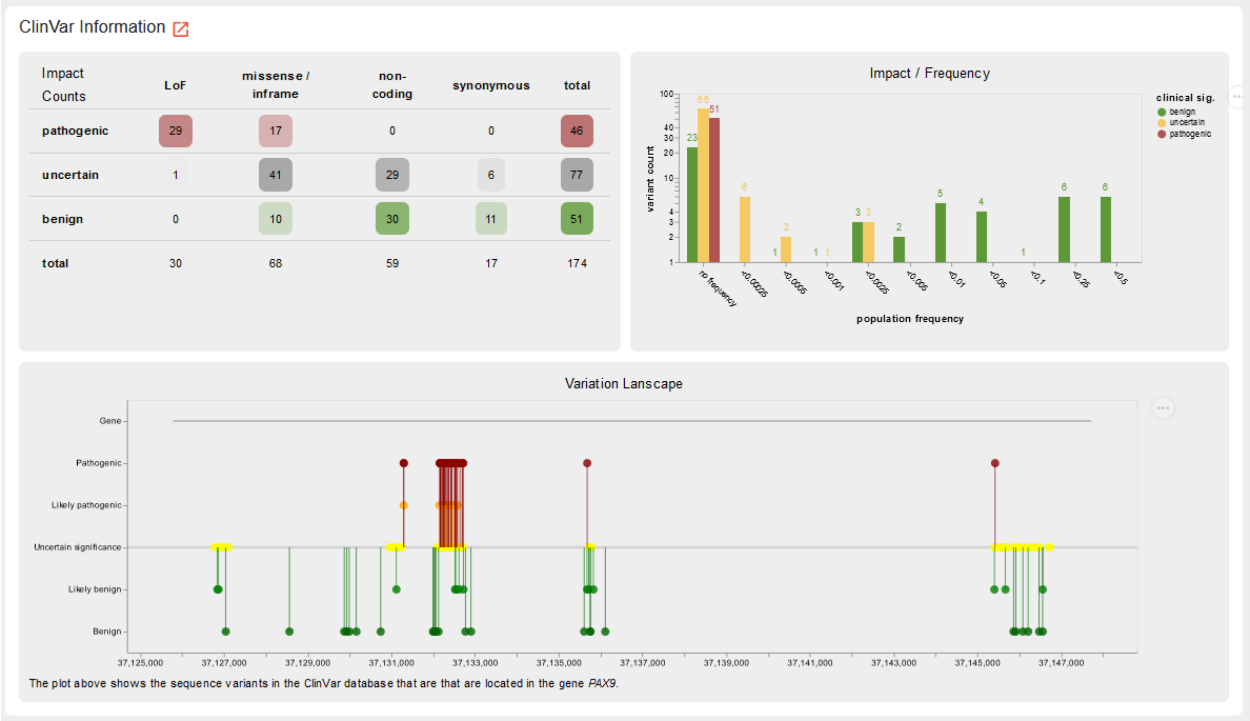
ClinVar Information
Impact Counts
LoF
missense / inframe
non-coding
synonymous
total
pathogenic 29 17 0 0 46
uncertain 1 41 29 6 77
benign 0 10 30 11 51
total 30 68 59 17 174
Impact / Frequency
variant count
population frequency
clinical sig.
benign
uncertain
pathogenic
Variation Lanscape
Gene
Pathogenic
Likely pathogenic
Uncertain significance
Likely benign
Benign
The plot above shows the sequence variants in the ClinVar database that are that are located in the gene PAX9.

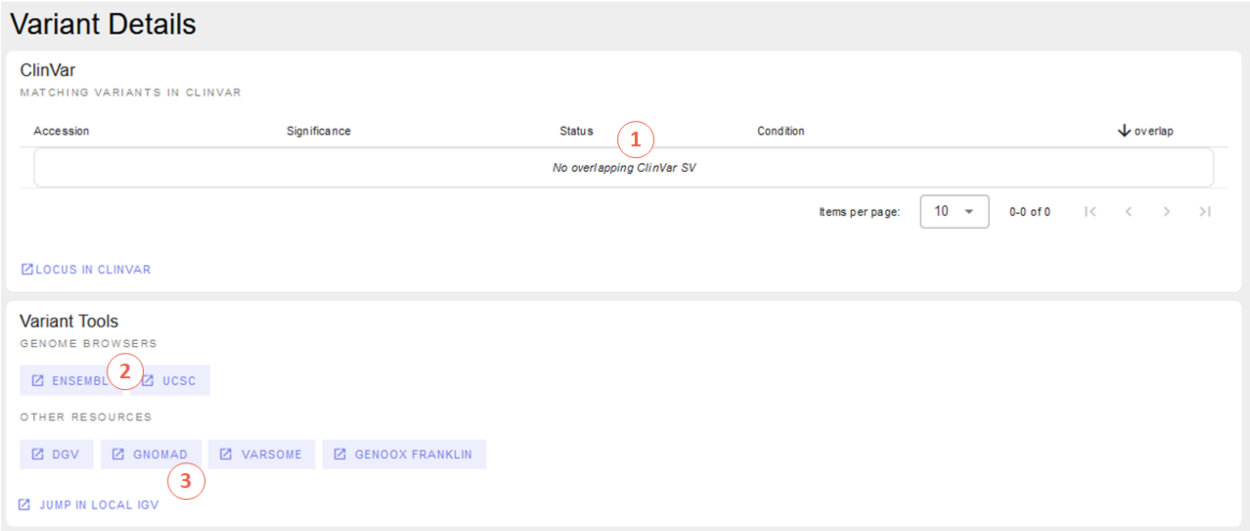
Variant Details
ClinVar
MATCHING VARIANTS IN CLINVAR
Accession
Significance
Status
Condition
overlap
1
No overlapping ClinVar SV
Items per page:
10
0-0 of 0
LOCUS IN CLINVAR
Variant Tools
GENOME BROWSERS
ENSEMBL
2
UCSC
OTHER RESOURCES
DGV
GNOMAD
VARSOME
GENOOX FRANKLIN
3
JUMP IN LOCAL IGV

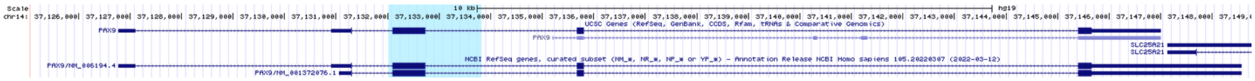

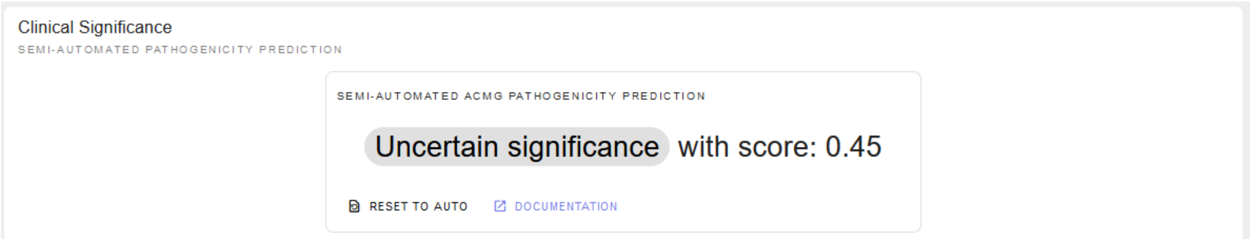
Clinical Significance
SEMI-AUTOMATED PATHOGENICITY PREDICTION
SEMI-AUTOMATED ACMG PATHOGENICITY PREDICTION
Uncertain significance with score: 0.45
RESET TO AUTO
DOCUMENTATION

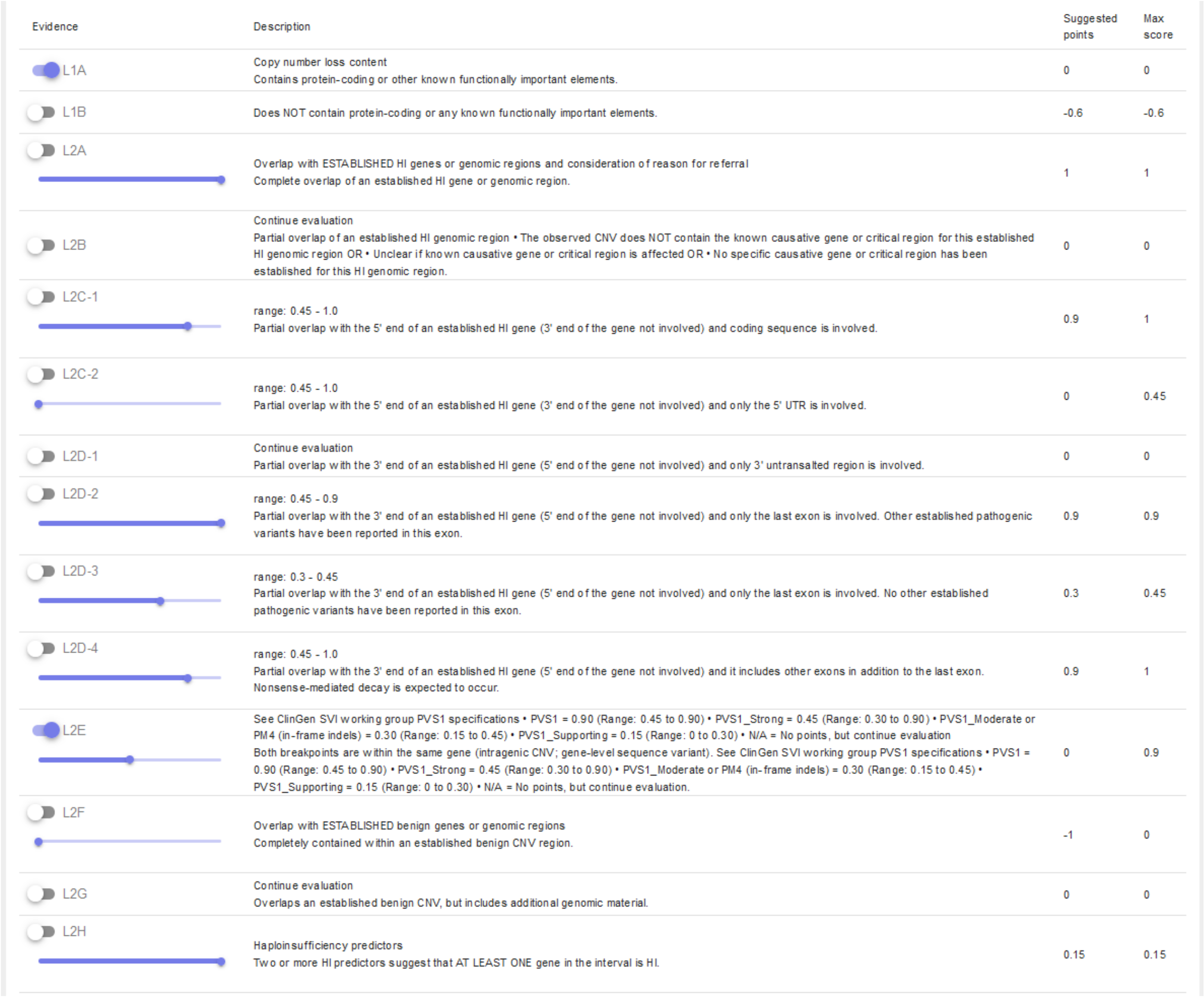

Evidence	Description	Suggested points	Max score
L1A	Copy number loss content<br>Contains protein-coding or other known functionally important elements.	0	0
L1B	Does NOT contain protein-coding or any known functionally important elements.	-0.6	-0.6
L2A	Overlap with ESTABLISHED HI genes or genomic regions and consideration of reason for referral<br>Complete overlap of an established HI gene or genomic region.	1	1
L2B	Continue evaluation<br>Partial overlap of an established HI genomic region • The observed CNV does NOT contain the known causative gene or critical region for this established HI genomic region OR • Unclear if known causative gene or critical region is affected OR • No specific causative gene or critical region has been established for this HI genomic region.	0	0
L2C-1	range: 0.45 - 1.0<br>Partial overlap with the 5' end of an established HI gene (3' end of the gene not involved) and coding sequence is involved.	0.9	1
L2C-2	range: 0.45 - 1.0<br>Partial overlap with the 5' end of an established HI gene (3' end of the gene not involved) and only the 5' UTR is involved.	0	0.45
L2D-1	Continue evaluation<br>Partial overlap with the 3' end of an established HI gene (5' end of the gene not involved) and only 3' untransalted region is involved.	0	0
L2D-2	range: 0.45 - 0.9<br>Partial overlap with the 3' end of an established HI gene (5' end of the gene not involved) and only the last exon is involved. Other established pathogenic variants have been reported in this exon.	0.9	0.9
L2D-3	range: 0.3 - 0.45<br>Partial overlap with the 3' end of an established HI gene (5' end of the gene not involved) and only the last exon is involved. No other established pathogenic variants have been reported in this exon.	0.3	0.45
L2D-4	range: 0.45 - 1.0<br>Partial overlap with the 3' end of an established HI gene (5' end of the gene not involved) and it includes other exons in addition to the last exon. Nonsense-mediated decay is expected to occur.	0.9	1
L2E	See ClinGen SVI working group PVS1 specifications • PVS1 = 0.90 (Range: 0.45 to 0.90) • PVS1_Strong = 0.45 (Range: 0.30 to 0.90) • PVS1_Moderate or PM4 (in-frame indels) = 0.30 (Range: 0.15 to 0.45) • PVS1_Supporting = 0.15 (Range: 0 to 0.30) • N/A = No points, but continue evaluation<br>Both breakpoints are within the same gene (intragenic CNV; gene-level sequence variant). See ClinGen SVI working group PVS1 specifications • PVS1 = 0.90 (Range: 0.45 to 0.90) • PVS1_Strong = 0.45 (Range: 0.30 to 0.90) • PVS1_Moderate or PM4 (in-frame indels) = 0.30 (Range: 0.15 to 0.45) • PVS1_Supporting = 0.15 (Range: 0 to 0.30) • N/A = No points, but continue evaluation.	0	0.9
L2F	Overlap with ESTABLISHED benign genes or genomic regions<br>Completely contained within an established benign CNV region.	-1	0
L2G	Continue evaluation<br>Overlaps an established benign CNV, but includes additional genomic material.	0	0
L2H	Haploinsufficiency predictors<br>Two or more HI predictors suggest that AT LEAST ONE gene in the interval is HI.	0.15	0.15

We find that criteria L1 and L2 are checked correctly for L1A and L2E true. Note, that we have to use L2E because both breakpoints of our deletion are within the same gene and that L2E is assigned with 0.45 points which is also the correct value: Since the deletion abrogates more than 50% of the protein coding sequence we can consider it a loss of function variant and assign the ACMG criterion PVS1 on strong level, equaling a score of 0.45.

L3 is an easy one and also pre-checked correctly to L3A:

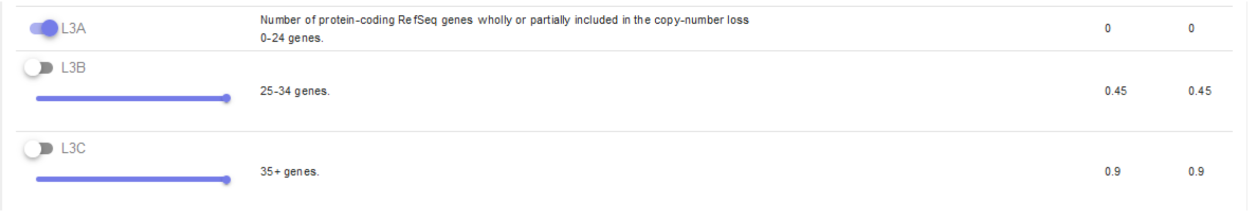

Let's continue on to the manual case specific part:

REEV showed us 29 (likely) pathogenic loss of function SNVs in the ClinVar database (see screenshot above). As we have no information on inheritance from this ClinVar data and there are no matching SVs reported yet, we can only give the max. 0.3 points at L4E here:

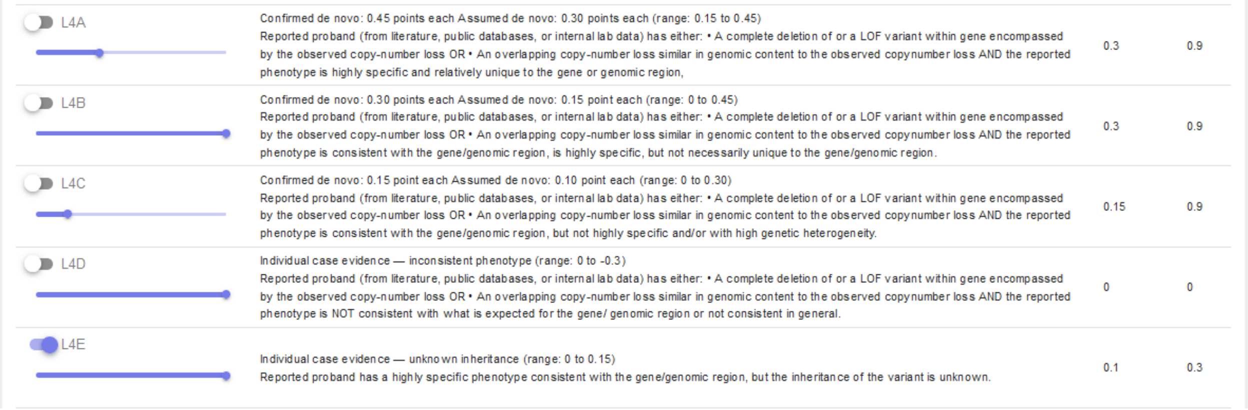

But we do have inheritance information on our case, of course! Here, the PAX9 deletion occurred de novo in a patient with oligodontia and their parents are known to be healthy. So, we can assign L5A with a score of 0.45:

Let's scroll back up and see where we have landed on rating this variant with the help of REEV!

Another solved case it is for us and REEV!

2.2.3 User login

All of the above is possible without any login. Beyond this, a login to REEV offers additional helpful features.

With creating a login you can:

- Store case specific information which can be helpful in evaluating your variant. E.g., you can add phenotypic information on your case by providing the corresponding HPO terms (see also above in the OMIM and HPO section of the sequence variant tutorial). On having provided HPO terms, a gene-to-phenotype rank will be provided to help you evaluate how well your patient's phenotype and the known gene-disease association fit one another.
- Bookmark genes and variants of interest to return to them at a later time point.
- Submit your evaluated variant to ClinVar (see section *ClinVar Submission*).

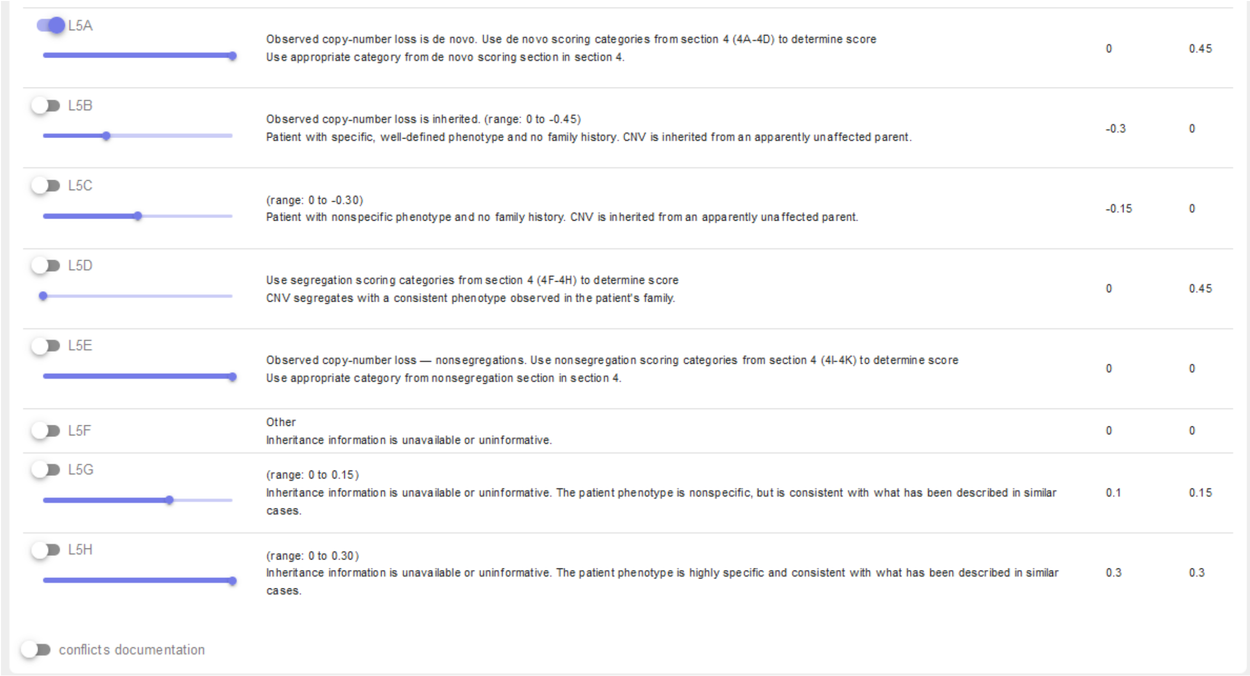
L5A
Observed copy-number loss is de novo. Use de novo scoring categories from section 4 (4A-4D) to determine score
Use appropriate category from de novo scoring section in section 4.
0
0.45
L5B
Observed copy-number loss is inherited. (range: 0 to -0.45)
Patient with specific, well-defined phenotype and no family history. CNV is inherited from an apparently unaffected parent.
-0.3
0
L5C
(range: 0 to -0.30)
Patient with nonspecific phenotype and no family history. CNV is inherited from an apparently unaffected parent.
-0.15
0
L5D
Use segregation scoring categories from section 4 (4F-4H) to determine score
CNV segregates with a consistent phenotype observed in the patient's family.
0
0.45
L5E
Observed copy-number loss — nonsegregations. Use nonsegregation scoring categories from section 4 (4I-4K) to determine score
Use appropriate category from nonsegregation section in section 4.
0
0
L5F
Other
Inheritance information is unavailable or uninformative.
0
0
L5G
(range: 0 to 0.15)
Inheritance information is unavailable or uninformative. The patient phenotype is nonspecific, but is consistent with what has been described in similar cases.
0.1
0.15
L5H
(range: 0 to 0.30)
Inheritance information is unavailable or uninformative. The patient phenotype is highly specific and consistent with what has been described in similar cases.
0.3
0.3
conflicts documentation

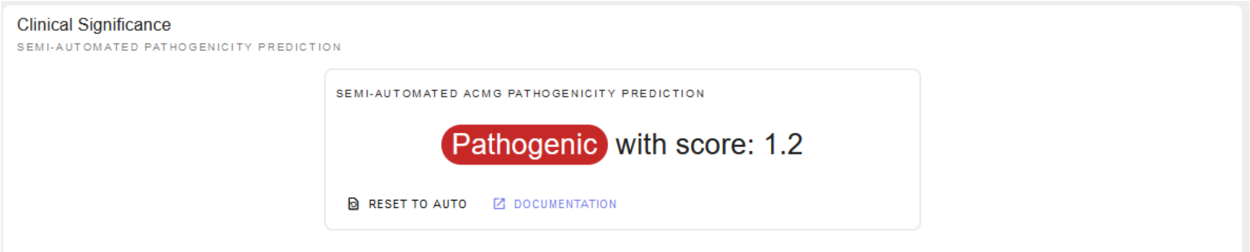
Clinical Significance
SEMI-AUTOMATED PATHOGENICITY PREDICTION
SEMI-AUTOMATED ACMG PATHOGENICITY PREDICTION
Pathogenic with score: 1.2
RESET TO AUTO
DOCUMENTATION

To login, click on "Login" button on the top right of the REEV homepage. You can then log into REEV with an ORCID or LifeScience Research Infrastructure account. Just click the corresponding button the login screen.

- ORCID is an organisation that provides persistent digital identifiers and creating an account with them is free. Go to https://orcid.org for more information.
- LifeScience Research Infrastructure allows researchers from the European Union to login with the account from their home organization. See https://lifescience-ri.eu/ls-login/ for more information.

2.3 Supported Queries

This section describes the queries that are supported by REEV.

2.3.1 Genes

Gene queries in REEV allow users to search by HGNC symbol or NCBI/ENSEMBL/HGNC ID. Examples of gene queries include:

- *BRCA1*: Search by HGNC symbol.
- *HGNC:1100*: This refers to the HGNC ID for the BRCA1 gene.
- *7273*: This is the NCBI ID for the TTN gene.

2.3.2 Sequence Variants

Sequence variant queries in REEV can be provided in HGVS notation, gnomAD style, or SPDI-style. Examples include:

- *NM_007294.4(BRCA1):c.5123C>A*: HGVS transcript SNV.
- *NM_000179.3:c.4082del*: HGVS transcript deletion.
- *NC_000017.10:g.41197728G>T*: HGVS genomic SNV.
- *GRCh37-chr17-41197751-G-T*: gnomAD style variant.
- *NC_000017.11:43039470:G:A*: Canonical SPDI.

2.3.3 Structural Variants

Structural variant queries in REEV can be provided in various formats including ISCN notation, colon-separated, or hyphen-separated formats. Examples include:

- *DEL:chr17:41176312:41277500*: Deletion on chromosome 17.
- *DUP-chrX-73565114-73956354*: Duplication on chromosome X.
- *arr[GRCh37] 7q11(72,650,120_74,154,209)x1*: ISCN notation.
- *GRCh37 7q11(72,650,120_74,154,209)x1*: Shorter form of ISCN notation.

2.4 Front-End URLs

This section describes the stable part of the front-end's URLs (aka web addresses). It includes a description how the search/lookup works in REEV and the supported query formats.

Overall, the front-end allows to lookup information on:

- sequence variants (`seqvars`, we consider all variants <50bp as small and these are also known as SNVs, SNPs, indels, etc.)
- structural variants (`strucvars`, where we currently support detailed information only on deletions and duplications, aka copy number losses and gains)
- individual genes

This section first describes the representation of the entities that REEV can be queried for. It then continues with the description of the URLs and the query engine behaviour.

2.4.1 Aims and Background

The aim of the variant/gene query (or better fitting: resolution) engine is to provide users with an intuitive/natural way to search for variants (and genes). The user input has to fulfill certain requirements, e.g., not contain too much extra whitespace. The description below includes a summary of what normalization is performed.

Note that the user generally has to select one of the supported genome releases GRCh37 or GRCh38, even for genes, the default currently is GRCh37. The reason for this is that reference genome specific information will also be displayed

The start page of REEV contains a number of examples for valid queries that should illustrate the supported formats.

2.4.2 Sequence Variants

Note: dbSNP and ClinVar accession translation has not been implemented yet.

Sequence variants are supported in the following formats:

HGVS genomic variants (`g.` format)

For example: `NC_000017.10:g.48275363C>A` or `NC_000017.11:g.50198002C>A`. Note that the sequence identifier are case sensitive must be versioned. The genome version will be derived from the sequence identifiers.

HGVS transcript variants (either `c.` or `n.` format)

For example: `NM_000088.4:n.653G>T` or `NM_000088.4:c.535G>T`. Here, the genome release will be needed to extract the genome reference information.

gnomAD variant identifiers

For example: `1-55516888-G-A`. Here, we will need explicit genome release information as well. You can also provide this, e.g., with `GRCh37-1-55516888-G-A`.

Canonical SPDI

This is the format `SEQUENCE:POSITION:REF:ALT` where `SEQUENCE` is the GenBank sequence name of the GRCh38 release. For example: `GRCh38:17:50198002:C:A` or `GRCh37:17:48275363:C:A`.

SPDI with genome release

You can also use RELEASE:CHROM for the SEQUENCE name above. Example: GRCh38:10:87925523:C:G. If you leave out the release name and just use CHROM then the genome release from the search field will be used, falling back to GRCh37.

dbSNP Identifier

This is the format rs<NUMBER> where <NUMBER> is the dbSNP identifier. This is case insensitive. For example: rs123456.

ClinVar identifier

This is RCV<NUMBER> and VCV<NUMBER>. Optionally, you can specify a .<VERSION> suffix which is subsequently ignored.

Overall, we support the following genome release identifiers (case insensitive) of the GRCh37 and GRCh38 releases. GRCh37, GRCh38, hg19, hg38. Only canonical chromosomes 1..22, X, Y, MT are supported.

2.4.3 Structural Variants

You can specify deletions and duplications as follows:

Colon-separated format

TYPE:SEQUENCE:START where SEQUENCE can be an NCBI identifier, the RELEASE:CHROM, or just CHROM as for sequence variant SPDI. Examples: DEL:NC_000017.10:48275363 or DEL:GRCh37:17:48275363 or DUP:17:48275363. Optionally, you can also use a the gnomAD-style variant identifier and use a hyphen rather than a color and, e.g., write DEL-GRCh37-17-48275363

ISCN array format

These follow the format arr[$RELEASE] $CHROM.$CHROME($START_$STOP)x$COPY. `$RELEASE is the genome release, $CHROMB is the CHROM{p,q}BAND of the start position, similarly $CHROME for the end chromosome. $START and $STOP are the start and stop positions, $COPY is the copy number. Examples: arr[GRCh37] 2q12.2q13(107132950_110427254)x1 for a deletion or or arr[hg38] 17q23.1-25.1(chr17:36449220-75053130)x3 for a duplication. You can also just specify $RELEASE rather than arr[$RELEASE].

2.4.4 Genes

You can lookup genes by:

- their approved HGNC symbols
- alias symbols as provided by HGNC
- previous symbols registered with HGNC
- the official HGNC identifier

All gene search is case insensitive.

For example, see the HGNC page on the *VPS13B* gene. The approved symbol is *VPS13B*, the only alias symbol is *BLTP5B`, previous symbols are *CHS1 and *COH1*, and the HGNC identifier is HGNC:2183 So the following queries would bring you to the gene *VPS13B*:

- VPS13B
- VPS13b
- BLTP5B
- CHS1

- `COH1`
- `coh1`
- `coH1`
- `HGNC:2183`

2.4.5 URLs

REEV provides the following stable entrypoint URLs.

`/query?q=${TERM}&genomeRelease=${GENOME}`

Query endpoint where `TERM` can be in any of the supported formats above. See below for the documented behaviour.

`/gene/${GENOME}/${SYMBOL}`

Canonical URLs of genes with the official HGNC symbol. When loaded, the symbol will be checked against the HGNC database. If we only find a match for an alias or previous symbol, the user will be redirected to the official symbol.

`/seqvar/${GENOME}/${CHROM}-${POS}-${DEL}-${INS}`

Canonical URLs of sequence variants in gnomAD-style SPDI format. Currently, no check is performed whether `DEL` sequence is correct in the reference.

`/strucvar/${GENOME}/(DEL|DUP)/${CHROM}-${START}-${STOP}`

Canonical URLs of deletions and insertions.

2.4.6 Resolving Queries

The resolution of gene names is done by a backend service, as is the parsing of HGVS variants and projection to gnomic coordinates. The remaining query formats are directly resolved by the query engine by parsing the data. The query engine will perform the following steps.

1. Attempt to interpret as one of the known sequence variant formats.
   a. gnomAD variant identifier
   b. SPDI
   c. dbSNP identifier
   d. ClinVar identifier
2. Attempt to interpret as one of the known structural variant formats.
   a. Colon-separated format
   b. ISCN array format
3. Attempt to interpret as HGVS notation with backend service.
4. Attempt to find a perfect case sensitive match for a gene symbol, alias, or previous symbol.
5. Attempt to find a partial match for a gene symbol, alias, or previous symbol.

2.5 Manual

This section describes the data display in detail.

2.5.1 Genes

Basic information

This card shows the basic information for a gene such as its symbol, name, and a short summary that we took from the NCBI gene overview. At the bottom of the card, you can find link-outs to other websites describing the gene, such as searching pubmed, ENSEMBL, NCBI, UniProt and MGI. At the very right of this link-outs you find a link to REEV's gene detail view (detailed view of a gene's properties without any specific variant information).

Note that you can also click on "More" on the lower right of the card to show additional information on the gene:

- Alternative identifiers with link-outs, e.g., to ENSEMBL, or RefSeq.
- Further external resources such as DECIPHER, OMIM, etc.
- Link-outs to locus specific databases if available for the respective gene of interest, e.g. LOVD, NIH-BIC database (for BRCA1/2 variants), Leiden Muscular Dystrophy pages (for TTN variants), etc.
- NCBI GeneRIFs

Gene Pathogenicity

This card shows the avaible gene pathogenicity scores for a gene such as:

- ClinGen haploinsufficiency and triplosensitivity scores
- gnomAD pLI and LOEUF scores (for loss of function variants) and Z-score (for missense variants)
- DECIPHER haploinsufficiency and triplosensitivity scores as well as sHet (Selection coefficient of heterozygous loss-of-function variants) scores

Associated Conditions

This card shows the avaible information on the conditiosn associated to the respective gene.

First, the card will display information on membership in the ACMG Supplementary Findings list, if the gene is contained.

Second, the card will show diseases associated via OMIM, OrphaNet or PanelApp. The confidence corresponds to the highest evidence of the link (high, medium, or low). A link from OMIM has high confidence, as does have a link from OrphaNet with "Assessed" label. Orphanet links that are "Not yet assessed" have a link of low strength. Link strength from PanelApp are taken from the green/amber/red labels in PanelApp. Currently, links from PanelApp are only made if the gene-phenotype link is tagged with an Orphanet or OMIM disease. Clicking on the disease sheet will show you the OMIM and Orphanet terms that are integrated into the card.

Below, you can see a list of PanelApp panels associated with the gene. You can see the panel name, the version, confidence, and labeled mode of inheritance. Clicking on the panel sheet will show you the full list of phenotypes labeled in the panel.

Note than you can use the button "numeric terms" in the lower left corner to show or hide the corresponding numeric HPO term and OMIM phenotype IDs; with the button "show links" right next you can activate and deactivate the corresponding link-outs of the displayed HPO terms and OMIM phenotypes. At the bottom of the card you can also find more link-outs to further websites with gene-phenotype information, such as JAX and Orphanet. Note that you

can also use the “More” button on the lower right of the card to directly show non-integrated OMIM and Orphanet disorders and their respective link-outs.

If you have logged in and provided case specific phenotype information with HPO terms you can find the gene-to-phenotype rank (i.e. how well your case’s HPO terms fit the gene’s associated HPO terms) in the grey box on the right.

Literature

This card displays the latest ten publications in PubMed related to this gene. For more genes, follow the link-out to PubTator.

Expression

This card shows the tissue specific information for a gene as provided by GTEX. Below the plot you also find the corresponding link-out to the GTEX portal. Note that you can also use the little “...” button in the upper right corner of the plot to

- save the plot as .svg or .png
- view the corresponding vega source code and open in vega editor

ClinVar Information

This card shows aggregate variant information from ClinVar for the current gene.

Impact Counts

This table gives summary counts of variants by classification and variant type as found in ClinVar. The rows are labeled as pathogenic/likely pathogenic (row “pathogenic”), uncertain significance, and benign/likely benign (row “benign”) in ClinVar for the current gene. The columns are labeled as loss of function (Lof), missense/inframe, non-coding, and synonmous. The marginal rows and columns contain row-/column-wise sums.

The cells have a colored background (red/gray/green in the rows pathogenic/uncertain/benign). The color intensity is proportional to the column-wise fraction. The maximal intensity is reached at 20% of the column counts. For example, if there is a total of 100 variants in the LoF column, with 30 pathogenic, 60 uncertain, and 10 benign, then the color intensity will be full for pathogenic and uncertain, and be at 50% intensity for benign.

Overall, this allows you to quickly gauge the distribution of variant classifications for different variant types in ClinVar. The mapping from variant type in ClinVar to column label is as follows:

- unknown: not shown
- stop lost: not shown
- no sequence alteration: synonymous
- synonymous variant: synonymous
- 3’ UTR variant: non-coding
- 5’ UTR variant: non-coding
- downstream transcript variant: non-coding
- intron variant: non-coding
- non-coding transcript variant: non-coding

- upstream transcript variant: non-coding
- inframe indel: missense/inframe
- missense variant: missense/inframe
- frameshift variant: LoF
- start lost: LoF
- stop gained: LoF
- splice acceptor variant: LoF
- splice donor variant: LoF

Impact / Frequency

This plot shows the number of variants with the coarse clinical significances pathogenic (pathogenic/likely pathogenic), uncertain significance, and benign (benign/likely benign) in ClinVar for the current gene. These are shown in buckets of population frequency (gnomAD AF) as provided by ClinVar. The bucket boundaries are 0.00025, 0.0005, 0.001, 0.0025, 0.005, 0.01, 0.025, 0.05, 0.1, 0.25, 0.5, 1.0.

Overall, this allows you to quickly see the frequency distribution of variants in ClinVar for the current gene.

Variation Landscape

This plot shows the distribution of pathogenic, likely pathogenic, uncertain significance, likely benign, and benign variants in ClinVar along the current gene. The line "gene" displays the exons of the gene. You can use this to see whether there are any hotspots of pathogenic variants in ClinVar for the current gene.

2.5.2 Sequence Variants

Clinical Significance

This card shows the semi-automated pathogenicity prediction based on InterVar[1]. Using the buttons on the left you can…

- Hide/show the terse mode of ACMG criteria[2] display. On show this will provide you an overview of just the different criteria and their evidence level, on hide you will see the full display also providing a description on every ACMG criterion and how it should be used.
- Hide/show failed criteria (not set to "active" by the little switch displayed left to every criterion)

Tipp: you can also see are brief description of every ACMG criterion in the terse mode when moving your curser of the little "i" in the upper right corner of every ACMG criterion box.

On default you will see the automated selection of ACMG criteria. You can individually select and deselect every ACMG criterion using the little switch displayed left to every criterion and also select the respective level of evidence (very strong, strong, moderate, supporting) using the button below the respective ACMG criterion.

[1] Q. Li and K. Wang. InterVar: Clinical Interpretation of Genetic Variants by the 2015 ACMG-AMP Guidelines. *Am J Hum Genet*, 100(2):267–280, Feb 2017.

[2] S. Richards, N. Aziz, S. Bale, D. Bick, S. Das, J. Gastier-Foster, W. W. Grody, M. Hegde, E. Lyon, E. Spector, K. Voelkerding, and H. L. Rehm. Standards and guidelines for the interpretation of sequence variants: a joint consensus recommendation of the American College of Medical Genetics and Genomics and the Association for Molecular Pathology. *Genet Med*, 17(5):405–424, May 2015.

Note that you can also clear all selected ACMG criteria or reset them to auto using the respective buttons below the pathogenicity prediction box on the top of the card. Here, you can also find a link-out to the detailed REEV documentation on ACMG criteria.

Finally, you can save your ACMG rating, load a preexisting one, or delete it from the server.

2.5.3 InterVar Automation

Explaining the ACMG criteria for sequence variants is beyond the scope of this manual, but we provide a brief overview of the InterVar[Page 33, 1] automation rules.

Table 1: Sequence variant ACMG criteria as implemented in InterVar.

Criteria	Summary
PVS1	Null variants on canonical transcript for 4807 identified LOF-intolerant gene list, before 50 nucleotides of final exon-junction complex
PVS1	Null variants on canonical transcript for 4807 identified LOF-intolerant gene list, before 50 nucleotides of final exon-junction complex
PS1	Automatic match against list of ClinVar pathogenic missense-variants, same AA change
PS4	Variants with OR > 5.0 in GWASdb v2
PM1	domain info from dbnsfp31a_interpro database, list of domains with only pathogenic and likely pathogenic variants based on ClinVar data
PM2	absent in ESP6500, 1000 Genomes, ExAC for dominant or AAF <0,5% for recessive
PM4	non-frameshift insertion/deletion, stop-loss in non-repeat regions (rmsk database UCSC browser)
PM5	Automatic match against list of ClinVar pathogenic missense-variants, different AA change
PP2	>80% pathogenic (at least one) clinvar variants missense and <10% benign (and less than one)
PP3	dfnsfp30a MetaSVM (>0, deleteriousness), GERP++ (>2.0, conservation), dbscnv11 (>0.6 ADA, RF scores)
PP5	ClinVar or HGMD as database
BA1	AAF >5%
BS1	AAF >1% (default cutoff, user-adjustable)
BS2	hom (for AR) or het (for AD) in 1000 Genomes
BP1	>80% pathogenic (at least one) clinvar variants truncating
BP3	non-frameshift insertion, non-frameshift deletion in repeat region (defined by rmsk database)
BP4	Evidence (see PP3) does not suggest impact
BP6	ClinVar or HGMD as database
BP7	dbscnv RF and ADA <0.6, GERP++ <2 (not conserved)

The following criteria are not implemented in InterVar:

Table 2: Sequence variant ACMG criteria not implemented in InterVar.

Criteria	Summary
PS2, PM6	de novo status of variant
PS3, BS6	functional studies
PM3, BP2	variant in cis/trans with known pathogenic
PP1, BS4	familial segregation
PP4	phenotype and family history
BP5	alternative molecular basis

Consequences

This cards shows the consequences of your variant of interest by providing information on

- the gene affected
- the different transcript variants (RefSeq)
- the respective consequence as type of the variant (missense, nonsense, frameshift, splice, etc.) and the change on cDNA an protein level in the corresponding transcript
- which of how many exons is affected by this variant in the corresponding transcript

ClinVar

This card shows information on the variant available in the ClinVar database such as the annotated interpretation (benign, likely benign, uncertain significance, likely pathogenic, pathogenic), the evidence level / review status (1 to 5 stars) and a link-out to this entry in ClinVar. Using the button in the lower right corner of the card you can expand this ClinVar information, e.g. to the associated condition linked to this variant.

Scores

This card shows a variety of precomputed sequence variant scores, e.g. CADD, PolyPhen2, SIFT, REVEL etc. Note that REEV also provides the precomputed splice predictions scores MMSplice and SpliceAI for the assessment of potential splice site variants. By default the most pathogenic prediction is displayed. Using the button next to the tool on the left you can also expand both predictions to all the different splice site change scores calculated (e.g. acceptor-gain, acceptor-loss, donor-gain, . . .).

REEV provides not only the raw scores but also an interpretation of the respective score. To this end, a color visualization of each score is shown with green color indicating a benign and red color indicating a pathogenic prediction. On the right REEV provides you with the respective evidence level you can use on the ACMG PP3 criterion according to recent ClinGen recommendations by Pejaver *et al.*[3] who advise that “For missense variants, to determine evidence for codes PP3 and BP4, we recommend that, for most situations, clinical laboratories use a single tool, genome-wide, that can reach the strong level of evidence for pathogenicity and moderate for benignity (BayesDel, MutPred2, REVEL, or VEST4)” The interpretation given by REEV follows the respective thresholds for each score published in these guidelines (Table 2).

On the bottom of this card you can also find information on conservation (UCSC 100 vertebrate vonservation) for the position affected by the variant of interest.

Note, that if there are more than one transcript variant for gene, on the bottom right of this card you can choose and switch between the different transcript variants and the corresponding predictions for this respective transcript variant will be provided above.

[3] V. Pejaver, A. B. Byrne, B. J. Feng, K. A. Pagel, S. D. Mooney, R. Karchin, A. O’Donnell-Luria, S. M. Harrison, S. V. Tavtigian, M. S. Greenblatt, L. G. Biesecker, P. Radivojac, S. E. Brenner, L. G. Biesecker, S. M. Harrison, A. A. Tayoun, J. S. Berg, S. E. Brenner, G. R. Cutting, S. Ellard, M. S. Greenblatt, P. Kang, I. Karbassi, R. Karchin, J. Mester, A. O’Donnell-Luria, T. Pesaran, S. E. Plon, H. L. Rehm, N. T. Strande, S. V. Tavtigian, and S. Topper. Calibration of computational tools for missense variant pathogenicity classification and ClinGen recommendations for PP3/BP4 criteria. *Am J Hum Genet*, 109(12):2163–2177, Dec 2022.

Population Frequencies

This card shows the variant's occurence in the control database gnomAD and also provides the respective link-out to gnomAD.

Variant Tools

This card provides useful further link-outs on variant level to the genome browesers ENSEMBL and UCSC as well as to further resources such as MutationTaster, Varsome, etc. By clicking on the "Jump in local IGV" button on the bottom, you can also look at the variant in IGV when you have the respective bam-file opened in your local IGV.

Beacon Network

In this card you can connect to others users via the beacon network to search for your variant of interest and associated information at other sites.

Variant Validator

In this last card you can submit the variant to VariantValidator to obtain gold standard HGVS description to make short to report the variant correctly in your lab report or paper.

2.5.4 Structural Variants

Gene List

In this first card you find an overview in the form of a gene list of all genes overlapping and contained in the region affected by you structural variant of interest. In this overview you find the Gene symbol, RefSeq MANE transcript ID as well as important scores on haploinsufficiency and triplosensitivity of the respective gene (see also *Genes*) For the currently selected gene, the information described in the section *Genes* is displayed.

Note, that if you investigate a larger SV affecting multiple genes, REEV can help you prioritize that larger set of genes by sorting by different criteria using the "sort by" selection box on the upper right corner of the card to sort the gene list by different (e.g. haploinsufficiency or triplosensitivity) scores.

ClinVar

This card shows information on overlapping variants listed in the ClinVar database, their annotated interpretation (benign, likely benign, uncertain significance, likely pathogenic, pathogenic), the evidence level / review status (1 to 5 stars), the associated condition linked to this variant and the size of the respective overlap of this ClinVar variant with your SV of interest. Note, that you can expand every row to show more information on the respective ClinVar variant and that you can sort the list of ClinVar variants by size of their overlap to your SV.

Variant Tools

This card provides useful further link-outs on variant level to the genome browesers ENSEMBL and UCSC as well as to further resources such as MutationTaster, Varsome, etc. By clicking on the "Jump in local IGV" button on the bottom, you can also look at the variant in IGV when you have the respective bam-file opened in your local IGV.

Clinical Significance

This card shows the semi-automated pathogenicity prediction based on AutoCNV[4]. Using the buttons to the left of each criterion you can select or deselect every ACMG CNV criterion[5]. The semi-automated prediction is providing an automated scoring for criteria 1-3 while you always have to select criteria 4 and 5 manually based on your clinical information on the case. On default you will see the automated selection of ACMG criteria. You can individually select and deselect every ACMG CNV criterion using the little switch displayed left to every criterion and also select the individual points you score on this criterion.

To help you with your manual ACMG assessment, you find a description next to every ACMG CNV criterion and a summary of the points suggested to use for this criterion as well as the maximum score allowed for this criterion.

Note, that you can also reset all selected ACMG criteria to auto using the respective button below the pathogenicity prediction box on the top of the card. Here, you can also find a link-out to the detailed REEV documentation on ACMG criteria.

2.5.5 AutoCNV Automation

Explaining the ACMG criteria for copy number variants is beyond the scope of this manual, but we provide a brief overview of the AutoCNV[Page 37, 4] automation. The following criteria are implemented.

[4] C. Fan, Z. Wang, Y. Sun, J. Sun, X. Liu, L. Kang, Y. Xu, M. Yang, W. Dai, L. Song, X. Wei, J. Xiang, H. Huang, M. Zhou, F. Zeng, L. Huang, Z. Xu, and Z. Peng. AutoCNV: a semiautomatic CNV interpretation system based on the 2019 ACMG/ClinGen Technical Standards for CNVs. *BMC Genomics*, 22(1):721, Oct 2021.

[5] E. R. Riggs, E. F. Andersen, A. M. Cherry, S. Kantarci, H. Kearney, A. Patel, G. Raca, D. I. Ritter, S. T. South, E. C. Thorland, D. Pineda-Alvarez, S. Aradhya, and C. L. Martin. Technical standards for the interpretation and reporting of constitutional copy-number variants: a joint consensus recommendation of the American College of Medical Genetics and Genomics (ACMG) and the Clinical Genome Resource (ClinGen). *Genet Med*, 22(2):245–257, Feb 2020.

Table 3: Copy number loss variant ACMG criteria as implemented in AutoCNV.

Section	Rule	Summary
1	1A	0 otherwise
	1B	-0.6 if no protein coding genes or functionally important elements
2	2A	1 if del spans haploinssuficient or dup spans triplosensitive gene or region (clingen database)
	2B	0 no overlap
	2C	del: 0.9 if exon involved or 0.0 without for partial 5' overlap; dup: -1 if same gene content as known benign CNV
	2D	del: 0.9 for 3' overlap if pathogenic variants documented in exon (P/LP ClinVar with AF <1% gnomAD), 0.3 without known pathogenic, 0.9 multiple exons; dup: -1 smaller than benign CNV
	2E	del: AutoPVS1, 0.9 if NMD, 0.45 if altered region critical, 0.45 if $> 10\%$ protein removed, 0.3 if $< 10\%$ protein; dup: assign 0 if interrupts protein coding gene
	2F	del: -1 if established benign genes, regions; dup: $[-1, 0]$ if larger than benign duplication without additional coding genes
	2G	0 if established benign genes but includes additional regions
	2H	0.15 gene pLI ≥ 0.9 and Decipher HI $\leq 10\%$
	2I	dup: AutoPVS1, 0.9 if tandem + NMD, 0.45 if tandem
	2L	dup: 0 genes without clinical significance
3	3A	0 otherwise
	3B	0.45 if del $[25..34]$ or dup $[35..49]$ protein coding genes
	3C	0.9 for del > 35 and dup > 50 protein coding genes
4	4O	-1 if CNV entirely within common variation (DGV Freq $\geq 1\%$ or gnomAD $\geq 1\%$), or if overlap $> 50\%$ without containing other protein coding genes

The following criteria are not implemented in AutoCNV.

Table 4: Copy number loss variant ACMG criteria not implemented in AutoCNV.

Section	Rule	Summary
2	2J, 2K	patient phenotype consistency with LoF of gene
4	4A-N	phenotype, segregation in literature, case/control
5	5A-5H	patient phenotype, family segregation

Genome Browser

This card provides an internal genome browser with useful tracks for interpreting the variant. You see the genomic location of the variant along with useful tracks from UCSC (e.g. Repeat Masker), RefSeq Genes as well as gnomAD and DGV SVs, ExAC CNVs.

2.6 ClinVar Submission

This section describes the ClinVar submission feature of REEV.

2.6.1 Prerequisites

To use this feature, you will need to fulfill the following prerequisites.

1. You must have a ClinVar API key. For this, you will first need to register your group with ClinVar and then request API access via an email to clinvar@ncbi.nlm.nih.gov. You can find out more information here about registering your organization with ClinVar and here about API keys.

2. You need a REEV account. This is easy, just click on "Login" on the top right of the REEV website. The account will be enabled after logging in with one of the following options.

   As a member of a research organisation in the European Union, you can login via your home organisation using LifeScience Research Infrastructure (LSRI). Otherwise, or if you prepare, you can login with OrcID. OrcID is a free service that allows you to create a unique identifier for your publications, and you can also create an OrcID account by connecting your Google/Microsoft/… account.

   We chose these two options because we do not want to handle and store passwords and both LSRI/OrcID are secure login options and available to us without charge.

Please note that the REEV authors are not affiliated with ClinVar, NCBI, or NIH.

2.6.2 Overview

Now that you have an account with REEV, access to a group account at ClinVar and your ClinVar API key (64 character long token), we can get started. The overall process will be as follows:

As a one-time setup, you will store the ClinVar API key in your REEV account. Note that multiple people from the same group can do this and everything will still work.

The process is then as follows:

1. You enter the sequence or structural variant that you want to upload in to REEV and go to the page.

2. You go to the "ClinVar Submission" tab and create a new submission.

3. You send a create, update, or delete request to ClinVar by:

   a. Enter the required information (e.g., SCV accession for a deletion, or variant information for creation).

   b. Store this request on the REEV server which will put it into the work queue of REEV. REEV will shortly submit this request to ClinVar.

   c. ClinVar will put your request into their work queue and process it eventually. Note that this takes some time (in our experience 2 hours are not rare). During this process, ClinVar will perform validation steps and may reject your request.

      The earlier that you get feedback from ClinVar, the higher the chance that there is a problem, so be patient. Also see the section *Common Errors*. After all automated validation steps are complete, you will get an SCV identifier and your submission will be published at the next release (usually the next Sunday). No human validation is done on the side of ClinVar.

      Compared to the Excel submission, this process is a huge improvement even if it sounds complicated.

2.6.3 ClinVar API Keys in Profile

After logging in, click "Profile" on the top right of the REEV website. Then, select "Orgs / Keys" menu on the left below "ClinVar Submissions". You will be shown the following screen. Here, you can find all of your organisations for which you have stored an API key in REEV. Most users, will not have more than one entry here.

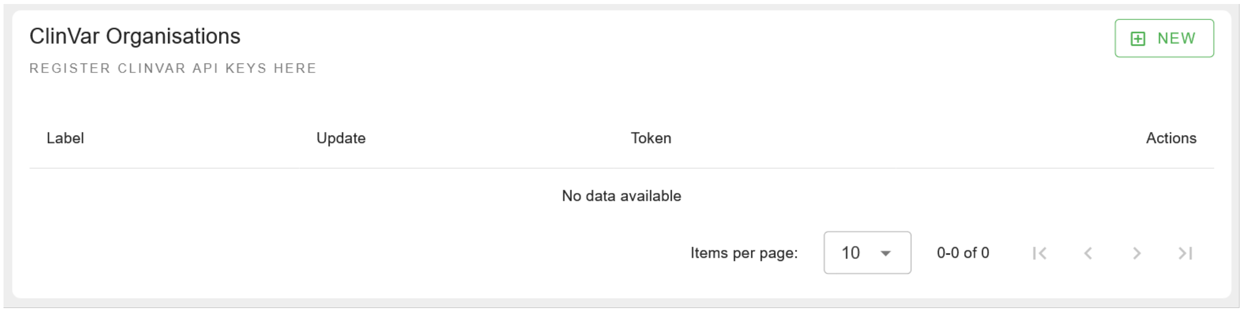

To create a new entry, click on the green "New" button on the top right. A modal will be shown where you can register a new organisation. You have to provide a label (only used for display purposes) and the ClinVar API key. Click "Create" to store the new entry.

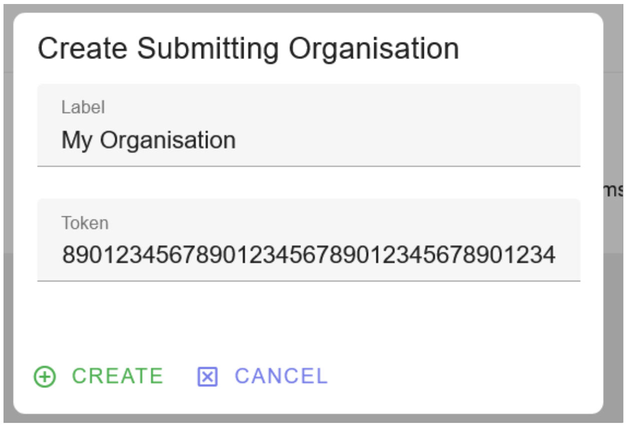

You now have successfully registered your ClinVar API key and can start create submissions.

2.6.4 Submit Novel Record

Submitting a *novel* record means that a given variant has not been submitted to ClinVar before. In this case, simply navigate to the given sequence or structural variant in REEV. Click "ClinVar Submission" in the navigation or scroll down to the corresponding box.

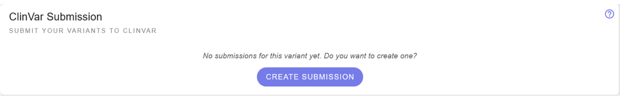

Here, click the "Create Submission" button. A step by step wizard will guide you through the process. First, select the organisation for which you want to submit the record. The screenshots below show the prcoess for sequence variants but the process is the same for structural variants. Here, we focus on new submissions, in *Variant Update* we will discuss updates and in *Variant Deletion* we will discuss deletions. Click "Next".

On the next screen, you can enter the information that you want to submit. The most important fields are the clinical significance and the condition. Also, you might want to assign some HPO phenotype terms of your patient. When you are done, click "Next".

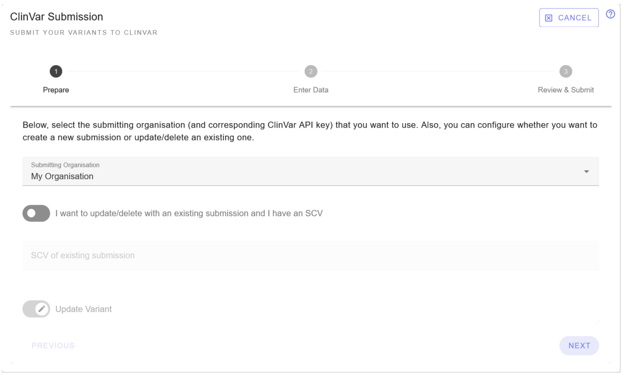
ClinVar Submission
SUBMIT YOUR VARIANTS TO CLINVAR
CANCEL
Prepare
Enter Data
Review & Submit
Below, select the submitting organisation (and corresponding ClinVar API key) that you want to use. Also, you can configure whether you want to create a new submission or update/delete an existing one.
Submitting Organisation
My Organisation
I want to update/delete with an existing submission and I have an SCV
SCV of existing submission
Update Variant
PREVIOUS
NEXT

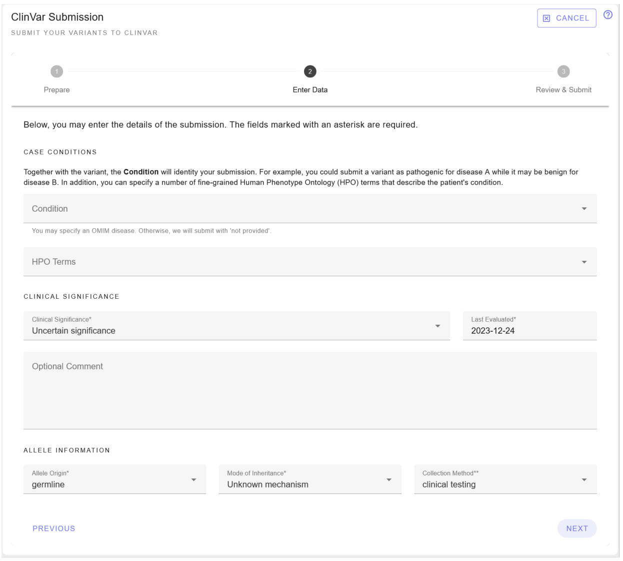
ClinVar Submission
SUBMIT YOUR VARIANTS TO CLINVAR
CANCEL
Prepare
Enter Data
Review & Submit
Below, you may enter the details of the submission. The fields marked with an asterisk are required.
CASE CONDITIONS
Together with the variant, the Condition will identity your submission. For example, you could submit a variant as pathogenic for disease A while it may be benign for disease B. In addition, you can specify a number of fine-grained Human Phenotype Ontology (HPO) terms that describe the patient's condition.
Condition
You may specify an OMIM disease. Otherwise, we will submit with 'not provided'.
HPO Terms
CLINICAL SIGNIFICANCE
Clinical Significance*
Uncertain significance
Last Evaluated*
2023-12-24
Optional Comment
ALLELE INFORMATION
Allele Origin*
germline
Mode of Inheritance*
Unknown mechanism
Collection Method**
clinical testing
PREVIOUS
NEXT

In this screen, you can review your submission. In the case that you want to adjust something, click "previous" to go back. Otherwise, click "submit creation request" to start the submission. By this, you create a submission job in REEV and a request will be sent to ClinVar.

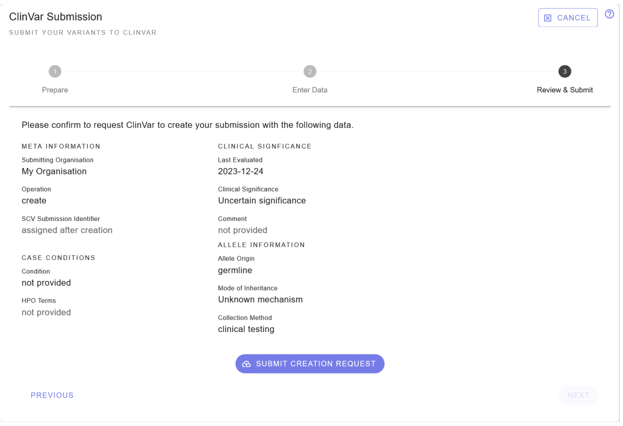

This job will be shown in the following overview table. It can take some time for ClinVar to process your request, and updates will be shown in the table below.

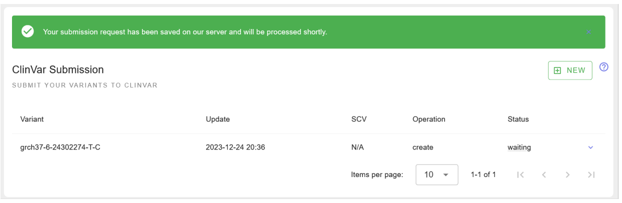

You can also go to your profile and the click "Submission Activity" on the left. You can see all of your past and current submission activity.

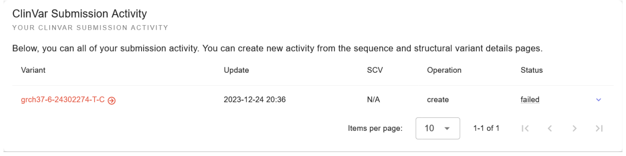

2.6.5 Variant Update

Variant updates work the same as variant creations. However, in the first step you have to enable "I want to update/delete an existing submission and I have an SCV", enter the SCV accession, and keep the third switch as "Update Variant". Note that you will currently have to re-fill the data in the "Enter Data" step.

2.6.6 Variant Deletion

Deleting a submission is even simpler. In the first step, check "I want to update/delete an existing submission and I have an SCV", enter the SCV accession, and change the third switch to "Delete Variant". You can optionally provide a free-text reason for removing the variant submission.

2.6.7 Common Errors

Invalid API Key

Message
No valid API key provided.

Explanation
The API key that you provided is not valid. Please check that you have entered the correct key.

Resolution
Correct the value that you provided in the REEV profile.

Record is not Novel

Message
This record is submitted as novel but it should be submitted as an update, including the SCV accession, because your organization previously submitted <SCV> for the same variant and condition.

Explanation
Your organisation has already submitted a record with this "name" and condition (OMIM code or "not provided"). ClinVar generates a variant name from your genomic coordinates. Each organisation can only have one submission for the combination of the condition and variant.

Resolution
You can either submit a revision of your interpretation, (or, e.g., extend the "observed in" information), or leave the record as it is.

Submission Names Cannot be Changed

Message
This update changes the description of the variant for <SCV>, which is generally not allowed on a ClinVar record. Please check the description of the variant and correct if necessary. If you intend to change the description of the variant, please submit as a new record and delete this record. Contact clinvar@ncbi.nlm.nih.gov if you have questions.

Explanation
Most likely, you try to update the coordinates of a variant with an existing SCV. ClinVar does not allow this. Rather, you should remove the old variant and create a new submission.

Resolution
Remove the old variant and add a new variant instead.

Multiple Conditions have been submitted without explanation

Message
You provided multiple diseases as the condition for the classification. If they represent related diseases along a spectrum, provide uncertain for multipleConditionExplanation. If they represent diseases that occur together in an individual with the variant (this case is rare), provide co-occurring for multipleConditionExplanation."

Explanation
Multiple Condition IDs have been submitted for single variant. Check if this has been intentional. If multiple conditions are to be submitted, a reason needs to be included with the submission.

Resolution
Explicitly add either Uncertain, Co-occurring or Novel disease to the list of CONDITIONS.

2.7 Dev Quickstart

Developer or User?

This section is part of the instrutions for programmers interested in REEV. If you want to **use** REEV, the best place is to start at *Quickstart*.

This section explains how to get started when you want to do REEV software development. Probably, it is best to contact us via email to cubi@bih-charite.de or open a discussion in the GitHub repository. However, REEV is fully open source, so here is how to get started hacking away - also on your own.

2.7.1 Overview

You will need to have Python 3.10, pipenv, and Node.js 20.x installed. The next step is to checkout the repository and install the Python/Node dependencies. Then, you will be able to run the frontend and backend servers. The following assumes a Debian/Ubuntu machine; your mileage may vary.

2.7.2 Prerequisites

You can use pyenv for getting a specific python version.

```
$ sudo apt-get update; sudo apt-get install make build-essential libssl-dev zlib1g-dev \
libbz2-dev libreadline-dev libsqlite3-dev wget curl llvm \
libncursesw5-dev xz-utils tk-dev libxml2-dev libxmlsec1-dev libffi-dev liblzma-dev
$ curl https://pyenv.run | bash
```

Append the following to your `` `~/.bashrc ``:

```
$ pyenv
export PATH="$HOME/.pyenv/bin:$PATH"
eval "$(pyenv init --path)"
eval "$(pyenv virtualenv-init -)"
```

... and ensure to execute/source this as well (`exec $SHELL`).

Now you can install a specific python version:

```
$ pyenv install 3.10
$ pyenv local 3.10
```

Install pipenv:

```
$ pip install --user pipenv
```

Install NodeJS v20 (LTS):

```
$ curl -fsSL https://deb.nodesource.com/setup_20.x | sudo -E bash -
$ sudo apt-get install -y nodejs
```

2.7.3 Clone Repository

```
$ git clone --recursive git@github.com/bihealth/reev.git
```

2.7.4 Install Dependencies

You can use the provided `Makefile` files to install the dependencies.

```
$ make deps
```

2.7.5 Setup Environment

You need to create an `.env` file for the backend. The values in `env-dev` are suitable for development with the `reev-docker-compose` with `docker-compose.override.yml-dev`.

```
$ ln -sr backend/env-dev backend/.env
```

2.7.6 Database Migrations

Next, you will need to run the database migrations.

```
$ make -C backend alembic-upgrade
```

2.7.7 Running the Servers

For development, you have to run a server both for the frontend and the backend. In deployment, the frontent will be built to a static `dist` directory and served through the backend HTTP server.

Execute the following commands in separate terminals. The servers and celery will be started with automated code reload. In case of weird issues, try to stop them with `Ctrl-C` and starting them again. You have to restart jupyterlab manually.

```
$ make -C backend serve
$ make -C backend celery
$ make -C backend jupyterlab
$ make -C frontend serve
```

Now you can navigate to the frontend development server at http://localhost:8081. This server will transparently forward the API requests to the backend server at http://localhost:8081.

2.7.8 Troubleshooting

Issues with nested git submodules.
We have put the shared code into `frontend/src/ext/reev-frontend-lib`. Make sure that you get a fresh clone of the repository with the `--recursive` flag. To initialize the submodules, use `git submodule update --init --recursive`. You can reset the submodule to the version stored in the main `reev` repository with `git submodule foreach --recursive git reset --hard`.

Issues with `reev-frontend-lib` as a submodule.
Having the library as a submodule is useful for development. There are some drawbacks. If you see **weird issues with typescript that you don't understand**, try to `rm -rf frontend/src/ext/reev-frontend-lib/{node_modules,dist}`. Such issues include problems with assigning the reactive classes from `vue`, such as `ref` and `computed`, or other weird stuff with types. In this case, try to clear out the directories described above and restart VS Code.

2.7.9 Notes

- A superuser will be created if you configured its email and password in environment variables `FIRST_USER_EMAIL` and `FIRST_USER_PASSWORD`.

2.8 Helper Makefiles

Developer or User?

This section is part of the instrutions for programmers interested in REEV. If you want to **use** REEV, the best place is to start at *Quickstart*.

We created some `Makefile` files to help with common tasks. Call `make help` to see them. You can call them from the top-level directory or as `make -C backend` or `make -C frontend`.

2.9 Frontend Development

Developer or User?

This section is part of the instrutions for programmers interested in REEV. If you want to **use** REEV, the best place is to start at *Quickstart*.

This section describes the best practices to use for frontend development.

2.9.1 Import Order

Import order should be (and is also enforced by prettier):

- external packages
- project includes with `@/` prefix
- relative includes with `./` prefix

Overall, restrict relative include order for tests to include code to be tested with `../`.

2.9.2 Types

- For the Vue reactive stores, explicitely specify the type in the creating function, e.g., *ref<string>* or *ref<string | undefined>*.
- In many places in the UI, using *undefined* is more appropriate than *null* as this is the value before the data is loaded from the backend.

2.9.3 Test Structure

Consider the following structure, an example is given below.

- imports
- **define fixture data**
  - put larger fixture data into `.json` files within the `__tests__` folders (will go to LFS by our `.gitattributes` configuration)
- define the tests
- **use assemble, act, assert structure e.g., as described here**
  - guard assertions are OK
- use `describe.concurrent` to describe the tests, usually one block per `.spec.ts` file
- **use `it` to define the tests**
  - use `async () => { ... }` only when necessary, e.g., for `await nextTick()`
- use the `setupMountedComponents()` helper from `@/components/__tests__/utils` to mount components, setup store, and setup router with mocks

```
import { setupMountedComponents } from '@bihealth/reev-frontend-lib/lib/testUtils'
import { describe, expect, it } from 'vitest'
import { h, nextTick } from 'vue'

import { type UserData, useUserStore } from '@/stores/user'

import UserProfileButton from './UserProfileButton.vue'

/** Example User data */
const adminUser: UserData = {
  id: '2c0a153e-5e8c-11ee-8c99-0242ac120002',
  email: 'admin@example.com',
  is_active: true,
```

(continues on next page)

(continued from previous page)

```
  is_superuser: true,
  is_verified: true,
  oauth_accounts: [
    {
      id: '2c0a153e-5e8c-11ee-8c99-0242ac120002',
      oauth_name: 'google',
      account_id: '1234567890',
      account_email: 'admin@example.com'
    }
  ]
}

/** Dummy routes for testing. */
const dummyRoutes = [
  {
    path: '/',
    name: 'home',
    component: h('div', { innerHTML: 'for testing' })
  },
  {
    path: '/login',
    name: 'login',
    component: h('div', { innerHTML: 'for testing' })
  },
  {
    path: '/profile',
    name: 'profile',
    component: h('div', { innerHTML: 'for testing' })
  }
]

describe.concurrent('UserProfileButton', () => {
  it('displays Login button without any user', async () => {
    // arrange:
    const { wrapper } = await setupMountedComponents(
      { component: UserProfileButton },
      {
        initialStoreState: {
          user: {
            currentUser: null
          }
        },
        routes: dummyRoutes
      }
    )

    // act: nothing, only test rendering

    // assert:
    const loginButton = wrapper.findComponent('#login')
    expect(loginButton.exists()).toBe(true)
    const logoutButton = wrapper.findComponent('#profile')
```

(continues on next page)

(continued from previous page)

```
    expect(logoutButton.exists()).toBe(false)
  })

  it('displays Profile button with a user', async () => {
    // arrange:
    const { wrapper } = await setupMountedComponents(
      { component: UserProfileButton },
      {
        initialStoreState: {
          user: {
            currentUser: adminUser
          }
        },
        routes: dummyRoutes
      }
    )

    // act: nothing, only test rendering

    // assert:
    const loginButton = wrapper.findComponent('#login')
    expect(loginButton.exists()).toBe(false)
    const logoutButton = wrapper.findComponent('#profile')
    expect(logoutButton.exists()).toBe(true)
  })

  it('switches from Login to Profile button when store changes', async () => {
    // arrange:
    // Note that we use an `async` test here as we need `await nextTick()` for the DOM
    // update to bubble through when updating the state property.
    const { wrapper } = await setupMountedComponents(
      { component: UserProfileButton },
      {
        initialStoreState: {
          user: {
            currentUser: null
          }
        },
        routes: dummyRoutes
      }
    )

    // act:
    let loginButton = wrapper.findComponent('#login')
    expect(loginButton.exists()).toBe(true)
    let logoutButton = wrapper.findComponent('#profile')
    expect(logoutButton.exists()).toBe(false)

    const userStore = useUserStore()
    userStore.currentUser = adminUser

    await nextTick()
```

(continues on next page)

(continued from previous page)

```
    // assert:
    loginButton = wrapper.findComponent('#login')
    expect(loginButton.exists()).toBe(false)
    logoutButton = wrapper.findComponent('#profile')
    expect(logoutButton.exists()).toBe(true)
  })
})
```

Note that there is a separation between views and components (cf. https://stackoverflow.com/a/50866150/84349) that is also reflected in the tests.

- The main purpose of views is to handle the routing.
- The actual work happens in components.

In tests, we thus use stubbed out of nested components in views but not components. This keeps the load time acceptable while testing the components in isolation.

2.10 Backend Development

Developer or User?

This section is part of the instrutions for programmers interested in REEV. If you want to **use** REEV, the best place is to start at *Quickstart*.

2.10.1 Types

- prefer to use semantic-carrying types, e.g., `enum.Enum` over `bool` or `int`

2.10.2 Test Structure

- use assemble, act, assert structure e.g., as described here
- properly use fixtures, place them in `conftest.py` files
- **use `pytest.mark.parameterize` to condense multiple test cases into one tests but don't overdo it**
  - see `test_clinvarsub.py` for an example (e.g., `test_create_submissionthreads`) that is still good a trade-off but probably shows the highest degree of complexity (read: different test code paths)

2.11 Continuous Integration

Developer or User?

This section is part of the instrutions for programmers interested in REEV. If you want to **use** REEV, the best place is to start at *Quickstart*.

We make extensive use of GitHub actions for continuous integration:

- `automerge.yml` will automatically accept merge requests from dependabot if when CI passes
- `conventional-prs.yml` will enforce that all PRs have a title that follows the conventional commit format
- `docker-build.yml` will automatically build Docker images for the `main` branch and all PRs
- `docker-cleanup-pr.yml` cleans up PR Docker images after they are merged
- `docker-cleanup-untagged.yml` cleans up untagged Docker images
- `main.yml` runs lintings and testing for the frontend and backend code and uploads coverage info to Coverage.io
- `release-please.yml` will automatically manage release PRs and releases

2.11.1 Conventional Commits

We follow conventional commits and enforce merge requests. We use release-please for managing our releases and changelog. For best practice this comes down to the following.

1. Open a proper issue for your change and document it as a "bug fix" or "enhancement". Alternatively, you can also create an *ad-hoc* commit but it is better to document the motivation, desired action, and actual implementation of the change.
2. Create a branch with a name that corresponds to the desired change/issue. GitHub has documentation for how to do this from an issue.
3. Create appropriate pull request form the branch. The first issue should probably follow the conventional commits format (for examples, see below). This project has been setup that branches can only be merged with squash pull requests. In the case of only one commit in a PR, the first line of the only commit will be used (for technical reasons). Otherwise, it would be sufficient for the PR to have a semantic commit message.
4. Get the PR reviewed.
5. Merge the PR.

Examples for commmit messages:

```
chore: a minor change that does not get an entry in the changelog
fix: a fix that will enforce bumping the patch component of the version
feat: a feature that will enforce bumping the minor component of the version
fix!: a fix with backwards change that will enforce bumping the major component
feat!: similar as for fix! but for features
ci: changes to the CI that does not appear in the changelog
build: changes to the build system that does not appear in the changelog
docs: changes to the documentation that does not appear in the changelog
```

You can use a `Release-As:` footer to enforce things being upgraded or downgraded in the changelog.

2.12 Deployment

Developer or User?

This section is part of the instrutions for programmers interested in REEV. If you want to **use** REEV, the best place is to start at *Quickstart*.

We deploy with Docker images. These are built automatically via GitHub CI and publisheed to the GitHub Container Registry (`ghcr.io`).

To build the images locally, run:

```
$ bash utils/docker/build-docker.sh
```

If you look at the `build-docker.sh` file then you will notice that you can pass additional parameters. A useful one is `--no-cache` that will disable the Docker build cache.

```
$ bash utils/docker/build-docker.sh --no-cache
```

You can run the Docker image on port 8080 as follows (make sure that you replace `0.0.0` with the version built previously):

```
$ docker run --name reev --rm -p 8080:8080 ghcr.io/bihealth/reev:0.0.0
```

To attach to the container and look around:

```
$ docker run -it reev bash -i
```

2.13 Writing Documentation

Developer or User?

This section is part of the instrutions for programmers interested in REEV. If you want to **use** REEV, the best place is to start at *Quickstart*.

Documentation is maintained in the `docs/` directory using Sphinx. It is automatically built to reev.readthedocs.io.

2.14 Tests

Developer or User?

This section is part of the instrutions for programmers interested in REEV. If you want to **use** REEV, the best place is to start at *Quickstart*.

Tests can be run with `make test` (or `make -C backend/frontend test`).

We use Pytest for testing the backend and Vitest for the frontend.

2.15 GitHub Project Management

Developer or User?

This section is part of the instrutions for programmers interested in REEV. If you want to **use** REEV, the best place is to start at *Quickstart*.

We use Terraform for managing the GitHub project settings (as applicable):

```
$ export GITHUB_OWNER=bihealth
$ export GITHUB_TOKEN=ghp_<thetoken>

$ cd utils/terraform
$ terraform init
$ terraform import github_repository.reev reev
$ terraform validate
$ terraform fmt
$ terraform plan
$ terraform apply
```

2.16 Operator Introduction

This section gives you an overview of REEV operation. Overall, a REEV installation consists of a number of Docker containers with access to static data.

We currently run the containers using Docker Compose. Data is hosted on our S3 servers. Read the section *Deployment Instructions* for information on getting both software and data.

You can find the source code of everything in the following repositories:

REEV Web Application
: https://github.com/bihealth/reev

REEV Frontend Library with shared code
: https://github.com/bihealth/reev-frontend-lib

REEV Docker Compose
: https://github.com/bihealth/reev-docker-compose

Data Downloader
: https://github.com/varfish-org/varfish-db-downloader

This Snakemake workflow downloads and processes the data. You do not need to do this but can use the data provided on our S3 servers. See *Deployment Instructions* for more details.

2.17 Deployment Instructions

This section describes the deployment of REEV, both for development and production.

2.17.1 Prerequisites

You will need to fetch some of this from our S3 server. We recommend the `s5cmd` tool as it is easy to install, use, and fast. You can download it from github.com/peak/s5cmd/releases. For example:

```
wget -O /tmp/s5cmd_2.1.0_Linux-64bit.tar.gz \
    https://github.com/peak/s5cmd/releases/download/v2.1.0/s5cmd_2.1.0_Linux-64bit.tar.gz
tar -C /tmp -xf /tmp/s5cmd_2.1.0_Linux-64bit.tar.gz
sudo cp /tmp/s5cmd /usr/local/bin/
```

You will need to install Docker Compose. Note that the "modern" way is to do this by using the docker compose plugin. Instructions can be found here on the Docker.com website.

2.17.2 Checkout and Configure

First, clone the repository:

```
git clone git@github.com:bihealth/reev-docker-compose.git
```

From here on, the commands should be executed from within this repository (`cd reev-docker-compose`).

We will use the directory `.dev` within the checkout for storing data and secrets. In a production deployment, these directories should live outside of the checkout, of course.

Now, we create the directories for data storage.

```
mkdir -p .dev/volumes/pgadmin/data
mkdir -p .dev/volumes/postgres/data
mkdir -p .dev/volumes/rabbitmq/data
mkdir -p .dev/volumes/redis/data
mkdir -p .dev/volumes/reev-static/data
```

Next, we setup some "secrets" for the passwords.

```
mkdir -p .dev/secrets
echo db-password >.dev/secrets/db-password
echo pgadmin-password >.dev/secrets/pgadmin-password
```

We now copy the `env.tpl` file to the default location for the environment `.env`.

```
cp env.tpl .env
```

Next, create a `docker-compose.override.yml` with the contents of the file `docker-compose.override.yml-dev`. This will disable everything that we assume is running on your host when you are developing. This includes the REEV backend, rabbitmq, celery workers, postgres.

```
cp docker-compose.override.yml-dev docker-compose.override.yml
```

2.17.3 Download Data

To serve data via the mehari, viguno, and annonars containers, you need to obtain the required datasets. We have prepared significantly reduced datasets (totaling less than 2GB as opposed to hundreds of GB) for development purposes.

We provide a script that sets up the necessary directories, downloads the data, and creates symlinks.

By default, the script verifies SSL certificates when downloading data. If you encounter SSL verification issues or operate in an environment where SSL verification is not required, you can disable SSL verification by setting the `NO_VERIFY_SSL` variable to 1 when running the script.

To download the data with SSL verification (default behavior):

```
bash download-data.sh
```

Note: Note that you can also download the full data by using `DOWNLOAD=full bash download-data.sh` below. To use a reduced dataset to exons plus/minus 100bp, use `DOWNLOAD=reduced-exomes bash download-data.sh`.

To download the data without SSL verification:

```
NO_VERIFY_SSL=1 bash download-data.sh
```

Note: Disabling SSL verification can make the connection less secure. Use this option only if you understand the risks and it is necessary for your environment.

2.17.4 Setup Configuration

The next step step is to create the configuration files in `.dev/config`.

```
mkdir -p .dev/config/nginx
cp utils/nginx/nginx.conf .dev/config/nginx

mkdir -p .dev/config/pgadmin
cp utils/pgadmin/servers.json .dev/config/pgadmin
```

2.17.5 Startup and Check

Now, you can bring up the docker compose environment (stop with `Ctrl+C`).

```
docker compose up
```

To verify the results, have a look at the following URLs. These URLs are used by the REEV application.

- Annonars database infos: http://127.0.0.1:3001/annos/db-info?genome_release=grch37
- Annonars gene info: http://0.0.0.0:3001/genes/info?hgnc_id=HGNC:12403
- Annonars variant info: http://0.0.0.0:3001/annos/variant?genome_release=grch37&chromosome=17&pos=41244100&reference=G&alternative=A
- Mehari impact prections: http://127.0.0.1:3002/tx/csq?genome-release=grch37&chromosome=17&position=48275363&reference=C&alternative=A
- Viguno for TGDS: http://127.0.0.1:3003/hpo/genes?gene_symbol=TGDS

- Nginx server with browser tracks http://127.0.0.1:3004/
- Dotty server with c./n./g. to SPDI resolution http://127.0.0.1:3005/api/v1/to-spdi?q=NM_000059.3:c.274G%3EA

Note that the development subset only has variants for a few genes, including BRCA1 (the example above).

You will also have the following services useful for introspection during development. For production, you probably don't want to expose them publically.

- flower, login is `admin`, with password `flower-password`
- pgAdmin for Postgres DB administration: http://127.0.0.1:3041 login is `admin@example.com` with password `pgadmin-password`

2.17.6 Service Information

This section describes the services that are started with this Docker Compose.

Traefik

Traefik is a reverse proxy that is used as the main entry point for all services behind HTTP(S). The software is well-documented by its creators. However, it is central to the setup and for much of the additional setup, touching Trafik configuration is needed. We thus summarize some important points here.

- Almost all configuration is done using labels on the `traefik` container itself or other containers.
- In the case of using configuration files, you will have to mount them from the host into the container.
- By default, we use "catch-all" configuration based on regular expressions on the host/domain name.

Dotty

Dotty (by the REEV authors) provides mapping from c./n./g. notation to SPDI.

Mehari

Mehari (by the REEV authors) provides information about variants and their effect on individual transcripts.

Viguno

Viguno (by the REEV authors) provides HPO/OMIM related information.

Annonars

Annonars (by the REEV authors) provides variant annotation from public databases.

Postgres

We use postgres for the database backend of REEV.

Rabbitmq

We use rabbitmq for message queues.

Redis

REDIS is used for storing authentication sessions.

PgAdmin

PgAdmin is a web-based administration tool for Postgres. We provide it for development and debugging but it can also come in handy in production.

Flower

Flower is a web-based application for monitoring and administrating Celery.

2.18 app.models

2.18.1 app.models.acmgseqvar

Models for ACMG sequence variant.

class `app.models.acmgseqvar.`**`AcmgSeqVar`**`(**kwargs)`

ACMG sequence variant.

`acmg_rank: AcmgRank`

ACMG criteria.

`id: Mapped[UUID]`

UUID of the ACMG sequence variant.

`seqvar_name: str`

Sequence variant ID.

`user: UUID`

User who created the ACMG sequence variant.

2.18.2 app.models.adminmsg

Models for admin messages.

class app.models.adminmsg.**AdminMessage**(***kwargs*)

Message to be set by administrators.

We currently only support read-only access, must be created via admin interface.

active_start: datetime

When to start displaying the message.

active_stop: datetime

When to stop displaying the message.

enabled

Whether the message is enabled at all.

id: Mapped[UUID]

UUID of the message.

text: str

The message's text.

title: str

Message title.

2.18.3 app.models.bookmark

Models for bookmarks of variants and genes.

class app.models.bookmark.**Bookmark**(***kwargs*)

Bookmark of a variant or gene.

id: Mapped[UUID]

UUID of the bookmark.

obj_id: str

ID of the bookmarked object.

obj_type: BookmarkTypes

Type of the bookmarked object.

user: UUID

User who created the bookmark.

2.18.4 app.models.caseinfo

Models for case information.

class app.models.caseinfo.**CaseInfo**(***kwargs*)

Case information.

affected_family_members: bool

Affected family members of the patient.

age_of_onset_month: int
Age of onset of the patient.

diseases: list[DiseaseTerm]
Diseases of the patient.

ethnicity: Ethnicity
Ethnicity of the patient.

family_segregation: bool
Family segregation of the patient.

hpo_terms: list[HpoTerm]
HPO terms of the patient.

id: Mapped[UUID]
UUID of the case information.

inheritance: Inheritance
Inheritance of the patient.

pseudonym: str
Pseudonym of the patient.

sex: Sex
Sex of the patient.

user: UUID
User who created the case information.

zygosity: Zygosity
Zygosity of the patient.

2.18.5 app.models.user

class app.models.user.**OAuthAccount**(***kwargs*)

access_token: Mapped[str]

account_email: Mapped[str]

account_id: Mapped[str]

expires_at: Mapped[int | None]

id: Mapped[UUID_ID]

oauth_name: Mapped[str]

refresh_token: Mapped[str | None]

user_id: UUID_ID

app.models.user.**TOKEN_SIZE = 65536**
Long tokens – 64kbytes should be enough for everyone

class app.models.user.**User**(***kwargs*)

email: Mapped[str]

hashed_password: Mapped[str]

id: Mapped[UUID_ID]

is_active: Mapped[bool]

is_superuser: Mapped[bool]

is_verified: Mapped[bool]

2.19 app.api.api_v1

2.19.1 app.api.api_v1.endpoints.acmgseqvar

async app.api.api_v1.endpoints.acmgseqvar.**create_acmgseqvar**(*acmgseqvar: AcmgSeqVarCreate*, *db: AsyncSession = Depends(get_db)*, *user: User = Depends(current_user_dependency)*)

Create a new ACMG Sequence Variant.

Parameters
acmgseqvar (dict or `schemas.AcmgSeqVarCreate`) – ACMG Sequence Variant to create

Returns
ACMG Sequence Variant

Return type
dict

async app.api.api_v1.endpoints.acmgseqvar.**delete_acmgseqvar**(*id: str*, *db: AsyncSession = Depends(get_db)*)

Delete a ACMG Sequence Variant by id. Available only for superusers.

Parameters
id (*uuid*) – ACMG Sequence Variant id

Returns
ACMG Sequence Variant

Return type
dict

async app.api.api_v1.endpoints.acmgseqvar.**delete_acmgseqvar_by_user**(*seqvar: str*, *db: AsyncSession = Depends(get_db)*, *user: User = Depends(current_user_dependency)*, *user_agent: str | None = None*)

Delete a ACMG Sequence Variant by id.

Parameters
id (*uuid*) – ACMG Sequence Variant id

Returns
ACMG Sequence Variant

Return type
dict

async app.api.api_v1.endpoints.acmgseqvar.**get_acmgseqvar**(*id: str*, *db: AsyncSession = Depends(get_db)*)

Get a ACMG Sequence Variant by id. Available only for superusers.

Parameters
id (*uuid*) – ACMG Sequence Variant id

Returns
ACMG Sequence Variant

Return type
dict

async app.api.api_v1.endpoints.acmgseqvar.**get_acmgseqvar_by_user**(*seqvar: str*, *db: AsyncSession = Depends(get_db)*, *user: User = Depends(current_user_dependency)*, *user_agent: str | None = None*)

Get a ACMG Sequence Variant by id.

Parameters
id (*uuid*) – ACMG Sequence Variant id

Returns
ACMG Sequence Variant

Return type
dict

Raises
HTTPException 404 – ACMG Sequence Variant not found

Note
If user_agent is browser, return 204 Response

async app.api.api_v1.endpoints.acmgseqvar.**list_acmgseqvars**(*skip: int = 0*, *limit: int = 100*, *db: AsyncSession = Depends(get_db)*)

List all ACMG Sequence Variants. Available only for superusers.

Parameters

- **skip** (*int*) – number of ACMG Sequence Variants to skip
- **limit** (*int*) – maximum number of ACMG Sequence Variants to return

Returns
list of ACMG Sequence Variants

Return type
list

async app.api.api_v1.endpoints.acmgseqvar.**list_acmgseqvars_by_user**(*skip: int = 0*, *limit: int = 100*, *db: AsyncSession = Depends(get_db)*, *user: User = Depends(current_user_dependency)*)

List ACMG Sequence Variants by user.

Returns
list of ACMG Sequence Variants

Return type
list

async app.api.api_v1.endpoints.acmgseqvar.update_acmgseqvar(*acmgseqvar: AcmgSeqVarUpdate*, *db: AsyncSession = Depends(get_db)*, *user: User = Depends(current_user_dependency)*)

Update a ACMG Sequence Variant.

Parameters
acmgseqvar (dict or schemas.AcmgSeqVarUpdate) – ACMG Sequence Variant to update

Returns
ACMG Sequence Variant

Return type
dict

2.19.2 app.api.api_v1.endpoints.adminmsgs

async app.api.api_v1.endpoints.adminmsgs.read_adminmsgs(*db: AsyncSession = Depends(get_db)*, *skip: int = 0*, *limit: int = 100*) → list[AdminMessageRead]

Retrieve all admin messages.

Returns
list of admin messages

Return type
list

2.19.3 app.api.api_v1.endpoints.auth

async app.api.api_v1.endpoints.auth.list_oauth2_providers() → list[OAuth2ProviderPublic]

Retrieve all admin messages.

Returns
list of admin messages

Return type
list

2.19.4 app.api.api_v1.endpoints.bookmarks

async app.api.api_v1.endpoints.bookmarks.**create_bookmark**(*bookmark: BookmarkCreate*, *db: AsyncSession = Depends(get_db)*, *user: User = Depends(current_user_dependency)*)

Create a new bookmark.

Parameters
: **bookmark** (dict or `schemas.BookmarkCreate`) – bookmark to create

Returns
: bookmark

Return type
: dict

async app.api.api_v1.endpoints.bookmarks.**delete_bookmark**(*id: str*, *db: AsyncSession = Depends(get_db)*)

Delete a bookmark. Available for superusers and bookmark owners.

Parameters
: **id** (*uuid*) – id of the bookmark

Returns
: bookmark which was deleted

Return type
: dict

async app.api.api_v1.endpoints.bookmarks.**delete_bookmark_for_user**(*obj_type: str*, *obj_id: str*, *db: AsyncSession = Depends(get_db)*, *user: User = Depends(current_user_dependency)*, *user_agent: str | None = None*)

Delete a bookmark for a current user by obj_type and obj_id.

Parameters

- **obj_type** (*str (enum) - "gene", "seqvar", "strucvar"*) – type of object to bookmark
- **obj_id** (*uuid*) – id of object to bookmark

Returns
: bookmark which was deleted

Return type
: dict

Raises
: **HTTPException 404** – if bookmark not found

Note
: if user_agent is browser, return 204 Response

async app.api.api_v1.endpoints.bookmarks.**get_bookmark**(*id: str*, *db: AsyncSession = Depends(get_db)*)

Get a bookmark by id. Available only for superusers.

Parameters
`id` (*uuid*) – id of the bookmark

Returns
bookmark

Return type
dict

async app.api.api_v1.endpoints.bookmarks.**get_bookmark_for_user**(*obj_type: str*, *obj_id: str*, *db: AsyncSession = Depends(get_db)*, *user: User = Depends(current_user_dependency)*, *user_agent: str | None = None*)

Get a bookmark for a current user by obj_type and obj_id.

Parameters

- **obj_type** (*str (enum) - "gene", "seqvar", "strucvar"*) – type of object to bookmark
- **obj_id** (*uuid*) – id of object to bookmark

Returns
bookmark

Return type
dict

Raises
`HTTPException 404` – if bookmark not found

Note
if user_agent is browser, return 204

async app.api.api_v1.endpoints.bookmarks.**list_bookmarks**(*skip: int = 0*, *limit: int = 100*, *db: AsyncSession = Depends(get_db)*)

List all bookmarks. Available only for superusers.

Parameters

- **skip** (*int*) – number of bookmarks to skip
- **limit** (*int*) – maximum number of bookmarks to return

Returns
list of bookmarks

Return type
list

async app.api.api_v1.endpoints.bookmarks.**list_bookmarks_for_user**(*skip: int = 0*, *limit: int = 100*, *db: AsyncSession = Depends(get_db)*, *user: User = Depends(current_user_dependency)*)

List bookmarks for a current user.

Parameters

- **skip** (*int*) – number of bookmarks to skip
- **limit** (*int*) – maximum number of bookmarks to return

Returns
list of bookmarks

Return type
list

2.19.5 app.api.api_v1.endpoints.caseinfo

async app.api.api_v1.endpoints.caseinfo.**create_caseinfo**(*caseinfo: CaseInfoCreate*, *db: AsyncSession = Depends(get_db)*, *user: User = Depends(current_user_dependency)*)

Create a new Case Information.

Parameters
caseinfo (dict or schemas.CaseInfoCreate) – Case Information to create

Returns
Case Information

Return type
dict

async app.api.api_v1.endpoints.caseinfo.**delete_caseinfo**(*id: str*, *db: AsyncSession = Depends(get_db)*)

Delete a Case Information by id. Available only for superusers.

Parameters
id (*uuid*) – Case Information id

Returns
Case Information

Return type
dict

async app.api.api_v1.endpoints.caseinfo.**delete_caseinfo_for_user**(*db: AsyncSession = Depends(get_db)*, *user: User = Depends(current_user_dependency)*)

Delete a Case Information for a current user.

Returns
Case Information

Return type
dict

async app.api.api_v1.endpoints.caseinfo.**get_caseinfo**(*id: str*, *db: AsyncSession = Depends(get_db)*)

Get a Case Information by id. Available only for superusers.

Parameters
id (*uuid*) – Case Information id

Returns
Case Information

Return type
dict

async app.api.api_v1.endpoints.caseinfo.**get_caseinfo_for_user**(*db: AsyncSession = Depends(get_db)*, *user: User = Depends(current_user_dependency)*)

Get a Case Information for a current user.

Returns
Case Information

Return type
dict

async app.api.api_v1.endpoints.caseinfo.**list_caseinfos**(*skip: int = 0*, *limit: int = 100*, *db: AsyncSession = Depends(get_db)*)

List all Case Information. Available only for superusers.

Parameters

- **skip** (*int*) – number of Case Information to skip
- **limit** (*int*) – maximum number of Case Information to return

Returns
list of Case Information

Return type
list

async app.api.api_v1.endpoints.caseinfo.**list_caseinfos_for_user**(*db: AsyncSession = Depends(get_db)*, *user: User = Depends(current_user_dependency)*)

List all Case Information for a current user.

Returns
list of Case Information

Return type
list

async app.api.api_v1.endpoints.caseinfo.**update_caseinfo_for_user**(*caseinfoupdate: CaseInfoUpdate*, *db: AsyncSession = Depends(get_db)*, *user: User = Depends(current_user_dependency)*)

Update a Case Information for a current user.

Parameters
caseinfo (dict or schemas.CaseInfoUpdate) – Case Information to update

Returns
Case Information

Return type
dict

2.19.6 app.api.api_v1.endpoints.utils

app.api.api_v1.endpoints.utils.**test_email**(*email_to: EmailStr*) → Any

Send out a test email.

Parameters
email_to (*str*) – email address to send the test email to

Returns
message

Return type
dict

2.20 app.api.internal

2.20.1 app.api.internal.endpoints.remote

Reverse proxies to external/remote services.

app.api.internal.endpoints.remote.**ACMG_RATING_KEYS: tuple[str, ...] = ('pvs1', 'ps1', 'ps2', 'ps3', 'ps4', 'pm1', 'pm2', 'pm3', 'pm4', 'pm5', 'pm6', 'pp1', 'pp2', 'pp3', 'pp4', 'pp5', 'ba1', 'bs1', 'bs2', 'bs3', 'bs4', 'bp1', 'bp2', 'bp3', 'bp4', 'bp5', 'bp6', 'bp7')**

Keys for the ACMG rating

class app.api.internal.endpoints.remote.**HTTPXClientWrapper**

Bases: object

Wrapper around HTTPX AsyncClient to for graceful startup/shutdown within FastAPI.

async_client: AsyncClient | None = None

start()

async stop()

transport: AsyncHTTPTransport | None = None

async app.api.internal.endpoints.remote.**acmg**(*request: Request*)

Implement searching for ACMG classification for SNVs and indels. Proxy requests to the WinterVar backend.

Parameters
request (fastapi.Request) – request

Returns
ACMG classification

Return type
dict

async app.api.internal.endpoints.remote.**cnv_acmg**(*request: Request*)

Implement searching for ACMG classification for CNVs. Proxy requests to the wAutoCNV backend.

Parameters
request (fastapi.Request) – request

Returns
ACMG classification

Return type
dict

`app.api.internal.endpoints.remote.`**`default_acmg_rating`**`()` → dict[str, bool]

`async app.api.internal.endpoints.remote.`**`pubtator3_api`**`(`*request: Request*, *path: str*`)`

Proxy requests to the PubTator 3 backend.

Parameters

- **request** (`fastapi.Request`) – request
- **path** (*str*) – path to append to the backend URL

Returns
response

Return type
`fastapi.responses.StreamingResponse`

`async app.api.internal.endpoints.remote.`**`variantvalidator`**`(`*request: Request*, *path: str*`)`

Implement VariantValidator API. Proxy requests to the VariantValidator backend.

Parameters

- **request** (`fastapi.Request`) – request
- **path** (*str*) – path to append to the backend URL

Returns
response

Return type
`fastapi.responses.StreamingResponse`

PYTHON MODULE INDEX

a

app.api.api_v1.endpoints.acmgseqvar, 60
app.api.api_v1.endpoints.adminmsgs, 62
app.api.api_v1.endpoints.auth, 62
app.api.api_v1.endpoints.bookmarks, 63
app.api.api_v1.endpoints.caseinfo, 65
app.api.api_v1.endpoints.utils, 67
app.api.internal.endpoints.remote, 67
app.models.acmgseqvar, 57
app.models.adminmsg, 58
app.models.bookmark, 58
app.models.caseinfo, 58
app.models.user, 59

INDEX

A

access_token (*app.models.user.OAuthAccount attribute*), 59
account_email (*app.models.user.OAuthAccount attribute*), 59
account_id (*app.models.user.OAuthAccount attribute*), 59
acmg() (*in module app.api.internal.endpoints.remote*), 67
acmg_rank (*app.models.acmgseqvar.AcmgSeqVar attribute*), 57
ACMG_RATING_KEYS (*in module app.api.internal.endpoints.remote*), 67
AcmgSeqVar (*class in app.models.acmgseqvar*), 57
active_start (*app.models.adminmsg.AdminMessage attribute*), 58
active_stop (*app.models.adminmsg.AdminMessage attribute*), 58
AdminMessage (*class in app.models.adminmsg*), 58
affected_family_members (*app.models.caseinfo.CaseInfo attribute*), 58
age_of_onset_month (*app.models.caseinfo.CaseInfo attribute*), 58
app.api.api_v1.endpoints.acmgseqvar
    module, 60
app.api.api_v1.endpoints.adminmsgs
    module, 62
app.api.api_v1.endpoints.auth
    module, 62
app.api.api_v1.endpoints.bookmarks
    module, 63
app.api.api_v1.endpoints.caseinfo
    module, 65
app.api.api_v1.endpoints.utils
    module, 67
app.api.internal.endpoints.remote
    module, 67
app.models.acmgseqvar
    module, 57
app.models.adminmsg
    module, 58
app.models.bookmark
    module, 58
app.models.caseinfo
    module, 58
app.models.user
    module, 59
async_client (*app.api.internal.endpoints.remote.HTTPXClientWrapper attribute*), 67

B

Bookmark (*class in app.models.bookmark*), 58

C

CaseInfo (*class in app.models.caseinfo*), 58
cnv_acmg() (*in module app.api.internal.endpoints.remote*), 67
create_acmgseqvar() (*in module app.api.api_v1.endpoints.acmgseqvar*), 60
create_bookmark() (*in module app.api.api_v1.endpoints.bookmarks*), 63
create_caseinfo() (*in module app.api.api_v1.endpoints.caseinfo*), 65

D

default_acmg_rating() (*in module app.api.internal.endpoints.remote*), 68
delete_acmgseqvar() (*in module app.api.api_v1.endpoints.acmgseqvar*), 60
delete_acmgseqvar_by_user() (*in module app.api.api_v1.endpoints.acmgseqvar*), 60
delete_bookmark() (*in module app.api.api_v1.endpoints.bookmarks*), 63
delete_bookmark_for_user() (*in module app.api.api_v1.endpoints.bookmarks*), 63
delete_caseinfo() (*in module app.api.api_v1.endpoints.caseinfo*), 65
delete_caseinfo_for_user() (*in module app.api.api_v1.endpoints.caseinfo*), 65
diseases (*app.models.caseinfo.CaseInfo attribute*), 59

E

email (*app.models.user.User attribute*), 59

enabled (*app.models.adminmsg.AdminMessage attribute*), 58
ethnicity (*app.models.caseinfo.CaseInfo attribute*), 59
expires_at (*app.models.user.OAuthAccount attribute*), 59

F

family_segregation (*app.models.caseinfo.CaseInfo attribute*), 59

G

get_acmgseqvar() (*in module app.api.api_v1.endpoints.acmgseqvar*), 61
get_acmgseqvar_by_user() (*in module app.api.api_v1.endpoints.acmgseqvar*), 61
get_bookmark() (*in module app.api.api_v1.endpoints.bookmarks*), 63
get_bookmark_for_user() (*in module app.api.api_v1.endpoints.bookmarks*), 64
get_caseinfo() (*in module app.api.api_v1.endpoints.caseinfo*), 65
get_caseinfo_for_user() (*in module app.api.api_v1.endpoints.caseinfo*), 65

H

hashed_password (*app.models.user.User attribute*), 60
hpo_terms (*app.models.caseinfo.CaseInfo attribute*), 59
HTTPXClientWrapper (*class in app.api.internal.endpoints.remote*), 67

I

id (*app.models.acmgseqvar.AcmgSeqVar attribute*), 57
id (*app.models.adminmsg.AdminMessage attribute*), 58
id (*app.models.bookmark.Bookmark attribute*), 58
id (*app.models.caseinfo.CaseInfo attribute*), 59
id (*app.models.user.OAuthAccount attribute*), 59
id (*app.models.user.User attribute*), 60
inheritance (*app.models.caseinfo.CaseInfo attribute*), 59
is_active (*app.models.user.User attribute*), 60
is_superuser (*app.models.user.User attribute*), 60
is_verified (*app.models.user.User attribute*), 60

L

list_acmgseqvars() (*in module app.api.api_v1.endpoints.acmgseqvar*), 61
list_acmgseqvars_by_user() (*in module app.api.api_v1.endpoints.acmgseqvar*), 61
list_bookmarks() (*in module app.api.api_v1.endpoints.bookmarks*), 64
list_bookmarks_for_user() (*in module app.api.api_v1.endpoints.bookmarks*), 64
list_caseinfos() (*in module app.api.api_v1.endpoints.caseinfo*), 66
list_caseinfos_for_user() (*in module app.api.api_v1.endpoints.caseinfo*), 66
list_oauth2_providers() (*in module app.api.api_v1.endpoints.auth*), 62

M

module
- app.api.api_v1.endpoints.acmgseqvar, 60
- app.api.api_v1.endpoints.adminmsgs, 62
- app.api.api_v1.endpoints.auth, 62
- app.api.api_v1.endpoints.bookmarks, 63
- app.api.api_v1.endpoints.caseinfo, 65
- app.api.api_v1.endpoints.utils, 67
- app.api.internal.endpoints.remote, 67
- app.models.acmgseqvar, 57
- app.models.adminmsg, 58
- app.models.bookmark, 58
- app.models.caseinfo, 58
- app.models.user, 59

O

oauth_name (*app.models.user.OAuthAccount attribute*), 59
OAuthAccount (*class in app.models.user*), 59
obj_id (*app.models.bookmark.Bookmark attribute*), 58
obj_type (*app.models.bookmark.Bookmark attribute*), 58

P

pseudonym (*app.models.caseinfo.CaseInfo attribute*), 59
pubtator3_api() (*in module app.api.internal.endpoints.remote*), 68

R

read_adminmsgs() (*in module app.api.api_v1.endpoints.adminmsgs*), 62
refresh_token (*app.models.user.OAuthAccount attribute*), 59

S

seqvar_name (*app.models.acmgseqvar.AcmgSeqVar attribute*), 57
sex (*app.models.caseinfo.CaseInfo attribute*), 59
start() (*app.api.internal.endpoints.remote.HTTPXClientWrapper method*), 67
stop() (*app.api.internal.endpoints.remote.HTTPXClientWrapper method*), 67

T

test_email() (*in module app.api.api_v1.endpoints.utils*), 67
text (*app.models.adminmsg.AdminMessage attribute*), 58

title (*app.models.adminmsg.AdminMessage attribute*), 58
TOKEN_SIZE (*in module app.models.user*), 59
transport (*app.api.internal.endpoints.remote.HTTPXClientWrapper attribute*), 67

U

update_acmgseqvar() (*in module app.api.api_v1.endpoints.acmgseqvar*), 62
update_caseinfo_for_user() (*in module app.api.api_v1.endpoints.caseinfo*), 66
user (*app.models.acmgseqvar.AcmgSeqVar attribute*), 57
user (*app.models.bookmark.Bookmark attribute*), 58
user (*app.models.caseinfo.CaseInfo attribute*), 59
User (*class in app.models.user*), 59
user_id (*app.models.user.OAuthAccount attribute*), 59

V

variantvalidator() (*in module app.api.internal.endpoints.remote*), 68

Z

zygosity (*app.models.caseinfo.CaseInfo attribute*), 59
