# Supplementary material for: REEV: review, evaluate and explain variants
Source: Nucleic Acids Res. 2024 May 20;52(W1):W148–58. doi: 10.1093/nar/gkae366 (PMC11223839; doi:10.1093/nar/gkae366)
Supplement: gkae366_Supplemental_Files [file gkae366_supplemental_files.zip › Supplementary-Information.docx]

**TABLE AND FIGURES LEGENDS**

**Supplementary Table 1. Variants for REEV software testing.**

**Supplementary Figure 1.** **REEV frontend overview of sequence variants. A** Overview of the REEV startpage: (1) Field for entering variant of interest, (2) Case Info button to provide case-specific information, e.g. phenotypic information as HPO terms, (3) Login, (4) Further information (About, Contact, Privacy Policy, Terms of Use, Data Versions). **B-C** Left: menu and quick link to the different sections; right: respective sections on gene (**B**) and variant (**C**) level.

**Supplementary Figure 2.** **REEV frontend overview of structural variants.** Left: menu and quick link to the different sections; right: respective sections on variant level. Note: Gene level information is shown in the same vein as for sequence variants (depicted in ***Supplementary Figure 1B***).

**Supplementary Figure 3.** **REEV software testing results.** Comparison of time (in minutes) to final ACMG classification between using REEV (red) and single look-up (grey) of required information: **A** for small variants and **B** structural variants (statistics: one-sided paired t-test, α=0.05; n=12 (6 software testers à 2 different variants for every type of variant).
