# Supplementary figures and images for: REEV: review, evaluate and explain variants
Source: Nucleic Acids Res. 2024 May 20;52(W1):W148–58. doi: 10.1093/nar/gkae366 (PMC11223839; doi:10.1093/nar/gkae366)

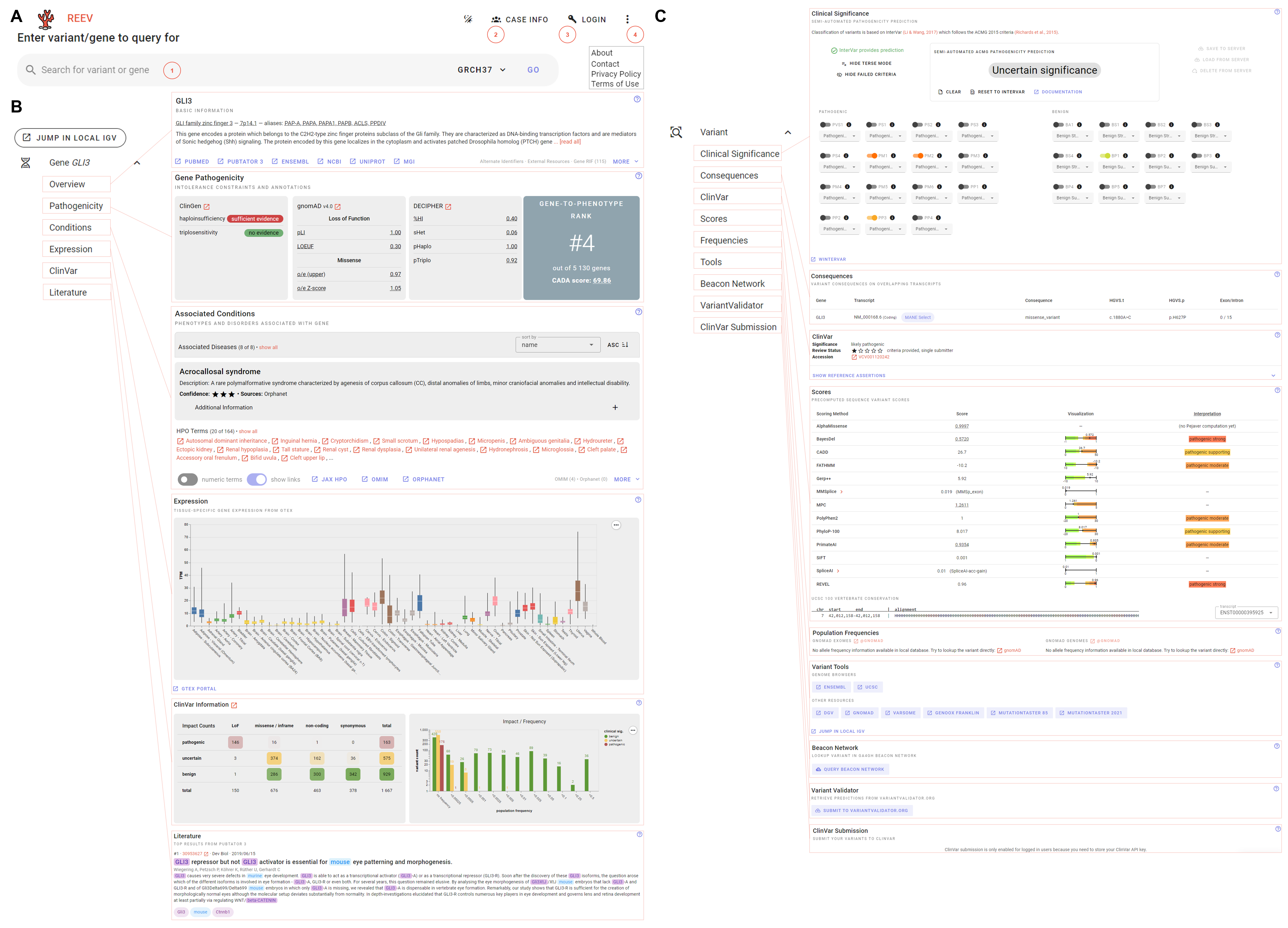

Supplement: gkae366_Supplemental_Files [file gkae366_supplemental_files.zip › SupplFig1.png]

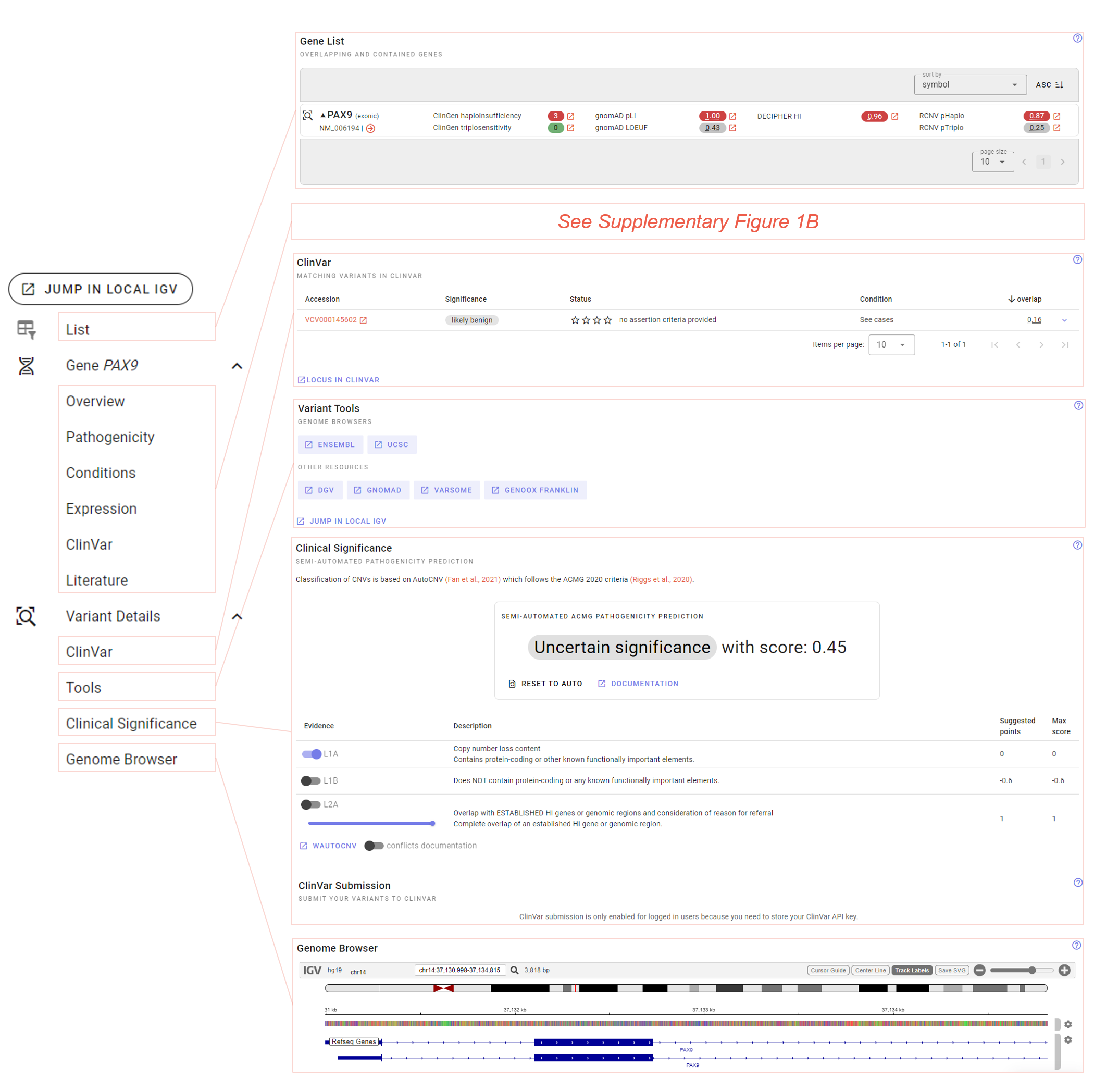

Supplement: gkae366_Supplemental_Files [file gkae366_supplemental_files.zip › SupplFig2.png]

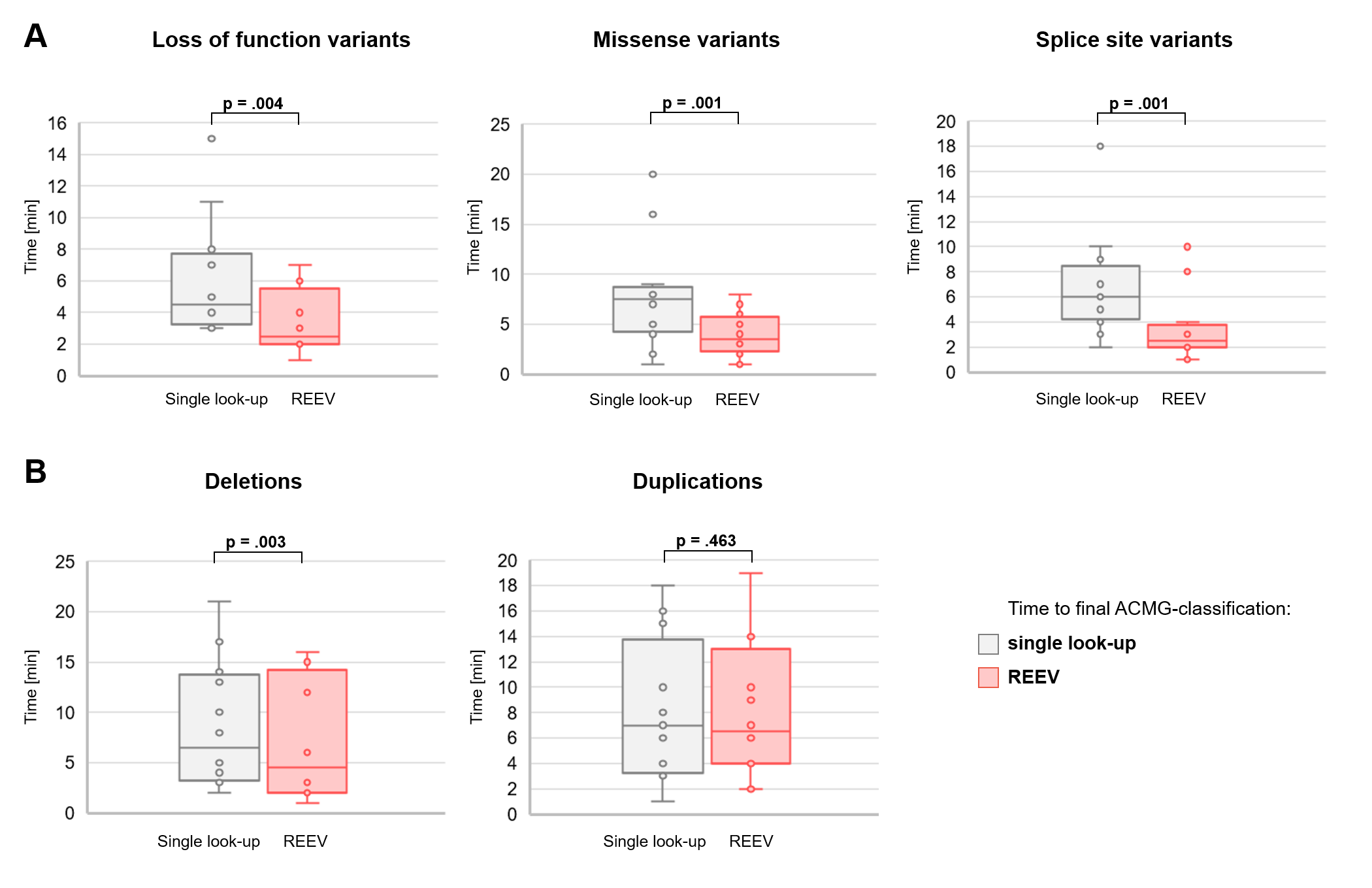

Supplement: gkae366_Supplemental_Files [file gkae366_supplemental_files.zip › SupplFig3.png]
